# Supplementary material for: Whisker-based tactile flight for tiny drones
Source: Nat Commun. 2026 Sep 18;17:9743. doi: 10.1038/s41467-026-77366-7 (PMC13588785; doi:10.1038/s41467-026-77366-7)
Supplement: Supplementary file 1 [file 41467_2026_77366_MOESM12_ESM.pdf]

nature portfolio

# Peer Review File

# Whisker-based Tactile Flight for Tiny Drones

Corresponding Author: Professor Salua Hamaza

**This file contains all reviewer reports in order by version, followed by all author rebuttals in order by version.**

Version 0:

Reviewer comments:

Reviewer #3

(Remarks to the Author)
Summary:

In this work, the authors present a mechatronic design to enable whisker-based tactile sensing on drones. They target very lightweight quadrotors (~30g), hence the additional mass and torque generated by the whisker mechanism is a major concern. The authors use two 20cm nitinol wires that are interfaced with 3 pressure sensors each, to form two whiskers. The whiskers are pointed in the forward direction, and the authors investigate the question about what angle the whiskers should be at by trying several settings from pointing downwards to pointing upwards. They find that an angle of 45 degrees yields the best results in their experiments because, being pointed upwards, the upwards torque counterbalances the additional weight that is shifted towards the front.
The goal of the authors is to measure the distance to static obstacles using the whiskers. But the barometric sensors cannot directly measure the bending force, let alone the distance.
Since the barometric sensors do not directly output the distance but rather a noisy and biased proxy for the bending force, the authors propose using a (temperature-based) bias estimator, a Butterworth filter to filter out noise, and a neural network to predict distance based on the three filtered barometer readings for each whisker.
The distance prediction is then used as observations for a Kalman filter that estimates the distance and angle to the wall. They find that they can estimate the distances with about 4-7 mm error and orientation with about 7 degrees.
The authors demonstrate this by conducting real-world test flights that sweep along solid and soft surfaces using a simple control logic that is based on the estimations of the whisker pipeline.
Furthermore, the authors integrate their sensor into a Gaussian Process-based exploration algorithm and combine it with an exploration policy that avoids concave regions by interpolating between whisker contact observations (located in space through the optical flow deck).

Remarks:

1. "For real-world validation, we used the Crazyflie 2.1 platform": Should be clear if it is the brushless version (Figure 1) or the brushed version (text, some of the videos?)
2. "due to its limited thrust." How much thrust?
3. "Five whisker placement angles (–15∘, 0∘, 15∘, 30∘, 45∘) were tested at a contact depth d = 3 cm, sweeping along a smooth wall at 0.1, 0.2, and 0.5 m/s (Fig. 2b–f). Only the 45∘ placement remained stable across all velocities, while 15∘, which shifted close to 0∘ due to gravity," The best angle is at the boundary of the tested range. To motivate the design choice, a decreasing performance beyond the chosen value should be shown (local optimum).
4. The videos were not playable with QuickTime, only VLC works.
5. How does it deal with the heavy oscillations that can be seen in the video, after sliding off the obstacle?
6. Some experiments seem to be conducted with the Crazyflie Brushed and others with the Brushless?
7. It seems like the angle tests are N=1 sample size? That does not give us conclusions about the reliability.
8. "F/T" not specified, I only realized on the third read that it is force/torque.
9. Crazyflie weight specifications in the text contradict Figure 1a in multiple places.
10. "An identical protector is mounted on the drone's tail to provide symmetry and stability, with the battery slightly shifted backward to balance the center of mass." contradicts that the "minimizing the need for rear counterweight."
11. "To suppress hysteresis and ringing, we apply a first-order Butterworth bandpass filter (0.05–1 Hz), implemented as:" How can a band-pass filter suppress hysteresis?

12. Neural network seems overkill for a 3D => 1D regression. Linear regression should at least be provided as a baseline.
13. In my opinion, reiterating basics about neural networks or the Kalman filter do not add to the contribution of this work. Naturally, they have to be severely limited in scope.
14. It is not clear if the data in table 1 is purely the test set or also contains training samples.
15. In the exploration scenario, the algorithm seems to heavily rely on the optical flow sensor (which works very well out of the box and has low drift, in my experience). So it is not really clear how these experiments contribute to this work.
16. The exploration in particular seems to be a stack of many heuristics/design decisions that are not properly ablated.
17. The avoidance of concave regions is not clearly demonstrated.

Conclusion:

This work proposes an interesting mechatronic and signal-processing method to add tactile sensing to aerial vehicles. The whisker-based design is shown to work in the real world, but many of its aspects and design decisions are not ablated or justified well. It is an interesting initial exploration for this type of sensor, but e.g. it is not clear to me why using barometric sensors is the best choice. Due to the novelty, there seems to be no baseline to compare it to, but it would still be valuable if the authors could elaborate more on what went into that design decision. Including what alternatives were considered and how it can be improved in the future.

(Remarks on code availability)
I have briefly inspected the codebase (I don't have a compatible machine to run it available right now). It seems like the code for the method described in this paper is not yet included. I found the dataset and the simulator used in this work. The repo states "Note: The full source code will be released upon acceptance of the article." so I assume the relevant code is not available.

Reviewer #4

(Remarks to the Author)
The paper presents a whisker-based tactile sensing system for tiny drones. The authors design a barometer-based whisker apparatus and integrate it on a Crazyflie platform. The system enables wall-following, navigation, and exploration using touch as the primary sensing modality, including operation in complete darkness.

The paper presents clear novelty by enabling tactile flight on an ultra-light drone with fully onboard execution under strict memory and computation constraints. It demonstrates strong system integration, combining hardware design, drift compensation, learning-based depth estimation, filtering, control, and exploration into a coherent pipeline. The experimental validation is convincing and comprehensive. The work also has strong practical relevance, addressing failure modes of vision and laser sensing, such as darkness and transparency, with particularly compelling demonstrations in complete darkness. That said, the following points may help further strengthen the paper.

Comments:
1. While related work is cited, there is no direct experimental comparison with other aerial tactile approaches, such as compliant bumpers. It is therefore difficult to quantify the improvement in stability or perception quality. Can the authors provide a quantitative comparison between whisker sensing and collision-resilient bumper-based drones in terms of stability, impact force, and perception capabilities?
2. What is the exact MLP architecture used for depth estimation? How many parameters does it contain?
3. How sensitive is the model to whisker replacement or barometer variability? The paper mentions calibration challenges. Is per-device retraining required?
4. In active exploration, what is the computational cost of GPIS onboard? How close is the system to memory limits during exploration?
5. The process model assumes static obstacles and locally planar surfaces. How does performance degrade with curved or moving objects?
6. It would be helpful to report energy consumption or flight time reduction due to whisker payload and computation.

(Remarks on code availability)

Version 1:

Reviewer comments:

Reviewer #3

(Remarks to the Author)
I would like to thank the authors for thoroughly addressing my comments! I can confirm that all of my concerns have been addressed, and I believe that this work is a good contribution to the community.

(Remarks on code availability)

Reviewer #4

(Remarks to the Author)
The manuscript has been substantially improved after revision, and the authors have adequately addressed all of the comments. Therefore, I would recommend acceptance.

(Remarks on code availability)
The released code may provide a useful resource for the community. However, reproducibility may still depend on factors beyond the software itself, such as hardware fabrication and sensor recalibration.

**Open Access** This Peer Review File is licensed under a Creative Commons Attribution 4.0 International License, which permits use, sharing, adaptation, distribution and reproduction in any medium or format, as long as you give appropriate credit to the original author(s) and the source, provide a link to the Creative Commons license, and indicate if changes were made.
In cases where reviewers are anonymous, credit should be given to 'Anonymous Referee' and the source.
The images or other third party material in this Peer Review File are included in the article's Creative Commons license, unless indicated otherwise in a credit line to the material. If material is not included in the article's Creative Commons license and your intended use is not permitted by statutory regulation or exceeds the permitted use, you will need to obtain permission directly from the copyright holder.
To view a copy of this license, visit https://creativecommons.org/licenses/by/4.0/

# Response to Reviewers

Manuscript title: *Whisker-based Tactile Flight for Tiny Drones*

We sincerely thank the Editor and the Reviewers for their careful reading of our manuscript and for their constructive comments. We have thoroughly revised the manuscript. Below, we provide a point-by-point response to each comment, as well as a preview of the changes reported to the manuscript. Reviewer comments are shown in blue underlined text, and revisions made in the manuscript are shown in orange text.

## Reviewer #3

### Summary:

In this work, the authors present a mechatronic design to enable whisker-based tactile sensing on drones. They target very lightweight quadrotors ( 30g), hence the additional mass and torque generated by the whisker mechanism is a major concern. The authors use two 20cm nitinol wires that are interfaced with 3 pressure sensors each, to form two whiskers. The whiskers are pointed in the forward direction, and the authors investigate the question about what angle the whiskers should be at by trying several settings from pointing downwards to pointing upwards. They find that an angle of 45 degrees yields the best results in their experiments because, being pointed upwards, the upwards torque counterbalances the additional weight that is shifted towards the front. The goal of the authors is to measure the distance to static obstacles using the whiskers. But the barometric sensors cannot directly measure the bending force, let alone the distance. Since the barometric sensors do not directly output the distance but rather a noisy and biased proxy for the bending force, the authors propose using a (temperature-based) bias estimator, a Butterworth filter to filter out noise, and a neural network to predict distance based on the three filtered barometer readings for each whisker. The distance prediction is then used as observations for a Kalman filter that estimates the distance and angle to the wall. They find that they can estimate the distances with about 4-7 mm error and orientation with about 7 degrees. The authors demonstrate this by conducting real-world test flights that sweep along solid and soft surfaces using a simple control logic that is based on the estimations of the whisker pipeline. Furthermore, the authors integrate their sensor into a Gaussian Process-based exploration algorithm and combine it with an exploration policy that avoids concave regions by interpolating between whisker contact observations (located in space through the optical flow deck).

**Response:**
We thank Reviewer 3 for carefully reading our manuscript and for the detailed and constructive comments. We appreciate the reviewer's clear summary of the overall system and the recognition that the main technical challenges arise from integrating whisker-based tactile sensing on a very lightweight aerial platform. Below, we address each point in detail. We note that Comments 1, 2,

3, 6, and 7 concern closely related issues, so we place these responses first for clarity.

## Comment 1

**Reviewer comment:**
"For real-world validation, we used the Crazyflie 2.1 platform": Should be clear if it is the brushless version (Figure 1) or the brushed version (text, some of the videos?)

**Response:**
Thank you for pointing out this ambiguity. The reviewer is correct that the platform designation should be made explicit in the manuscript. We would like to clarify that, in the official Bitcraze naming convention, "Crazyflie 2.1" refers to the standard brushed platform [1], whereas "Crazyflie 2.1 Brushless" refers to the brushless variant [2]. Thus, our original use of "Crazyflie 2.1" followed the official platform designation.

That said, we agree this was not sufficiently clear to the reader. As further explained in our response to Comment 6, we have conducted new experiments related to the whisker placement angle on the Crazyflie 2.1 Brushless platform. These new experiments have replaced the old ones in the Results section. The old experiments were indeed conducted on the Crazyflie 2.1 brushed, and have now been moved to the Supplementary Materials along with the supporting observations. We have also revised the text, all figure captions, and the Supplementary Materials to explicitly label the brushed platform as Crazyflie 2.1 brushed wherever relevant.

**Revision in the paper:**
Page 7, Results, lines 136–137:
Preliminary experimental observations obtained on the Crazyflie 2.1 brushed platform are included in the Supplementary Materials. Video compilations are provided in Movie S7.

Page 21, Methods, lines 402 – 408:

### Hardware

For all experiments reported in the main text, we utilized a prototype Crazyflie 2.1 Brushless drone. The drone features 4 × 08028-10000KV high-quality motors and, with a measured weight of 24.2 g for the drone and 9.2 g for the 350 mAh battery (33.4 g total for the bare platform and battery), achieves a flight endurance of over 10 minutes. We equipped the drone with two whiskers mounted 5 cm apart on a custom-designed 3D-printed mounting. For the whisker placement-angle study, the mounting included holders that allowed different angular orientations of the whiskers to be tested.

Page S6, Supplementary Text, lines 886 – 900:

### Preliminary experiments of whisker placements on the Crazyflie 2.1 brushed platform

We conducted preliminary experiments of whisker placement angle on the Crazyflie 2.1 brushed platform to qualitatively assess how the contact location in space influences the interaction stability during wall sweeping maneuvers.

The brushed platform has a mass of 27 g and a recommended maximum payload of about 15 g. Due to its lower actuation capability, this platform made less favourable whisker placements more evident, as it was more prone to crashes due to contact-induced instabilities.

In these preliminary trials, five whisker placement angles ($-15°$, $0°$, $15°$, $30°$, and $45°$) were tested at a contact depth of $d = 3$ cm while sweeping along a smooth wall at 0.1, 0.2, and 0.5 m/s (Fig. S3b–g). Among the tested configurations, the $45°$ placement showed the most stable interaction across the tested velocities, whereas the $15°$ configuration, which shifted close to $0°$ due to gravity, showed the highest crash tendency. Crashes were primarily associated with excessive yaw moment deviation that the controller could not compensate in time. Video compilations are also provided in Movie S1.

Movie S1 and S7:
We have revised the labels in Movie S1 and S7 to explicitly distinguish the two platforms. The brushless trials are now labeled as "Crazyflie 2.1 Brushless", and the earlier exploratory brushed trials are labeled as "Crazyflie 2.1 brushed".

## Comment 2

**Reviewer comment:**
"due to its limited thrust." How much thrust?

**Response:**
According to the Crazyflie supplier, the crazyflie brushed can generate up to approximately 57.9 g of total thrust at 93.75% PWM [3]. However, this corresponds to near-maximum operating conditions; in practice, only part of this thrust is available for flying, while retaining some buffer for attitude stabilization, disturbance rejection, and battery-voltage variation. We also observed experimentally that, as the payload approached this limit, takeoff and stable flight became difficult.

In the revised manuscript, the main whisker placement-angle study is now reported on the Crazyflie 2.1 Brushless platform rather than the brushed platform. We therefore removed the "limited thrust" statement from the main text. The payload details for the old experiments are now provided in the Supplementary Materials.

**Revision in the paper:**
Page S6, Supplementary Text, lines 891–893:
The brushed platform has a mass of 27 g and a recommended maximum payload of about 15 g. Its smaller practical thrust margin made less favourable whisker placements more evident, as the

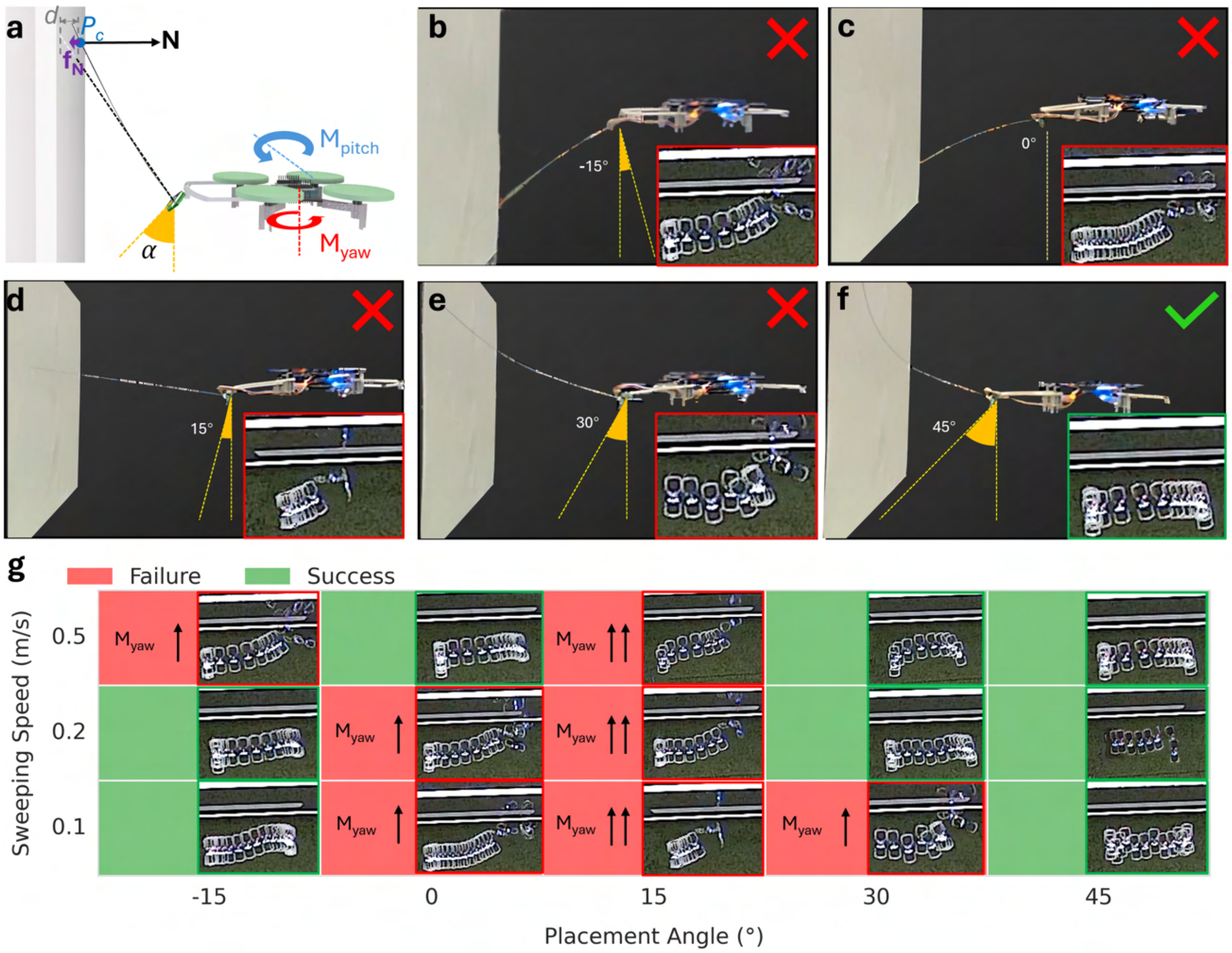


Figure S3: Effect of whisker placement angle on drone stability during wall-sweeping interactions on the Crazyflie 2.1 brushed platform. (a) Schematic illustration of the forces and moments acting on the drone during whisker sweeping. During contact with a vertical obstacle, the whisker generates a normal force $N$, which induces a friction force $f_N$. The placement angle $\alpha$ influences the resulting pitch moment $M_{\text{pitch}}$ and yaw moment $M_{\text{yaw}}$. (b–f) Representative wall-sweeping trials with whiskers mounted at $-15°$, $0°$, $15°$, $30°$, and $45°$ in the pitch plane. Due to gravity, the tip of the whisker naturally tilts downward by approximately $10°$ from the preset angle. The top-view inset in the lower right corner illustrates that crashes primarily occur when the drone is unable to compensate for $M_{\text{yaw}}$. (g) Exploratory results showing yaw deflection and success or failure outcomes for different whisker placement angles at sweeping velocities of 0.1, 0.2, and 0.5 m/s. Among the tested configurations, the $45°$ placement showed the most stable interaction in these preliminary trials.

platform was more prone to crashes caused by contact-induced instabilities.

## Comment 3

**Reviewer comment:**
"Five whisker placement angles (−15°, 0°, 15°, 30°, 45°) were tested at a contact depth $d = 3$ cm, sweeping along a smooth wall at 0.1, 0.2, and 0.5 m/s (Fig. 2b–f). Only the 45° placement remained stable across all velocities, while 15°, which shifted close to 0° due to gravity," The best angle is at the boundary of the tested range. To motivate the design choice, a decreasing performance beyond the chosen value should be shown (local optimum).

**Response:**
Thank you for this helpful suggestion. We agree and we thus have extended the angle range for this investigation, to include a 60° configuration. Moreover, we have re-done all placement experiments on the Crazyflie 2.1 Brushless platform.

Specifically, we evaluated six whisker placement angles (−15°, 0°, 15°, 30°, 45°, and 60°) at three sweeping velocities (0.1, 0.2, and 0.5 m/s) and a contact depth of $d = 8$ cm, with four repeated trials per angle–velocity condition, resulting in 72 experiments in total. We selected $d = 8$ cm because it ensures a safety distance from the wall while still producing clear whisker contact.

We quantified performance using both the "crash rate" and the "peak absolute yaw deviation" during contact. We used peak absolute yaw deviation because, under the same controller, it provides a direct measure of the largest contact-induced attitude disturbance that must be compensated during wall sweeping, and therefore serves as a practical indicator of interaction stability.

The revised results show that the 30°, 45°, and 60° configurations satisfy contact flight without crashes, but the 45° placement yielded the smallest yaw drift across all speeds. Although the mechanical analysis predicts a reduction in normal force with increasing whisker angle (page S3, **Theoretical analysis for whisker placement selection**), the 60° configuration did not further reduce the observed yaw drift. A likely explanation for this is that at 60°, the normal distance of the drone from the wall is significantly shortened, inducing aerodynamic disturbances on the drone due to its proximity. Thus, while 60° remained crash-free, it did not outperform 45° in practice and also reduced the usable sensing range. We therefore selected 45° for the final design because it provided the best balance between interaction stability and usable sensing range.

We also agree that our original wording, *"optimal whisker placement"*, was too strong in this context. In the revised manuscript, we replaced such wording with more appropriate phrasing.

The revised Fig. 2 and the corresponding Results text have been updated accordingly. The repeatability of these experiments is further detailed in our response to Comment 7.

**Revision in the paper:**
Page 7, Results, lines 119–137 and Figure 2:

For real-world validation, we conducted experiments on the Crazyflie 2.1 Brushless platform, a

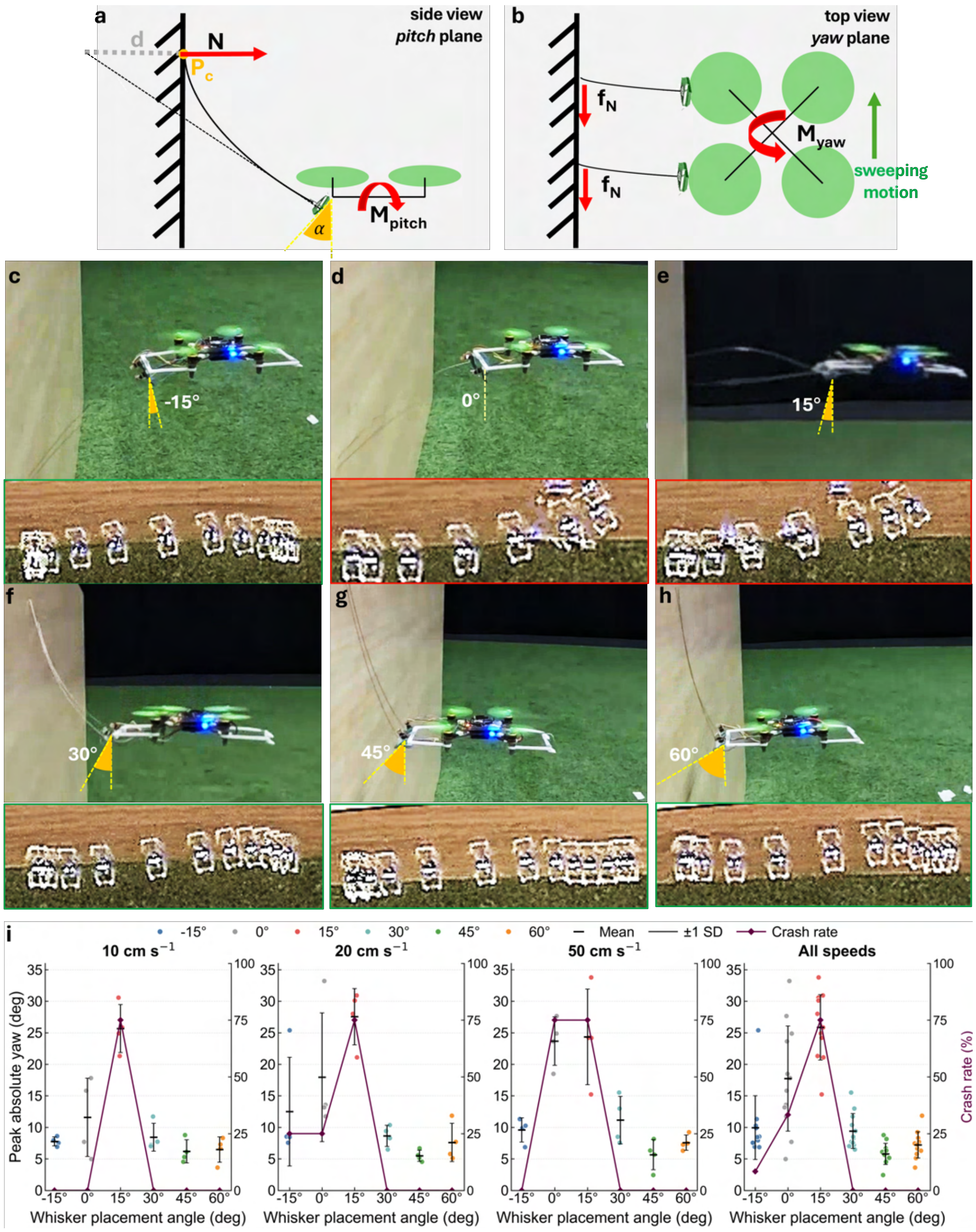


Figure 2: Effect of the whisker placement angle on the drone's stability during wall-following interactions. (a-b) Schematic illustration of forces and moments present on the drone during whisker-based wall sweeping at the positive placement angle. While in contact with a vertical obstacle, the whisker generates a normal force $N$ which induces a friction force $f_N$. The whisker's placement angle $\alpha$ influences the resulting $N$ and moment arm, and thus the drone's pitch $M_{pitch}$ and yaw $M_{yaw}$ moments. (c–h) Experimental setup, whisker deflection, and sweeping motion of the drone with whiskers mounted at $-15°$, $0°$, $15°$, $30°$, $45°$, and $60°$ in the pitch plane. Due to gravity, the whisker naturally tilts downward by approximately $10°$ from the preset angle. In the top-view clips we can observe sweeping behaviour at 0.5 m/s: crashes primarily occur due to the drone's yaw deviation and inability compensate for $M_{yaw}$. (i) Peak absolute yaw deviation and crash rate for the six placement angles at sweeping velocities of 0.1, 0.2, and 0.5 m/s. Each angle–velocity condition was repeated four times. The 45° placement yielded the smallest yaw deviation across all speeds, as well as no crashes.

44.1-g drone equipped with two whiskers. We evaluated six whisker placement angles ($-15°$, $0°$, $15°$, $30°$, $45°$, and $60°$) at a contact depth of $d = 8$ cm while sweeping along a smooth wall at 0.1, 0.2, and 0.5 m/s (Fig. 2c–h). Each angle–velocity condition was repeated four times, resulting in 72 trials in total. We quantified stability using the peak absolute yaw deviation during contact and the crash rate (Fig. 2i). Across all speeds, the 15° configuration showed the largest yaw drift and the highest crash rate, consistent with the whisker drooping toward a near-0° configuration under gravity. The 0° configuration also showed large yaw excursions and frequent crashes. The $-15°$ configuration produced one crash, consistent with the additional adverse pitch moment predicted by the analysis above. In contrast, the 30°, 45°, and 60° configurations completed all trials without crashes. Among these, the 45° placement yielded the smallest mean peak yaw deviation across speeds, indicating the most stable wall interaction (Fig. 2i). Although the 60° configuration also remained crash-free, it did not further reduce yaw drift and shortened the effective forward sensing range of the whiskers. One possible reason is that, at 60°, the shortened forward reach brings the drone body closer to the wall at the tested contact depth, which may introduce additional aerodynamic-related disturbances. We therefore selected 45° as the whisker placement angle because it provided the best balance between interaction stability and usable sensing range. Earlier exploratory observations obtained on the Crazyflie 2.1 Brushed platform are included in the Supplementary Materials. Video compilations are provided in Movie S1 and S7.

Page 4, Results, lines 78–80:
We begin by detailing the construction of the whiskered drone platform, which involves designing and fabricating the whiskers, selecting the appropriate drone, and determining a suitable whisker placement angle.

Page S4, Supplementary Text, lines 857:

**Theoretical analysis for whisker placement selection**

Page 21, Methods, lines 408 – 409:
For the subsequent experiments, the whiskers were mounted at the selected 45° placement angle.

### Comment 6

**Reviewer comment:**
Some experiments seem to be conducted with the Crazyflie Brushed and others with the Brushless?

**Response:**
Thank you for pointing this out. The reviewer is correct that the original manuscript used different Crazyflie variants in different parts of the study, which could cause confusion. In the original version, the earlier whisker placement-angle trials were conducted on the Crazyflie 2.1 brushed platform because its lower actuation margin made crash-prone configurations easier to reveal. However, we agree that this created an inconsistency in platform usage across the manuscript.

As noted above, we have addressed this by moving the earlier Crazyflie 2.1 brushed results to the Supplementary Materials as supporting observations only, and by repeating the whisker placement-angle study on the Crazyflie 2.1 Brushless platform for the revised main text. In addition, the revised angle-placement study is now evaluated using both the crash rate and the peak absolute yaw deviation during contact, rather than crash outcomes alone, providing a more complete assessment of interaction stability. Further clarification of the platform naming is provided in our response to Comment 1.

As noted above, these changes have already been explained in our responses; for clarity, we present below the consolidated additions and relocations of the Crazyflie 2.1 Brushless and Crazyflie 2.1 brushed content.

**Revision in the paper:**
Page 7, Results, lines 119–137 and Figure 2:
Same as in response to Comment 3.

Page 21, Methods, lines 402 – 408:

### Hardware

Same as in response to Comment 1.

Page S6, Supplementary Text, lines 886 – 900:

### Preliminary experiments of whisker placements on the Crazyflie 2.1 brushed platform

Same as in response to Comment 1.

### Comment 7

**Reviewer comment:**
It seems like the angle tests are N=1 sample size? That does not give us conclusions about the reliability.

**Response:**
Thank you for highlighting this. We agree that the original manuscript did not sufficiently establish the repeatability of the angle-placement results. In the revised manuscript, we repeated the whisker placement-angle experiment on the Crazyflie 2.1 Brushless platform four times for each angle–velocity condition.

Specifically, we evaluated six whisker placement angles (−15°, 0°, 15°, 30°, 45°, and 60°) at three sweeping velocities (0.1, 0.2, and 0.5 m/s), resulting in **72 trials in total**. We now report the

mean and standard deviation of the peak absolute yaw deviation, together with the crash rate, in the revised Fig. 2.

Across all speeds, the repeated trials show a clear and consistent trend. The 15° configuration had the largest mean peak absolute yaw deviation (25.85° ± 5.19°) and the highest crash rate (75.0%). The 0° configuration also showed poor repeatability, with a mean peak yaw deviation of 17.73° ± 8.34° and a crash rate of 33.3%. By contrast, the 30°, 45°, and 60° configurations all achieved a 0% crash rate, with mean peak yaw deviations of 9.41° ± 2.76°, 5.78° ± 1.67°, and 7.22° ± 2.08°, respectively. Thus, among the crash-free positive-angle configurations, the 45° placement consistently showed the smallest mean peak yaw deviation.

These repeated trials provide a substantially stronger basis for assessing the reliability of the angle-placement study and for selecting the 45° configuration for the final design.

**Revision in the paper:**
The corresponding revisions have been described in full in our responses to Comments 1, 2, 3, and 6. To avoid repetition, we do not reproduce the full revised passage here.

## Comment 4

**Reviewer comment:**
The videos were not playable with QuickTime, only VLC works.

**Response:**
Thank you for reporting this issue. We have re-encoded all supplementary videos using a more widely supported H.264 format to improve compatibility with standard media players, including QuickTime.

## Comment 5

**Reviewer comment:**
How does it deal with the heavy oscillations that can be seen in the video, after sliding off the obstacle?

**Response:**
Thank you for pointing out the oscillations observed in the video after the whisker slides off the obstacle. These oscillations are primarily caused by whisker rebound and transient drone vibrations, which introduce high-frequency ringing in the barometric signals. To mitigate this effect, our tactile signal processing pipeline incorporates a bespoke drift compensation module (TDORC), which includes a band-pass filter to attenuate high-frequency components associated with whisker rebound and drone-induced vibrations. This step stabilizes the whisker signals **before** they are used for contact detection and distance estimation.

To clarify this behavior, we now provide additional plots showing the raw tactile signals, the

TDORC-processed outputs, the contact threshold, and the corresponding depth estimates for both left and right whiskers (Fig. S7). As highlighted in the shaded regions, the raw signals exhibit pronounced oscillations after sliding off the obstacle. In contrast, the TDORC-processed signals remain significantly more stable and suitable for reliable contact detection.

Importantly, depth estimation is only activated once contact is detected, i.e., when the processed signal exceeds a predefined threshold. As a result, transient oscillations occurring outside the contact regime do not affect the estimation process. We also observe that the processed signals may exhibit small negative values, particularly immediately after contact is lost. This is due to the transient response of the band-pass filter: when the signal undergoes a sudden drop from contact to no-contact, the filter produces a short negative undershoot. This behavior is expected and does not affect subsequent processing, because contact detection relies on a positive threshold applied to the processed signal.

**Revision in the paper:**
Page 13, Results, lines 261-266:
In addition, we evaluate the robustness of the proposed tactile processing pipeline under post-contact oscillations, which occur when the whisker slides off an obstacle. These oscillations are caused by whisker rebound and transient drone vibrations, and introduce high-frequency disturbances in the raw tactile signals. The proposed TDORC module effectively suppresses these oscillations before they are used for contact detection and depth estimation. Detailed results and analysis are provided in the Supplementary Material (Fig. S7).

Page S11 and S16, Supplementary Text, lines 954-976 and Figure S7:

## Robustness to Post-Contact Oscillations

During tactile flight, when the whisker slides off an obstacle, the system may experience transient oscillations caused by whisker rebound and drone-induced vibrations. These effects introduce high-frequency ringing in the raw (whisker) pressure signals. To evaluate the robustness of the proposed method under such conditions, we analyze representative segments of tactile navigation data, as shown in Fig. S7. For clarity of visualization, we show the top channel of each whisker, defined as the channel experiencing the largest deformation under contact and thus exhibiting the strongest response. This channel is also used for contact detection in our system. In practice, all channels are used in the proposed method, and similar behavior is consistently observed across them.

As highlighted in the shaded gray regions, the raw signals exhibit pronounced oscillations after sliding off the obstacle. In contrast, the TDORC-processed signals remain significantly more stable, effectively attenuating high-frequency components while preserving the informative tactile features required for contact detection. Depth estimation is only activated when the processed signal exceeds a predefined threshold. Therefore, oscillations occurring outside the contact regime do not influence the estimation process. This is reflected in Fig. S7, where the depth estimates remain stable despite

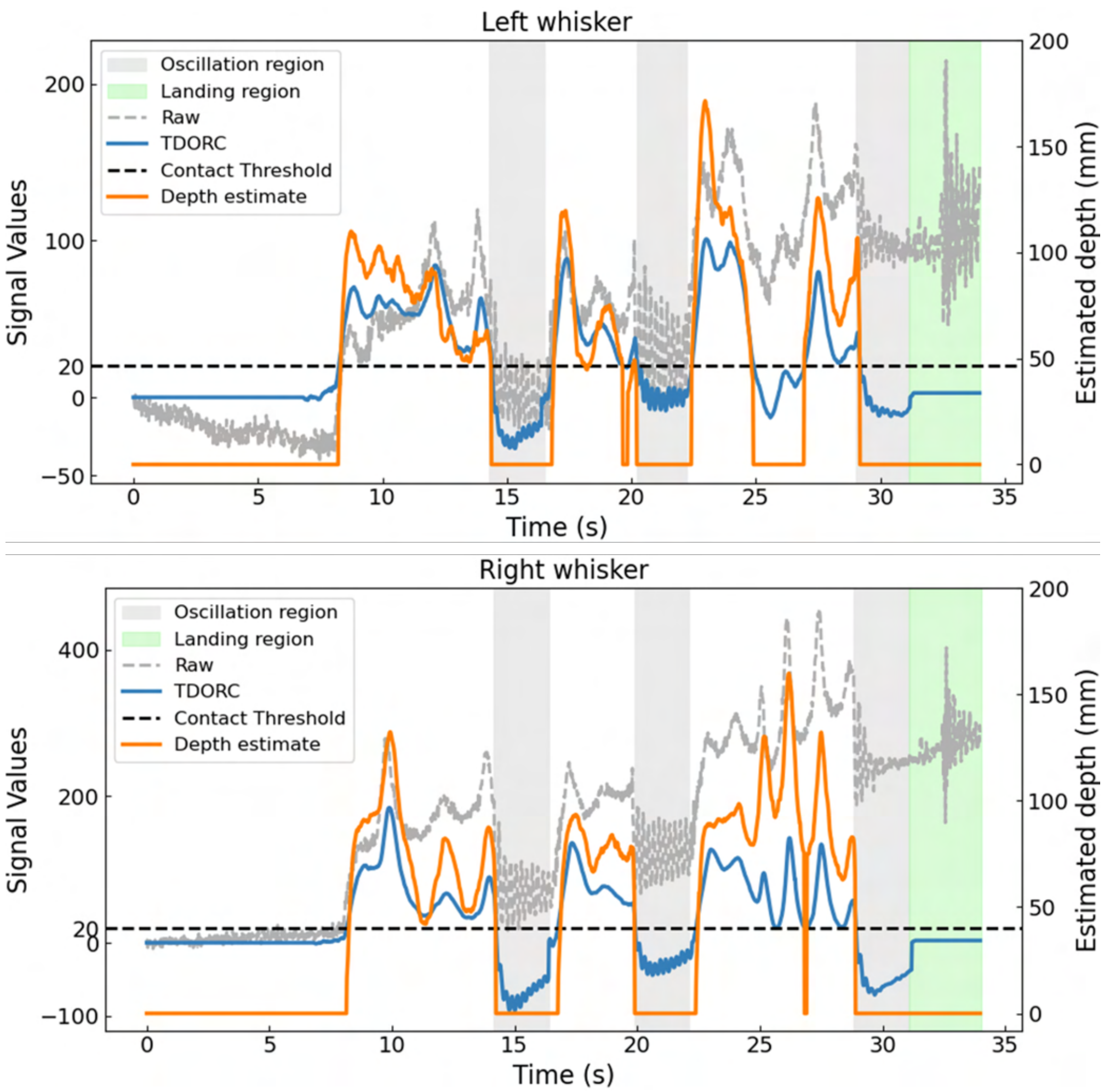


Figure S7: —Whisker signal processing and depth estimation under post-contact oscillations. Raw whisker signals (gray), TDORC-processed signals (blue), contact threshold (black dashed line), and corresponding depth estimates (orange) for the left (top) and right (bottom) whiskers. The shaded gray regions indicate intervals with strong post-contact oscillations caused by whisker rebound and drone vibrations after sliding off the obstacle. The green region denotes the landing phase. For clarity of visualization, we show the top channel of each whisker, defined as the channel experiencing the largest deformation under contact and thus exhibiting the strongest response. This channel is also used for contact detection in our system.

strong oscillations in the raw signals. The landing phase is also shown in the green region, where stable estimation is maintained after multiple sliding-off events.

We further observe that the processed signals may exhibit small negative values immediately after contact is lost. This is due to the transient response of the band-pass filtering stage: when the signal undergoes a sudden drop from contact to no-contact, the filter produces a short negative undershoot. This behavior is expected and does not affect subsequent processing. Since contact detection relies on a positive threshold applied to the processed signal, these transient values do not interfere with contact detection or the resulting depth estimation.

### Comment 8

**Reviewer comment:**
"F/T" not specified, I only realized on the third read that it is force/torque.

**Response:**
Thank you for pointing this out. We have now defined the acronym explicitly as Force/Torque (F/T) at its first occurrence in the text.

**Revision in the paper:**
Page 8, Results, lines 169–171:
While in principle PRBM could calculate $d$ from base moments, this requires calibrating the outputs of three barometers to contact forces using expensive high-precision Force/Torque (F/T) sensors, and can only be done while stationary—impractical in dynamic flight.

### Comment 9

**Reviewer comment:**
Crazyflie weight specifications in the text contradict Figure 1a in multiple places.

**Response:**
Thank you for the careful reading. We agree that the weight specifications in the original manuscript were not presented consistently. The value of 32 g corresponds to the official specification provided by Bitcraze for the Crazyflie 2.1 Brushless with a 350 mAh battery[2]. In our experiments, however, we also directly measured the mass of the drone and battery separately, obtaining 24.2 g for the drone and 9.2 g for the 350 mAh battery. These measured values correspond to the quantities shown in Fig. 1a and yield a total mass of 33.4 g for the bare platform and battery.

To avoid confusion, we revised the manuscript to report the measured values consistently where Fig. 1a is discussed.

**Revision in the paper:**
Page 21, Methods, lines 403 – 405:
The drone features 4 $\times$ 08028-10000KV high-quality motors and, with a measured weight of 24.2 g for the drone and 9.2 g for the 350 mAh battery (33.4 g total for the bare platform and battery),

achieves a flight endurance of over 10 minutes.

Page 22, Methods, lines 417 – 419:
In this configuration, the total weight was 44.1 g, and the platform achieved approximately 7 minutes of flight time when running our pipeline; details are provided in the Supplementary Materials, Flight Time Analysis.

### Comment 10

**Reviewer comment:**
"An identical protector is mounted on the drone's tail to provide symmetry and stability, with the battery slightly shifted backward to balance the center of mass." contradicts that the "minimizing the need for rear counterweight."

**Response:**
Thank you for the careful reading. We agree that the original wording was unclear. The battery is shifted slightly backward to partially compensate for the forward center-of-mass shift introduced by the whiskers, which improves stability during takeoff and general flight. However, this adjustment does not fully cancel the forward offset of the drone's center of mass. Instead, a small forward bias remains, and this remaining bias is partly compensated by the reaction moment generated during wall contact. We revised the manuscript to clarify both the mechanical rationale and the hardware implementation.

**Revision in the paper:**
Page 5, Results, lines 120–123:
To evaluate the effect of whisker placement, the drone performed controlled sweeping motions along a wall (Movie S1). We categorized placement angles in the pitch planes into three types: negative angles, 0°, and positive angles. The 0° placement was immediately ruled out due to whisker buckling and irregular signal outputs. Introducing a slight yaw angle in this configuration to maintain wall contact results in a normal force $N$, bending the whisker in the yaw plane, and a friction force $f_N$ during sweeping; together they induce a high yaw moment $M_{\text{yaw}}$, thereby compromising stability during wall sweeping. Negative and positive angles reduce yaw disturbance, as $f_N \ll N$, while the normal force primarily generates a pitch-plane moment $M_N$, which is easier to compensate, Fig. 2a and b. Between the two viable configurations, a positive angle is preferable. Forward-mounted whiskers generate a counterclockwise pitching moment $M_w$ due to the forward shift of the center of mass. During contact, the normal force $N$ produces a clockwise moment $M_N$ that partially offsets $M_w$, thereby reducing the overall pitching moment $M_{\text{pitch}}$ during contact interaction and reducing the need for additional rear counterweight. In contrast, negative angles reinforce $M_w$, increasing $M_{\text{pitch}}$ and destabilizing the drone. Pseudo Rigid-Body Modeling (PRBM) shows that as the placement angle increases, the normal force $N$ decreases for the same contact depth $d$ at a static point $P_c$, reducing friction force $f_N \propto N$. From this, we derived a physical model relating $N$ and $d$ (Methods). This analysis suggests that positive whisker angles should improve interaction stability, although excessively large angles also reduce the effective forward sensing range.

Page 21 and 22, Methods, lines 409 – 412:

An identical mounting was added on the drone's tail to provide symmetry and stability, with the battery position adjusted to partially compensate for the forward center-of-mass shift introduced by the whisker module, while maintaining a small forward bias that helps reduce clockwise pitch moment during contact interaction.

## Comment 11

**Reviewer comment:**

"To suppress hysteresis and ringing, we apply a first-order Butterworth bandpass filter (0.05–1 Hz), implemented as:" How can a band-pass filter suppress hysteresis?

**Response:**

Thank you for this insightful comment. We agree that the original wording in the Methods section was imprecise, as a band-pass filter does not suppress hysteresis in a general sense. We have revised the manuscript to clarify this point and to make the wording consistent with the earlier summary description of TDORC.

In the original manuscript, we wrote that the "Tactile Drift Online Recurrent Compensation (TDORC) algorithm addresses three noise sources in tactile sensing: temperature-induced signal drift, hysteresis from the gel's partial recovery, and vibration-induced ringing. That statement referred to the role of the overall TDORC pipeline." However, the later Methods description, which stated that a band-pass filter was used to "suppress hysteresis and ringing", was too compressed and could incorrectly suggest that the filter alone suppresses hysteresis in general.

In our setting, hysteresis-related effects appear in two forms. During sustained contact, the signal is affected by the intrinsic material response of the whisker and the sensor dynamics, which cannot be directly removed by filtering. In our system, this effect is instead partially mitigated through fusion with the process model via the Kalman filter, because the process model does not exhibit the same lag as the tactile signal. This behavior is consistent with the improved estimation and reconstruction results shown in Fig. 3, Table 1, and Fig. S6.

After contact is lost, an additional effect appears: the signal shows a delayed recovery toward baseline due to material response and sensor dynamics, resulting in a low-frequency residual that can interfere with subsequent contact detection. The proposed TDORC pipeline is designed to mitigate this post-contact recovery effect rather than hysteresis in general. Specifically, it combines online drift compensation with a first-order Butterworth band-pass filter (0.05–1 Hz). The high-pass component attenuates the low-frequency recovery after contact release, allowing the signal to return to baseline more rapidly, while the low-pass component suppresses high-frequency ringing caused by whisker rebound and drone-induced vibrations.

To make this distinction clearer, we added an additional comparison in Fig. S8. In the hysteresis-related recovery region, a low-pass-only filtered signal remains offset because of the slow recovery after contact release, whereas the proposed method restores the baseline more effectively. We have

therefore revised the manuscript so that the text attributes these effects to the overall TDORC pipeline, while stating more precisely that the band-pass filter itself mitigates delayed recovery after contact loss and high-frequency ringing, rather than hysteresis in general.

**Revision in the paper:**

Page 3, Introduction, lines 66–60:

To enable this, we develop and optimize lightweight whisker tactile sensors for tiny aerial robots, using force-aware structural design to minimize destabilizing torques. Complementing the hardware, we introduce a novel tactile signal processing pipeline that includes a Tactile Drift Online Recurrent Compensation (TDORC) algorithm, which corrects for drift caused by convective airflow, suppresses sensor ringing, and mitigates hysteretic post-contact recovery in the signal.

Page 8, Results, lines 147–153:

We propose the TDORC algorithm to address three noise sources in tactile sensing: temperature-induced signal drift, post-contact hysteretic recovery, and vibration-induced ringing (Extended Data Fig. 1b, yellow box). TDORC estimates temperature drift via first-order least squares within a sliding window ($n = 100$), updating it continuously during free flight—unlike Tactile Drift Online Compensation (TDOC), which calibrates only once at takeoff. The estimated drift is subtracted from the raw data, followed by band-pass filtering to suppress high-frequency ringing and mitigate delayed recovery toward baseline after contact loss.

Page 22, Methods, lines 428–431:

We propose the TDORC method to separate contact-related tactile signals from measurement artifacts. The raw signal $S_r(t)$ is modeled as the sum of the desired tactile response and three major artifact components: temperature-induced drift, hysteresis-related post-contact recovery effects, and vibration-induced ringing:

$$S_r(t) = S_t(t) + N_{\mathrm{td}}(t) + N_{\mathrm{h}}(t) + N_{\mathrm{r}}(t) \tag{1}$$

where $S_t(t)$ is true signal, $N_{\mathrm{td}}(t)$ is noise caused by temperature fluctuations,

Page 23, Methods, lines 448–449:

To mitigate delayed recovery from hysteresis after contact loss and to suppress high-frequency ringing, we apply a first-order Butterworth bandpass filter (0.05–1 Hz),

Page S16–S17, supplementary Text, lines 977–997 and Figure S8:

### Mitigation of Post-Contact Recovery Delay

During aerial tactile navigation, the whisker signals are affected not only by high-frequency oscillations but also by slow recovery dynamics after contact is lost. This effect arises from the partial deformation and recovery of the soft gel material, leading to a delayed return of the signal to its baseline. Such behavior can introduce a residual bias that interferes with subsequent contact detec-

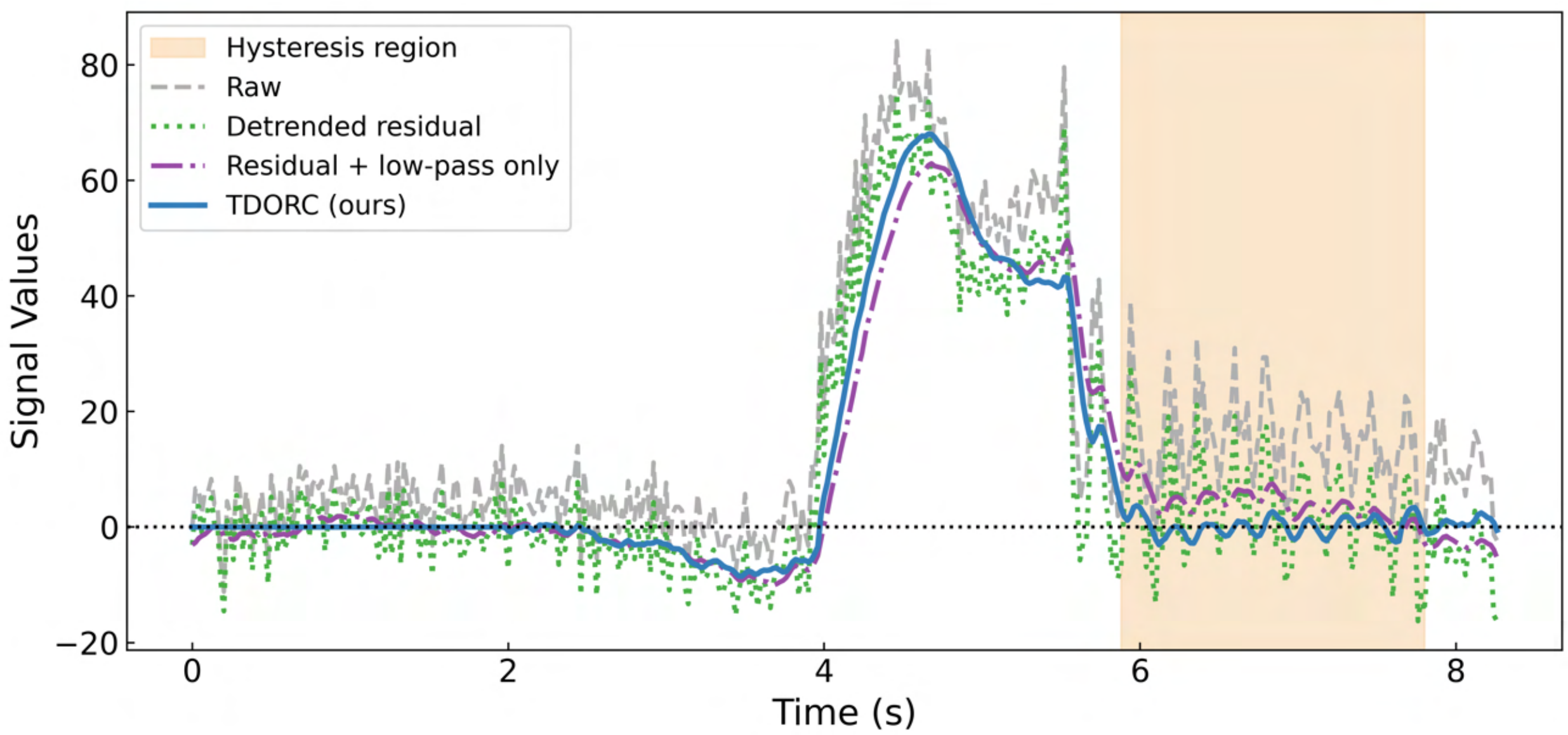


Figure S8: Comparison of the raw whisker signal (gray), detrended residual (green), residual with low-pass filtering only (purple), and the TDORC-processed signal (blue). The data are taken from the aerial tactile navigation experiment (Trial 4) when the drone passes the first wall. We show the top channel of the left whisker, i.e., the channel experiencing the largest deformation under contact and exhibiting the strongest response. The shaded region highlights the hysteresis-dominated interval after contact release, where the signal exhibits delayed recovery due to the soft material response. The low-pass-only signal remains biased and does not return to baseline, whereas TDORC effectively restores the signal to zero by attenuating low-frequency components associated with the recovery process. This behavior is critical for reliable contact detection.

tion. To analyze this effect, we consider data from the aerial tactile navigation experiment (Fig. 4, Trial 4), specifically when the drone passes the first wall. Fig. S8 shows the signal from the top channel of the left whisker, which corresponds to the channel undergoing the largest deformation and thus providing the most prominent contact response. Similar behavior is observed across other channels. As shown in Fig. S8, the raw signal exhibits both noise and a clear delay in returning to zero after contact release. Removing the linear trend (detrended residual) reduces drift but does not resolve the slow recovery behavior. Applying low-pass filtering alone further smooths the signal but retains the low-frequency bias, resulting in a signal that remains offset during the hysteresis-dominated interval. In contrast, the proposed TDORC method effectively mitigates this delayed recovery. By combining online drift compensation with a band-pass filter, the method attenuates both high-frequency ringing and low-frequency recovery components. As a result, the TDORC-processed signal returns to baseline more rapidly after contact loss, reducing the impact of recovery-induced bias. It is important to note that this approach does not remove the intrinsic hysteresis of the sensing material during contact. Instead, it mitigates its effect on the signal during the transition from contact to free space. This is particularly important for reliable contact detection in aerial tactile navigation, where rapid transitions between contact and free flight frequently occur.

## Comment 12

**Reviewer comment:**
Neural network seems overkill for a 3D ⇒ 1D regression. Linear regression should at least be provided as a baseline.

**Response:**
Thank you for this helpful suggestion. We agree that a simple linear regression baseline is important for evaluating whether a neural network is warranted for this 3D-to-1D regression task. In our study, this baseline was already included in the supplementary analysis under the polynomial fitting model. Specifically, the polynomial degree was selected on the training set using 5-fold cross-validation over degrees 1–10, and the optimal degree was consistently found to be 1 for both the left and right whisker models. Therefore, this model is equivalent to standard linear regression using the three barometric sensor channels as input.

We apologize for not making this point explicit in the previous version of the manuscript. To improve clarity, we revised the manuscript and Supplementary Materials to explicitly state that the polynomial baseline reduces to linear regression in this case, and we added Table S2 to summarize the hyperparameter search spaces and selected values for all compared sensor models. This revision makes it clearer that a linear regression baseline was indeed included, and that the MLP still consistently outperforms this linear baseline as well as the other tested methods.

**Revision in the paper:**
Page 9, Results, lines 184–190:
We compare our proposed method with depth estimation achieved using the sensor model alone,

based on variants of the two versions of the process models and the MLP-based sensor model. Among the sensor models, we present only the MLP in the main text, as it outperforms the other tested baseline sensor models on Dataset 1. Detailed quantitative comparisons for all sensor models are provided in the Supplementary Materials, including evaluation metrics across trials (Table S1), hyperparameter search spaces and selected values (Table. S2), and additional prediction, deviation, and reconstruction results (Fig.S6).

Table S2: Hyperparameter search spaces and selected values for all compared sensor models. For all methods, hyperparameters were selected on the training set using 5-fold cross-validation.

| **Model** | **Hyperparameter** | **Search range** | **Selected value** | |
|---|---|---|---|---|
| | | | **Left whisker** | **Right whisker** |
| PolyFit | Polynomial degree | $\{1, 2, \dots, 10\}$ | 1 | 1 |
| RFR | Number of trees | $\{100, 200, 300\}$ | 300 | 100 |
| | Maximum tree depth | $\{\text{None}, 5, 10, 20\}$ | 5 | 5 |
| | Minimum samples for split | $\{2, 5, 10\}$ | 5 | 10 |
| | Minimum samples per leaf | $\{1, 2, 4\}$ | 2 | 4 |
| GPR | Kernel amplitude $c$ | $\{1, 2, \dots, 10\}$ | 6 | 9 |
| | RBF length scale $l$ | $\{50, 100, \dots, 10000\}$ | 2450 | 4600 |
| MLP | Hidden units per layer | $\{16, 32\}$ | $(32, 32, 32)$ | $(32, 32, 32)$ |
| | Batch size | $\{32, 64, 128\}$ | 32 | 32 |
| | Learning rate | $\{10^{-4}, 10^{-3}\}$ | $10^{-3}$ | $10^{-3}$ |
| | Dropout | $\{0, 0.1, 0.5\}$ | 0.1 | 0.1 |
| | Regularization | $\{0, 10^{-4}, 10^{-3}, 10^{-2}\}$ | $10^{-3}$ | $10^{-2}$ |

Page S10, Supplementary Text, lines 930–933 and Table S2:
Table S1 presents the results of whisker-based tactile depth estimation using different models on Dataset 1. For all compared sensor models, hyperparameters were selected on the training set using 5-fold cross-validation. Table S2 summarizes the hyperparameter search spaces and the selected values for all compared models.

## Comment 13

**Reviewer comment:**
In my opinion, reiterating basics about neural networks or the Kalman filter do not add to the contribution of this work. Naturally, they have to be severely limited in scope.

**Response:**
Thank you for this suggestion. We agree that detailed explanations of these standard techniques should have been kept concise.

In the revised manuscript, we substantially shortened these sections and retained only the elements needed to understand our implementation: the whisker input representation, the state definition, the key assumptions of the motion model, and the final prediction and update equations used in the Kalman filter. More general implementation details, such as network architecture and hyperparameter selection, are now referred to the Supplementary Materials.

**Revision in the paper:**
Page 24–26, Methods, Page 456–490:

### Whisker sensor model

The whisker sensor model maps the pre-processed signals from the three barometers to a 1D contact depth estimate. For each whisker, the input to the model is the normalized three-channel signal vector $\mathbf{s}_\mathrm{n} = [S_\mathrm{n}^1, S_\mathrm{n}^2, S_\mathrm{n}^3]^\top$, where

$$S_\mathrm{n}^i = \frac{S_\mathrm{p}^i - \mu^i}{\sigma^i}, \quad i = 1, 2, 3, \tag{12}$$

and $\mu^i$ and $\sigma^i$ are the mean and standard deviation of the training data for the $i$-th barometer.

We use an MLP to predict depth from $\mathbf{s}_\mathrm{n}$. Ground-truth depths are obtained from the relative pose between the drone and the wall measured by the OptiTrack system. Since the platform operates in 2D, the left and right whisker depths are computed geometrically from the relative position and orientation of the drone and the wall. The network is trained by minimizing the mean squared error

$$\mathcal{L}_\mathrm{MSE} = \frac{1}{N}\sum_{i=1}^{N}(m_i - d_{t,i})^2, \tag{13}$$

where $m_i$ is the predicted depth and $d_{t,i}$ is the corresponding ground-truth depth for sample $i$. Optimization is performed using Adam. Network architecture and hyperparameter selection are provided in the Supplementary Materials.

### Whiskered drone motion model

To estimate wall distance from tactile sensing, we assume: (1) the contacted obstacle is static in the world frame, (2) each whisker has at most one contact point at a time, and (3) the contacted surface is locally planar.

As shown in Fig. S4, we define the state of the system in the drone frame $\{D\}$ as:

$$\mathbf{x}_k = \begin{bmatrix} d_{\mathrm{wall},k} \\ \theta_{\mathrm{wall},k} \end{bmatrix}, \tag{14}$$

where $d_{\mathrm{wall},k}$ is the perpendicular distance to the wall and $\theta_{\mathrm{wall},k}$ is the wall orientation relative to

the drone heading.

The wall orientation is estimated from the left and right whisker depths as

$$\theta^D_{\text{wall}} = \arctan\left(\frac{d_l - d_r}{s}\right), \tag{15}$$

where $d_l$ and $d_r$ are the estimated depths from the left and right whiskers, and $s$ is the distance between the whiskers. The relative wall orientation in the drone frame is related to the world-frame quantities by

$$\theta^D_{\text{wall}} = \theta^W_{\text{wall}} - \psi^W. \tag{16}$$

where $\psi^W$ is the drone yaw angle in the world frame. The drone displacement between two consecutive time steps is

$$\Delta p_k = \sqrt{(x_{d,k} - x_{d,k-1})^2 + (y_{d,k} - y_{d,k-1})^2}, \tag{17}$$

and the predicted wall distance is updated as

$$d^-_{k+1} = \frac{\Delta p_k \sin\big(\theta^W_{\text{wall},k} - \arctan(\Delta y/\Delta x)\big) + d_k \cos(\theta^D_{\text{wall},k})}{\cos(\theta^D_{\text{wall},k+1})}, \tag{18}$$

where $\Delta x = x_{d,k} - x_{d,k-1}$ and $\Delta y = y_{d,k} - y_{d,k-1}$.

For the simplified process model, we assume $d_l = d_r$, such that $\theta^D_{\text{wall}} = 0$.

## Sensor fusion for tactile-based depth estimation

We fuse the whisker-based depth estimate from the sensor model with the motion-model prediction using a Kalman filter. The prediction step uses Eq. (18), and the covariance is propagated as

$$\mathbf{P}^-_{k+1} = \mathbf{P}_k + \mathbf{Q}, \tag{19}$$

where $\mathbf{Q}$ is the process noise covariance.

The update step incorporates the sensor-model depth estimate $m$:

$$\mathbf{K}_k = \mathbf{P}^-_{k+1}\left(\mathbf{P}^-_{k+1} + \mathbf{R}\right)^{-1}, \tag{20}$$

$$d_{k+1} = d^-_{k+1} + \mathbf{K}_k(m - d^-_{k+1}), \tag{21}$$

$$\mathbf{P}_{k+1} = (\mathbf{I} - \mathbf{K}_k)\mathbf{P}^-_{k+1}, \tag{22}$$

where $\mathbf{R}$ is the measurement noise covariance.

## Comment 14

**Reviewer comment:**
It is not clear if the data in Table 1 is purely the test set or also contains training samples.

**Response:**
Thank you for pointing this out. The results reported in Table 1 are computed solely on the test set, which is separated from the training data during model development. No training samples are included in these evaluations. We agree that this was not clearly stated in the original manuscript, and we have revised the manuscript to make the test set explicit in both the table caption and the corresponding figure description.

**Revision in the paper:**
Page 10 and 11, Results, Table 1 and Figure 3 captions:

Table 1: **Evaluation metrics for different models across two datasets: Dataset 1 (whiteboard) and Dataset 2 (glass wall).** The table reports MAE and RMSE for each model across different estimation categories evaluated on the test set.

**Dataset 1 — Collected by flying close to a whiteboard**

| Model | **Whisker Left** (mm) | | **Whisker Right** (mm) | | **Orientation** (°) | | **Reconstruction** (mm) | |
|---|---|---|---|---|---|---|---|---|
| | MAE | RMSE | MAE | RMSE | MAE | RMSE | MAE | RMSE |
| **MLP** | 6.40 | 9.07 | 5.36 | 8.08 | 6.18 | 7.72 | 5.34 | 8.06 |
| **MLP + KF (Simplified)** | 6.19 | 8.35 | 5.13 | 7.09 | 5.96 | 7.36 | 4.73 | 6.99 |
| **MLP + KF (Full Model)** | **4.23** | **5.69** | **4.72** | **6.75** | **5.55** | **6.84** | **4.12** | **5.75** |

**Dataset 2 — Collected by flying close to a glass wall**

| Model | **Whisker Left** (mm) | | **Whisker Right** (mm) | | **Orientation** (°) | | **Reconstruction** (mm) | |
|---|---|---|---|---|---|---|---|---|
| | MAE | RMSE | MAE | RMSE | MAE | RMSE | MAE | RMSE |
| **MLP** | 11.33 | 14.69 | 5.78 | 8.15 | 11.41 | 14.79 | 8.13 | 9.87 |
| **MLP + KF (Simplified)** | 10.09 | 13.27 | **5.11** | **6.24** | 10.01 | 13.44 | 8.49 | 10.25 |
| **MLP + KF (Full Model)** | **5.91** | **7.68** | 5.38 | 7.03 | **5.06** | **6.60** | **5.71** | **7.06** |

## Comment 15

**Reviewer comment:**
In the exploration scenario, the algorithm seems to heavily rely on the optical flow sensor (which works very well out of the box and has low drift, in my experience). So it is not really clear how these experiments contribute to this work.

**Response:**
Thank you for this comment. We agree that the optical flow sensor provides reliable low-drift egomotion estimation for aerial platforms, and in our system it is used primarily for stabilization

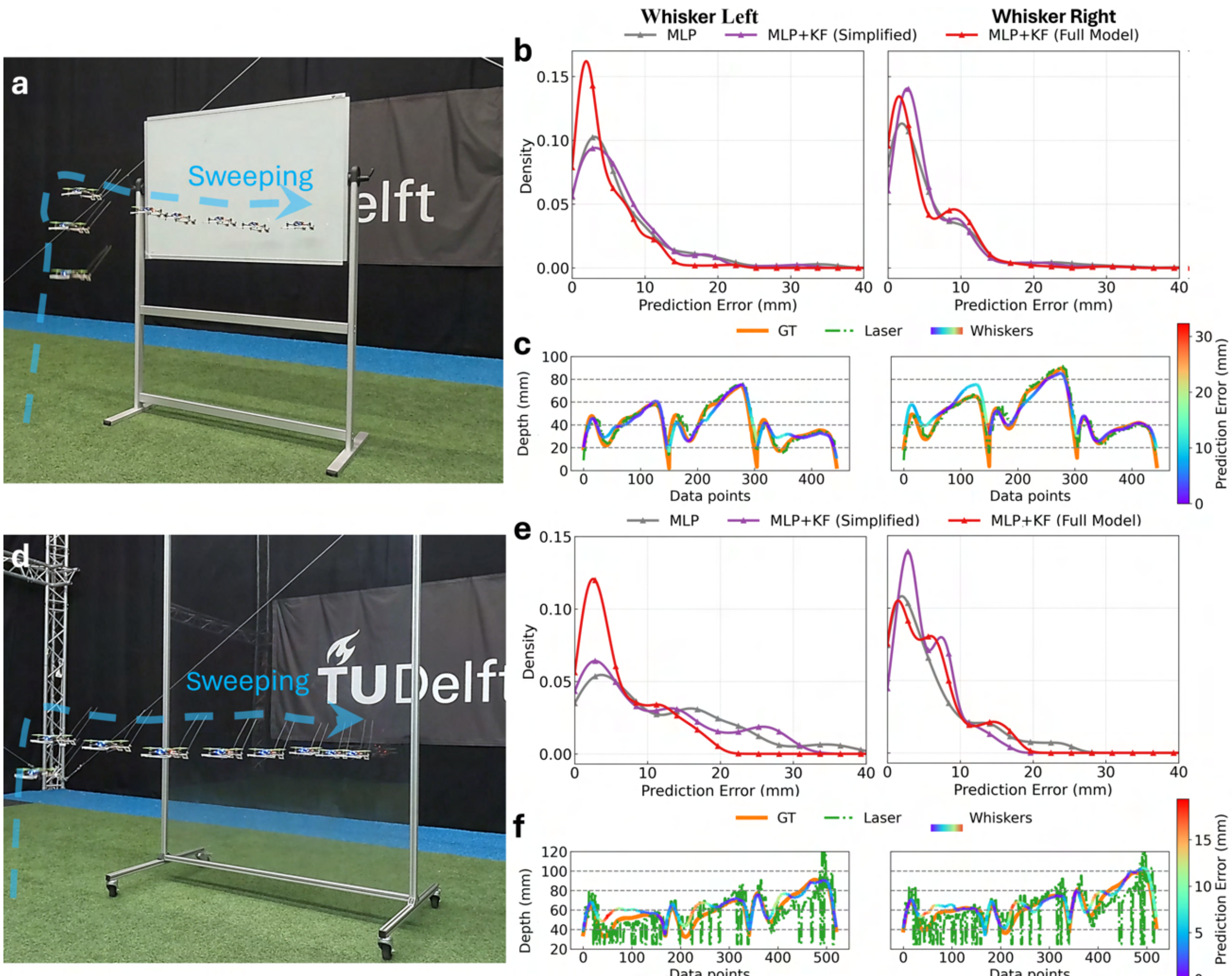


Figure 3: Whisker-based tactile-based depth estimation evaluated on the test set. (a) Data collection on a rigid panel (Dataset 1). (b) Prediction error density across models for Dataset 1. (c) Predicted depth from MLP+KF (Full Model) vs. GT and laser on Dataset 1. (d) Data collection on a rigid and transparent panel (Dataset 2). (e) Prediction error density across models for Dataset 2. (f) Predicted depth from MLP+KF (Full Model) vs. GT and laser on Dataset 2.

and motion estimation.

However, its role in our experiments is fundamentally different from that of the tactile sensing system. In our setup, the optical flow sensor is downward-facing and is used only for egomotion estimation relative to the floor, rather than for forward environmental perception. Optical flow provides only relative motion information and does not directly perceive the surrounding environment, such as obstacle presence, surface geometry, or proximity in visually degraded conditions. In particular, downward-looking optical flow measurements cannot support functions such as wall detection, surface contour following, or tactile mapping.

The contribution of our work lies in enabling these capabilities through whisker-based tactile sensing. The whiskers provide direct physical interaction with the environment, allowing the drone to infer distance, detect contact, and construct local environmental representations even in scenarios where vision-based or active ranging methods are unreliable or unavailable. In the exploration experiments, optical flow is used only for egomotion estimation and flight stabilization, whereas environment perception and navigation decisions are driven by tactile feedback. These experiments therefore evaluate not the odometry itself, but whether tactile sensing can support contact-driven mapping and navigation. In addition, all sensing, estimation, and control in our system are performed fully onboard the drone. The platform does not rely on external motion-capture systems or offboard computation to perform tactile exploration and navigation. We consider this an important aspect of the contribution, as it demonstrates that whisker-based tactile flight can be realized within the severe sensing, computation, and payload constraints of a tiny aerial robot. We have revised the manuscript to make this distinction more explicit.

**Revision in the paper:**
Page 18, Results, lines 333–336:
To validate our method in real-world scenarios, we constructed a 2m × 2m room with glass walls and an 80 cm-wide exit. As part of the experimental setup, optical flow is used only for egomotion estimation and flight stabilization, while all environment perception and navigation decisions are driven by tactile sensing onboard. As shown in Fig. 5g and h, the drone successfully explored the environment and located the exit.

## Comment 16

**Reviewer comment:**
The exploration in particular seems to be a stack of many heuristics/design decisions that are not properly ablated.

**Response:**
We thank the reviewer for this comment. We agree that the original manuscript did not sufficiently isolate the contributions of the individual components in the exploration strategy. To address this concern, we added a systematic ablation study that quantitatively evaluates the roles of the corner-penalty module, the sweeping behavior, and active exploration guidance.

Specifically, removing the corner-penalty module reduces the exit success rate from 90% to 50%, while increasing the average travel distance from $25.38 \pm 5.26$ m to $34.16 \pm 4.48$ m and increasing the sweeping count from $7.00 \pm 1.41$ to $8.60 \pm 0.89$. Removing the sweeping behavior further degrades performance, reducing the exit success rate to 40% and worsening reconstruction accuracy from $0.26 \pm 0.06$ m to $0.33 \pm 0.09$ m in Chamfer Distance and from $0.14 \pm 0.03$ m to $0.19 \pm 0.07$ m in RMSD, while also increasing the exploration cost. Replacing active exploration guidance with random exploration leads to the worst performance across all metrics, with only a 10% exit success rate.

To further clarify the role of each component, we also added a failure-case analysis and qualitative visualizations across all trials. The failure-case analysis shows that removing the corner-penalty module leads primarily to crashing failures, whereas removing sweeping leads more often to timeout failures caused by insufficient local coverage. Random exploration exhibits both frequent crashing and timeout events, reflecting the lack of an information-driven exploration strategy. The qualitative visualization show the drone trajectories, reconstructed contours, ground-truth layouts, and crashing events, and they further highlight the role of the corner-penalty mechanism in avoiding concave regions. In particular, without this component, the drone frequently collides with sharp corners, whereas the proposed method consistently steers away from such high-risk regions. They also show that removing the sweeping behavior leads to less accurate reconstructions because local geometric coverage is reduced.

These ablation results now clearly show the value of the proposed, complementary mechanisms. The corner-penalty term improves safety by reducing crashing events, sweeping improves local coverage and geometric reconstruction, and active exploration guidance improves exploration efficiency. The corresponding revisions are shown below.

**Revision in the paper:**

Page 15 – 18, Results, lines 293–332, Figure 5 and Table 2:

We conducted 10 simulation trials in randomly rotated room orientations. The proposed method successfully located the exit in 9 out of 10 trials (90% success rate) within 5 min, achieving a mean reconstruction accuracy of $0.26 \pm 0.06$ m (CD) and $0.14 \pm 0.03$ m (RMSD), with a mean travel distance of $25.38 \pm 5.26$ m.

To further evaluate the contribution of each component, we perform a systematic ablation study, as summarized in Table 2. The definitions of the baseline variants and evaluation metrics are provided in Methods. The full method consistently outperforms all ablated variants in both reconstruction accuracy and exploration efficiency. Removing the corner-penalty module significantly degrades performance, reducing the success rate to 50% and increasing both travel distance and sweeping count. Similarly, removing the sweeping behavior further deteriorates both mapping accuracy and efficiency. Random exploration performs the worst across all metrics, as it does not explicitly leverage the acquired observations to guide exploration. Failure cases are further analyzed in Fig. S10,

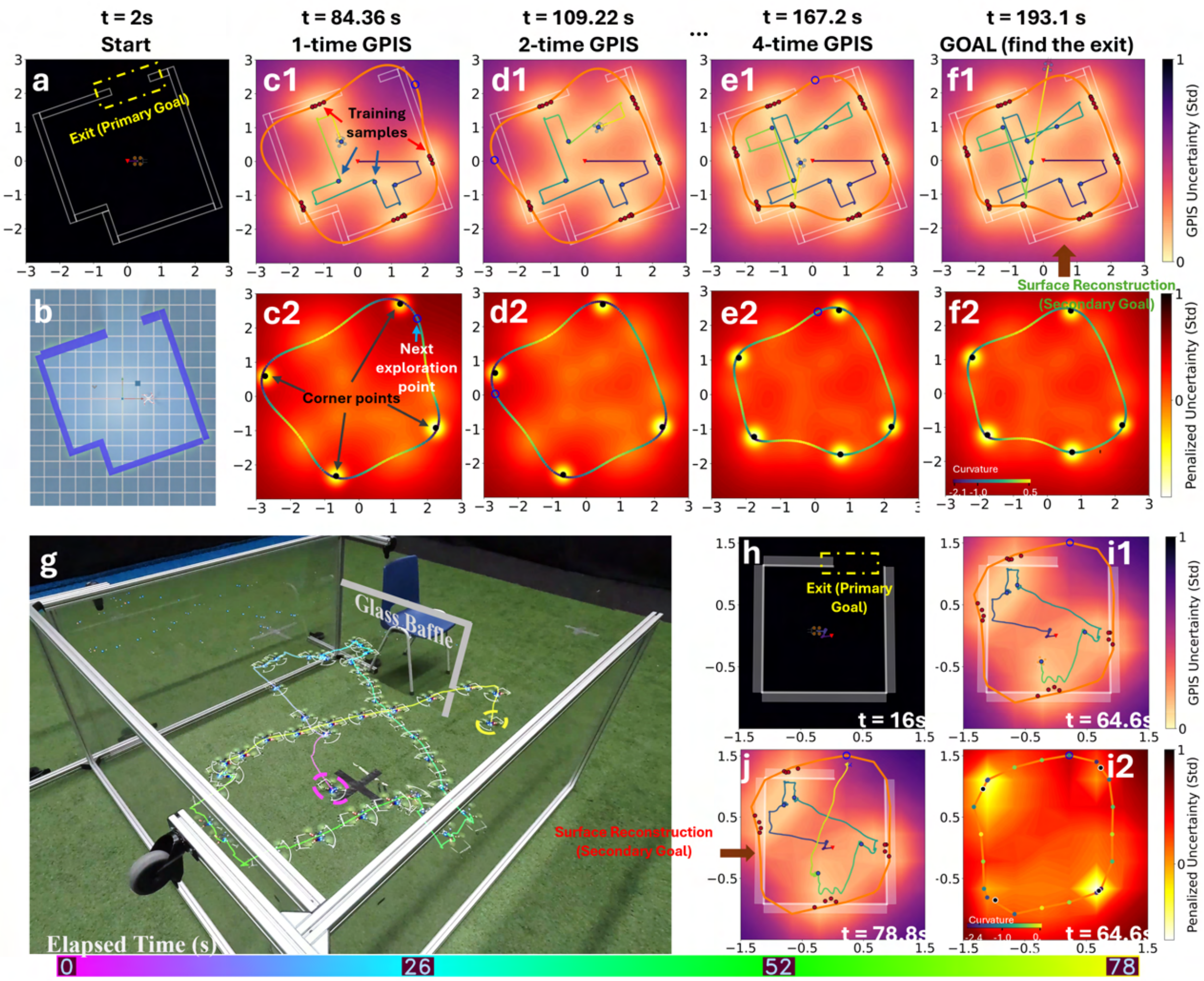


Figure 5: Simulation and real-world results of active aerial tactile exploration. (a) Initial unexplored environment with maximum uncertainty. (b) Simulation in ISAAC SIM. (c1, d1, e1, f1) Successive GPIS applications after collecting training data (red: surface; blue: interior), showing reduced uncertainty and improved shape reconstruction. The drone's trajectory (rainbow line), reconstructed contours (orange), and real-time state (drone icon) are shown. The exit is reached after the fourth GPIS. (c2, d2, e2, f2) Curvature analysis of extracted contours: brighter colors indicate higher curvature. Identified convex corners (white dots) are penalized, and the next exploration target is selected (blue hollow circle) based on updated uncertainty. (g) Real-world trajectory with time-color-coded path; start and landing points marked. (h) Initial environment map with maximum uncertainty. (i1) Initial exploration in four directions to gather GPIS data, with corresponding trajectory and reconstruction. (i2) Real-world curvature analysis guiding target selection. (j) Final reconstructed map with successful exit navigation; minor deviations are due to odometry drift.

Table 2: **Quantitative evaluation of active aerial tactile exploration.** The table reports exit success rate, reconstruction accuracy, and exploration efficiency for different methods, including ablation variants. Exit success is defined as successfully locating and navigating out of the environment within 5 min. Reconstruction accuracy is measured by Chamfer Distance (CD) and Root Mean Square Distance (RMSD). Exploration efficiency is measured by travel distance and sweeping count, and is computed only over trials that were not prematurely terminated by crashing events. Each method is evaluated over 10 trials with randomly rotated environment orientations. To ensure fair comparison, trials in which the drone exits the environment immediately without performing meaningful exploration are excluded.

| Method | **Exit Success** | **Reconstruction Accuracy** | | **Exploration Efficiency** | |
|---|---|---|---|---|---|
| | **SR (%)↑** | **CD (m)↓** | **RMSD (m)↓** | **Distance (m)↓** | **Sweeping Count↓** |
| **Ours (Full)** | **90** | **0.26 ± 0.06** | **0.14 ± 0.03** | **25.38 ± 5.26** | **7.00 ± 1.41** |
| w/o Corner Penalty | 50 | 0.27 ± 0.08 | 0.16 ± 0.06 | 34.16 ± 4.48 | 8.60 ± 0.89 |
| w/o Sweeping | 40 | 0.33 ± 0.09 | 0.19 ± 0.07 | 32.60 ± 10.88 | 10.11 ± 3.48 |
| Random Exploration | 10 | 0.36 ± 0.12 | 0.25 ± 0.13 | 33.86 ± 9.16 | 10.50 ± 3.00 |

which breaks down the outcomes into success, crashing, and timeout events. The results reveal distinct failure patterns across methods. Removing the corner-penalty module leads to a substantial increase in crashing events, which account for the majority of failures in this variant. In contrast, removing the sweeping behavior results in a higher proportion of timeout cases, suggesting inefficient exploration with limited environment coverage. Random exploration exhibits both frequent crashing and timeout events, reflecting the lack of an information-driven exploration strategy.

Additional qualitative results across all trials are provided in Supplementary Fig. S11, where we visualize the drone trajectories, reconstructed contours, GT layouts, and failure cases (indicated by red crosses for crashing events). These results further highlight the role of the corner-penalty mechanism in avoiding concave regions. Without this component, the drone frequently collides with sharp corners, as observed in both the w/o corner-penalty and random exploration variants. In contrast, the proposed method consistently steers away from such high-risk regions. We also observe that removing the sweeping behavior leads to noticeably less accurate reconstructions, as the lack of local coverage limits the acquisition of fine geometric details. Finally, we note a limitation of the current strategy: in some cases (e.g., Trial 8), the drone locates the exit before fully exploring the environment, resulting in incomplete reconstruction. This suggests that future work could incorporate an uncertainty-aware exploration strategy to prevent premature exit and enable the drone to return and continue exploring until sufficient environment coverage is achieved.

Fig. 5 illustrates one successful exploration, which panel Fig. 5c1 shows the first application of GPIS to the uncertainty map after collecting training data in four orthogonal orientations (red dots: surface; blue dots: interior). Compared to Fig. 5a, uncertainty is significantly reduced in explored regions. The drone's trajectory and current state are indicated by a small drone icon, with reconstructed contours in orange using the marching squares algorithm. Curvature analysis (Fig. 5c2) highlights high-curvature corners, which are penalized to guide the next exploration target (blue hollow circle). Subsequent GPIS applications and corner penalties (Fig. 5d1–f2) progressively refine the map and reduce uncertainty. For clarity, we omit the third GPIS application from the

figure. The drone successfully located the exit after the fourth GPIS application.

Page 31 – 32, Methods, lines 611–647

To facilitate quantitative evaluation, we implement several baseline and ablation variants of the proposed exploration strategy in simulation. Specifically, we consider: (1) *w/o Corner Penalty*, in which the curvature-based penalty is removed; (2) *w/o Sweeping*, in which the wall-following sweeping behavior is disabled; in this case, the corner-penalty term is also inactive, since corner avoidance is only needed during sweeping-based target selection; and (3) *Random Exploration*, in which the drone selects exploration directions without leveraging GPIS uncertainty or geometric cues. All methods share the same initialization procedure, where the drone first explores in four orthogonal directions to build a coarse environmental model.

We evaluate performance using three groups of metrics.

*Exit success rate* is defined as the percentage of trials in which the drone successfully locates the exit and flies out of the environment within 5 min.

*Reconstruction accuracy* is quantified using Chamfer Distance (CD) and Root Mean Square Distance (RMSD) between the reconstructed contour and the ground-truth contour.Let $\mathcal{P} = \{\mathbf{p}_i\}$ and $\mathcal{G} = \{\mathbf{g}_i\}$ denote the sampled points on the reconstructed and ground-truth contours, respectively. The Chamfer Distance is defined as

$$\mathrm{CD}(\mathcal{P}, \mathcal{G}) = \frac{1}{|\mathcal{P}|} \sum_{p \in \mathcal{P}} \min_{g \in \mathcal{G}} \|p - g\|_2 + \frac{1}{|\mathcal{G}|} \sum_{g \in \mathcal{G}} \min_{p \in \mathcal{P}} \|g - p\|_2, \tag{23}$$

where $|\mathcal{P}|$ and $|\mathcal{G}|$ are the numbers of sampled points in the reconstructed and ground-truth contours, respectively, and $\|\cdot\|_2$ denotes the Euclidean norm. CD measures the bidirectional average nearest-neighbor distance between the two contours.

The RMSD is defined as

$$\mathrm{RMSD}(\mathcal{P}, \mathcal{G}) = \sqrt{\frac{1}{|\mathcal{P}|} \sum_{p \in \mathcal{P}} d(p, \mathcal{G})^2}, \tag{24}$$

where $d(p, \mathcal{G}) = \min_{g \in \mathcal{G}} \|p - g\|_2$. In our implementation, this distance is computed as the minimum point-to-segment distance from $p$ to the piecewise-linear ground-truth contour.

*Exploration efficiency* is measured by the total travel distance and the sweeping count. The travel distance is computed as the accumulated Euclidean distance along the drone trajectory. The sweeping count denotes the number of sweeping interactions performed during exploration. To ensure fair comparison, exploration-efficiency metrics are computed only over trials that are not prematurely terminated by crashing events, whereas reconstruction metrics are reported over all trials.

Page S20 – S21, Supplementary Text, lines 1033–1039, Figure S10 and Figure 11:

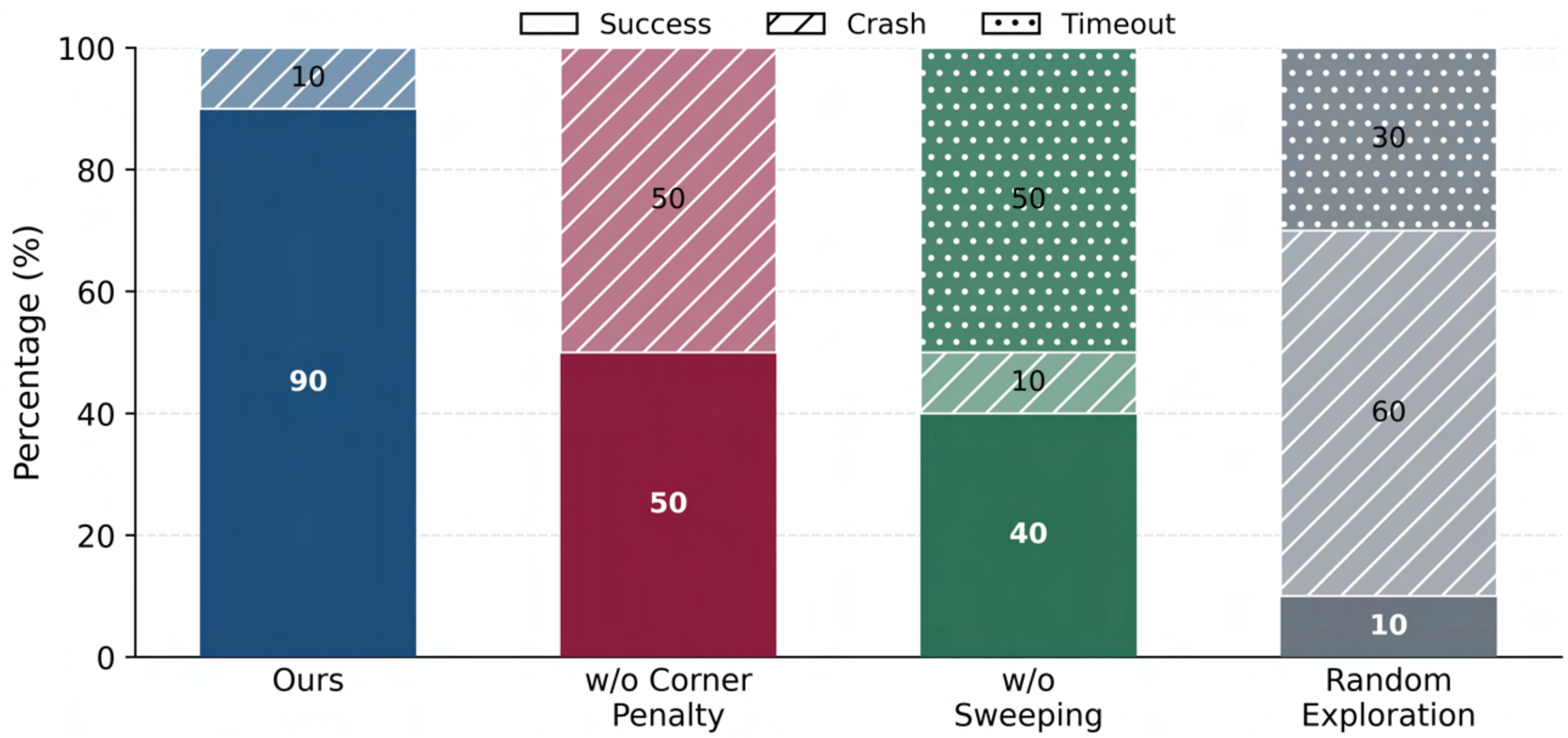


Figure S10: Failure modes across tactile exploration methods. Removing the corner-penalty module leads to a substantial increase in failures due to crashes. In contrast, removing the sweeping behaviour results in more timeout cases due to insufficient exploration. Random exploration exhibits both frequent crashes and timeout events.

### Ablation study on active aerial tactile exploration

Supplementary Fig. S10 shows the breakdown of trial outcomes into success, crashing, and timeout events across different methods.

Supplementary Fig. S11 presents qualitative results for all trials, including drone trajectories, reconstructed contours, GT layouts, and failure cases (red crosses indicate crashing events).

These results further corroborate the distinct roles of each component in shaping tactile flight exploration performance and failure modes.

## Comment 17

**Reviewer comment:**
The avoidance of concave regions is not clearly demonstrated.

**Response:**
We agree that the avoidance of concave regions should be demonstrated clearly. To address this point, we provide further quantitative and qualitative evidence in the revised manuscript.

Quantitatively, the newly added failure-mode analysis shows that removing the corner-penalty module leads to a sharp increase in crashing events, which become the dominant failure mode for that variant. A similar pattern is observed for the random-exploration baseline. These results are consistent with the drone entering high-curvature, concave regions without curvature-aware

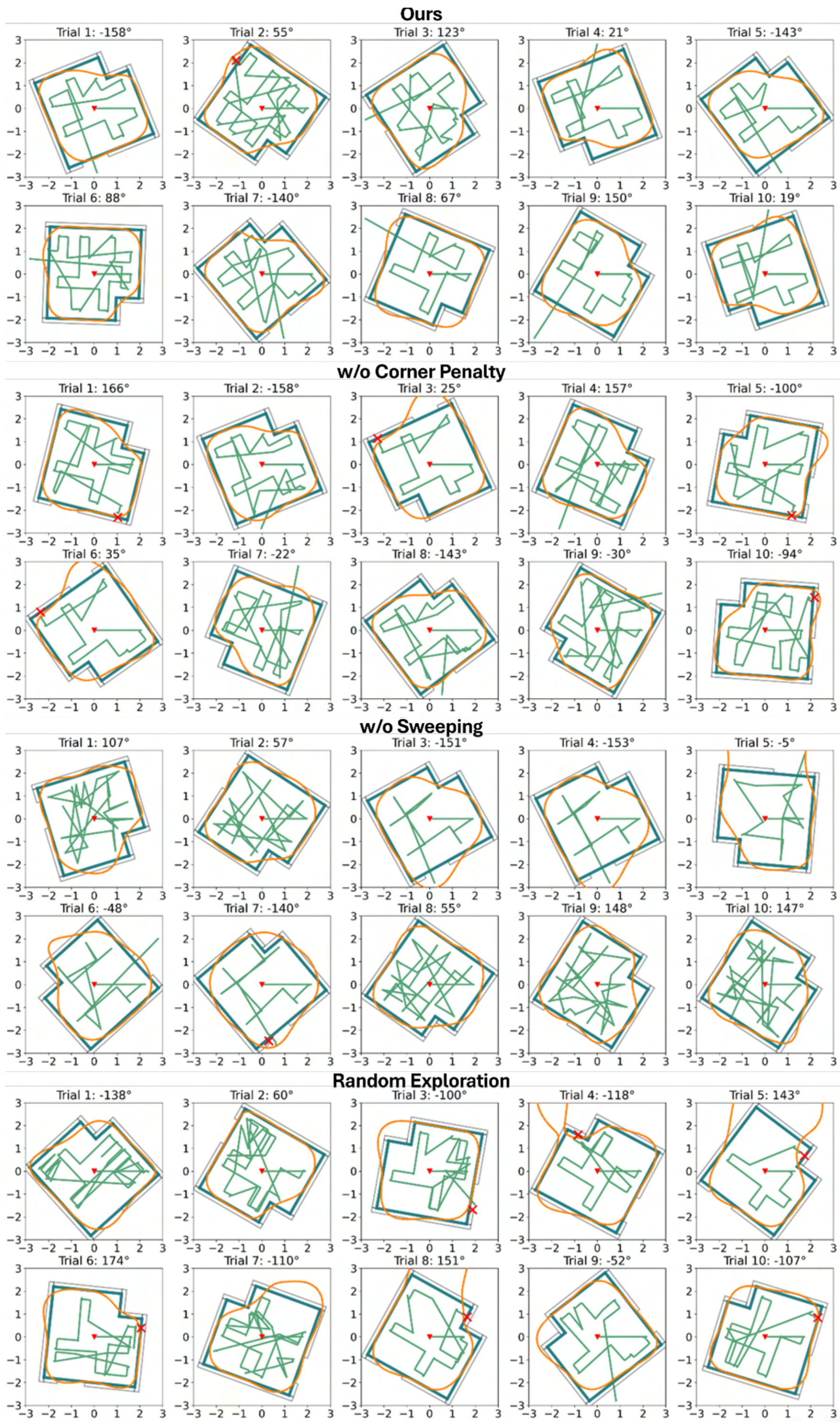


Figure S11: Qualitative results across all trials. Each subplot shows the drone trajectory (green), reconstructed contour (orange), and GT layout (blue). Crash cases are indicated by red crosses. Without the corner-penalty module, the drone frequently enters concave regions and collides with sharp corners. In contrast, the proposed method consistently avoids these regions and produces stable exploration trajectories.

guidance.

Qualitatively, Supplementary Fig. S11 now shows all trials, including drone trajectories, reconstructed contours, ground-truth layouts, and crashing events marked by red crosses. These visualizations show that, without the corner-penalty mechanism, the drone more frequently enters concave regions and collides with sharp corners, whereas the proposed method more consistently steers away from such high-risk regions.

Together, these additions provide clearer statistical and visual evidence that the curvature-based corner-penalty mechanism improves avoidance of concave geometries during tactile exploration.

**Revision in the paper:**
Same as in response to Comment 16.

### Conclusion

**Reviewer comment:**
This work proposes an interesting mechatronic and signal-processing method to add tactile sensing to aerial vehicles. The whisker-based design is shown to work in the real world, but many of its aspects and design decisions are not ablated or justified well. It is an interesting initial exploration for this type of sensor, but e.g. it is not clear to me why using barometric sensors is the best choice. Due to the novelty, there seems to be no baseline to compare it to, but it would still be valuable if the authors could elaborate more on what went into that design decision. Including what alternatives were considered and how it can be improved in the future.

**Response:**
We thank the reviewer for this thoughtful overall assessment. We agree that, in the original manuscript, some design choices were not sufficiently justified and that the motivation for selecting barometric sensing deserved a clearer explanation.

To address this, we have strengthened the revised manuscript in several ways. Firstly, we added new ablation and repeated-validation experiments, including the expanded whisker placement-angle study and the active exploration ablation analysis, to better isolate the contributions of the main design components. Secondly, we revised the Supplementary Materials to clarify the rationale for choosing barometric sensing under the specific constraints of aerial tactile exploration, including strict limits on payload, onboard space, onboard computation, and robustness under repeated contact. We now also discuss more explicitly two representative alternative sensing directions commonly explored in whisker sensing, namely vision-based and magnetic-based approaches, and explain why these were not adopted in the present aerial platform. Thirdly, we expanded the limitations and future-work discussion to more clearly state where the current system can be improved, including calibration scalability, curved-surface following, and more advanced tactile servoing and mapping strategies.

We agree that this work introduces the concept of whisker-based tactile flight, which can be expanded in several new research directions. We have revised the manuscript to make both the

current design rationale and its limitations more explicit.

**Revision in the paper:**
Page S3, Supplementary Text, lines 845 – 856:

### Choice of sensing modality

The choice of barometric sensing is driven by the constraints of aerial tactile exploration rather than by a direct benchmark against alternative modalities. Small aerial robots impose strict limits on payload, onboard computation, and robustness under repeated transitions between contact and free flight. Among the representative alternatives commonly explored in whisker sensing, vision-based whisker sensing typically requires additional imaging hardware and processing, increasing computational load and system footprint [7, 6]. Magnetic-based approaches often require extra magnets, holders, or alignment structures, which increase integration complexity and size [9, 8]. In addition, achieving high sensitivity with long, slender whiskers typically requires compliant deformation structures, introducing a trade-off with mechanical robustness [10]. In contrast, barometric sensing enables a compact, lightweight, and sensitive implementation that is well suited to aerial deployment [14].

## Code availability

**Reviewer comment:**
I have briefly inspected the codebase (I don't have a compatible machine to run it available right now). It seems like the code for the method described in this paper is not yet included. I found the dataset and the simulator used in this work. The repo states "Note: The full source code will be released upon acceptance of the article." so I assume the relevant code is not available.

**Response:**
We thank the reviewer for checking the repository and for pointing this out. At the time of submission, the repository only contained the dataset and simulator, and the full implementation of the method had not yet been made public. We have now updated the GitHub repository to include the code corresponding to the method described in this paper.

Specifically, the repository now contains both the simulation and real-world implementations. The simulation code is provided under `simulation/WhiskeredDroneSim/`, including the modified drone simulation framework for whisker-based exploration. The real-world implementation is provided under `real_world/`, including the modified Crazyflie firmware, whisker sensor integration, onboard tactile navigation and exploration. We have also added the code for whisker depth estimation, including classical baselines, MLP training/testing, and Kalman-filter-based post-processing using pretrained models.

The README has been revised accordingly to clarify the repository structure, dependencies,

and setup instructions. In particular, detailed instructions are provided in `Dataset/README.md`, `simulation/README.md`, and `real_world/README.md`. We have also updated the Code availability statement in the manuscript to reflect that the full source code is now included in the public repository.

**Revision in the paper:**
Page 32 and 33, lines 638 – 645:

## Data availability

All data supporting the findings of this study, including the simulation and real-world experimental data, are available in the Supplementary Materials. Source Data are provided with this paper.

## Code availability

The code used in this study is publicly available at `https://github.com/BioMorphic-Intelligence-Lab/Whiskered-drone`. The repository includes the simulation code, the modified Crazyflie firmware for the real-world platform, the onboard tactile navigation and exploration implementation, and the whisker depth estimation code.

## Reviewer #4

### Summary:

The paper presents a whisker-based tactile sensing system for tiny drones. The authors design a barometer-based whisker apparatus and integrate it on a Crazyflie platform. The system enables wall-following, navigation, and exploration using touch as the primary sensing modality, including operation in complete darkness. The paper presents clear novelty by enabling tactile flight on an ultra-light drone with fully onboard execution under strict memory and computation constraints. It demonstrates strong system integration, combining hardware design, drift compensation, learning-based depth estimation, filtering, control, and exploration into a coherent pipeline. The experimental validation is convincing and comprehensive. The work also has strong practical relevance, addressing failure modes of vision and laser sensing, such as darkness and transparency, with particularly compelling demonstrations in complete darkness. That said, the following points may help further strengthen the paper.

**Response:**
We thank Reviewer #4 for this positive and thoughtful overall assessment. We particularly appreciate the reviewer's recognition of the novelty of whisker-based tactile flight on a tiny drone, the

fully onboard implementation under strict memory and computation constraints, the system-level integration, and the practical relevance of tactile sensing in conditions where vision and laser-based sensing can fail. We are encouraged that the reviewer found the experimental validation convincing and comprehensive. Below, we address the reviewer's comments point by point.

## Comment 1

**Reviewer comment:**
While related work is cited, there is no direct experimental comparison with other aerial tactile approaches, such as compliant bumpers. It is therefore difficult to quantify the improvement in stability or perception quality. Can the authors provide a quantitative comparison between whisker sensing and collision-resilient bumper-based drones in terms of stability, impact force, and perception capabilities?

**Response:**
We thank the reviewer for this comment. We would like to clarify that a direct quantitative comparison between our whisker-based system and prior collision-resilient aerial systems is not straightforward, because these platforms differ substantially in mass, contact interface, sensing range, and the type of tactile/contact information they provide.

As summarized in Table S3, XPLORER and RMF also exploit continuous contact information. However, compared with the present work, their sensing range is much shorter, since contact information is obtained through body or flap deformation rather than through forward-reaching whiskers. In addition, these systems generally involve larger interaction forces, larger contact areas, larger structural footprint, and substantially heavier platforms. Notably, lighter prior systems at 133 g and 321 g provide only binary collision-event sensing, whereas the systems that exploit continuous contact information are substantially heavier, at 495 g and 1.12 kg, compared with 44.1 g in this work.

These differences lead to qualitatively different interaction mechanics for tactile wall following. In particular, our whisker-placement experiments show that even modest changes in contact-induced moment can strongly affect flight stability on a very small aerial platform. Hence, we expect that bumper-based systems with a large contact area will experience large, destabilizing contact-induced moments. In contrast, the proposed system provides localized continuous tactile sensing with a longer sensing range and very low interaction force, which are particularly well suited to active tactile sweeping and wall following on a tiny drone.

Among the compared systems, XPLORER is the closest in spirit in that it supports sustained contact and sweeping-like interaction, but it does so through body deformation and force estimation on a much heavier deformable platform. RMF, while equipped with continuous contact sensing, primarily relies on laser/ToF-based obstacle avoidance rather than tactile sensing as the principal navigation modality. These differences highlight that the compared systems are not directly equivalent benchmarks for the tactile sensing problem addressed in this work.

Table S3: **Interface-level comparison between whisker-based tactile sensing and representative collision-resilient drones equipped with mechanical bumpers.** The table highlights contact-interface properties relevant to tactile wall following, rather than providing a direct performance benchmark, since the platforms, tasks, and sensing goals differ substantially across studies.

| System | Weight | Tactile/contact information | Interaction force | Supports active tactile sweeping | Sensing range |
|---|---|---|---|---|---|
| **This work** | **44.1 g** | **Localized continuous tactile sensing** | **Very Low** | **Yes** | **Large (whisker deformation)** |
| RMF with laser avoidance [5] | 495 g | Flexion-based continuous contact sensing | Low | No | Moderate (flap deformation) |
| XPLORER [12] | 1.12 kg | Force-based continuous tactile sensing | High (body contact) | Yes | Very short (body deformation) |
| [4] | 321 g | Discrete collision event | High (body contact) | No | None |
| Tiercel [11] | 133 g | Discrete collision events | High (body contact) | No | None |
| Air Bumper [13] | 1.45 kg | Discrete collision events | High (body contact) | No | None |

We have revised the manuscript to make this distinction clearer and added Table S3 to summarize the interface-level differences.

**Revision in the paper:**
Page S25 and S26, Supplementary Text, lines 1080 – 1101 and Table S3:

**Comparison with collision-resilient aerial systems**

Table S3 summarizes key interface-level differences between the proposed whisker-based system and representative collision-resilient aerial platforms. XPLORER[12] and RMF[5] also exploit continuous contact information. However, compared with the present work, their sensing range is much shorter, since contact information is obtained through body or flap deformation rather than through forward-reaching whiskers. In addition, these systems generally involve larger interaction forces, larger contact areas, larger structural footprint, and substantially heavier platforms. No-

tably, lighter prior systems at 133 g and 321 g provide only binary collision-event sensing, whereas the systems that exploit continuous contact information are substantially heavier, at 495 g and 1.12 kg, compared with 44.1 g in this work. These differences lead to qualitatively different interaction mechanics for tactile wall following. In particular, our whisker-placement experiments already show that even modest changes in contact-induced moment can strongly affect flight stability on a very small aerial platform. Hence, we expect that bumper-based systems with a large contact area will experience large, destabilizing contact-induced moments. In contrast, the proposed system provides localized continuous tactile sensing with a longer sensing range and very low interaction force, which are particularly suited to active tactile sweeping and wall following on a tiny drone. Among the compared systems, XPLORER is the closest in spirit in that it supports sustained contact and sweeping-like interaction, but it does so through body deformation and force estimation on a much heavier deformable platform. RMF, while equipped with continuous contact sensing, primarily relies on laser/ToF-based obstacle avoidance rather than tactile sensing as the principal navigation modality. These differences highlight that the compared systems are not directly equivalent benchmarks for the tactile sensing problem addressed in this work.

### Comment 2

**Reviewer comment:**
What is the exact MLP architecture used for depth estimation? How many parameters does it contain?

**Response:**
Thank you for pointing this out. We apologize for not clearly specifying the network architecture in the original manuscript. The depth estimation model uses a lightweight MLP designed to satisfy the computational and memory constraints of the Crazyflie platform.

The network takes the three normalized barometer signals as input and outputs the estimated whisker depth. It consists of three hidden layers with 32 neurons each, followed by a single linear output layer. The resulting model contains 2,273 trainable parameters for each whisker. This architecture provides sufficient capacity to capture the nonlinear relationship between barometer readings and whisker depth while remaining lightweight enough for real-time onboard inference.

We have added the architecture and parameter count to the revised manuscript and Supplementary Information.

**Revision in the paper:**
Page S10 and S13, Supplementary Text, lines 930 – 939 and Table S2:
using different models on Dataset1. For all compared sensor models, hyperparameters were selected on the training set using 5-fold cross-validation. Table. S2 summarizes the hyperparameter search spaces and the selected values for all compared models.

First, we evaluate the sensor model, comparing a deep learning approach (MLP) with traditional machine learning models, including Polynomial Fitting (PolyFit), Gaussian Process Regression

Table S2: Hyperparameter search spaces and selected values for all compared sensor models. For all methods, hyperparameters were selected on the training set using 5-fold cross-validation.

| Model | Hyperparameter | Search range | Selected value | |
|---|---|---|---|---|
| | | | Left whisker | Right whisker |
| PolyFit | Polynomial degree | $\{1, 2, \ldots, 10\}$ | 1 | 1 |
| RFR | Number of trees | $\{100, 200, 300\}$ | 300 | 100 |
| | Maximum tree depth | $\{\text{None}, 5, 10, 20\}$ | 5 | 5 |
| | Minimum samples for split | $\{2, 5, 10\}$ | 5 | 10 |
| | Minimum samples per leaf | $\{1, 2, 4\}$ | 2 | 4 |
| GPR | Kernel amplitude $c$ | $\{1, 2, \ldots, 10\}$ | 6 | 9 |
| | RBF length scale $l$ | $\{50, 100, \ldots, 10000\}$ | 2450 | 4600 |
| MLP | Hidden units per layer | $\{16, 32\}$ | $(32, 32, 32)$ | $(32, 32, 32)$ |
| | Batch size | $\{32, 64, 128\}$ | 32 | 32 |
| | Learning rate | $\{10^{-4}, 10^{-3}\}$ | $10^{-3}$ | $10^{-3}$ |
| | Dropout | $\{0, 0.1, 0.5\}$ | 0.1 | 0.1 |
| | Regularization | $\{0, 10^{-4}, 10^{-3}, 10^{-2}\}$ | $10^{-3}$ | $10^{-2}$ |

(GPR), and Random Forest Regression (RFR). For each whisker, the MLP takes the three normalized barometer signals as input and outputs a scalar depth estimate. The network consists of three hidden layers with 32 neurons each, followed by a linear output layer, resulting in 2,273 trainable parameters. As shown in the results, the MLP model significantly outperforms the traditional machine learning approaches.

## Comment 3

**Reviewer comment:**

How sensitive is the model to whisker replacement or barometer variability? The paper mentions calibration challenges. Is per-device retraining required?

**Response:**

Thank you for raising this important point. The mapping between barometer signals and whisker deflection is sensitive to variations in whisker geometry and sensor characteristics. Because the whiskers are lightweight, highly compliant, and sensitive to small mechanical differences, manufacturing tolerances and barometric inconsistencies can shift the sensor-to-depth mapping across devices.

In the current system, direct model transfer across devices is therefore limited, and per-device recalibration is required to achieve the best performance. In practice, when a whisker or sensing unit is replaced, we perform a short recalibration procedure using approximately 5–6 sweeping

motions with external ground-truth sensing, which takes about 15–20 minutes. Thus, the current pipeline does not yet support calibration-free replacement or zero-shot transfer between devices.

We have clarified this point in the revised manuscript. In particular, the calibration challenge mentioned in the paper refers to the difficulty of scaling this per-device recalibration procedure to large numbers of devices, since the current approach still depends on controlled experiments and external ground-truth sensing.

**Revision in the paper:**
Page 21, Results, lines 383 – 391:
One limitation of our method is drift accumulation during extended periods of time in tactile sweeping, which can degrade whisker depth estimation, especially under disturbances that increase dynamic effects in aerial navigation. Incorporating time-series modeling to predict drift alongside tactile measurements may help mitigate this issue. Increasing the number of whiskers could improve obstacle avoidance, but adds weight, which is critical for small drones. Scalable fabrication and calibration remain open challenges, as barometric inconsistencies and small mechanical variations hinder direct model transfer across devices. In the current setup, achieving the best performance requires a short per-device recalibration procedure based on approximately 5–6 controlled sweeps with external ground-truth sensing, which takes about 15–20 minutes. Future transfer-learning approaches with brief multi-directional sweeps, ideally requiring little or no labeled data, may improve scalability.

## Comment 4

**Reviewer comment:**
In active exploration, what is the computational cost of GPIS onboard? How close is the system to memory limits during exploration?

**Response:**
Thank you for this important question. We have added a more explicit analysis of both memory usage and computational cost for the onboard GPIS module in the revised manuscript and Supplementary Information.

The Crazyflie platform uses an STM32F405 microcontroller with 192 kB of RAM, of which the base firmware occupies approximately 132 kB. For the whisker-based autonomous exploration system, we allocate about 34 kB of memory. Within this allocation, TDORC requires approximately 0.2 kB, and the tactile depth estimator (MLP + KF) occupies about 9.6 kB. For GPIS-based active perception, we limit the onboard representation to at most 50 environmental data points and a $10 \times 10$ grid, which requires approximately 20.9 kB of memory. Importantly, the tactile depth estimator and the penalty-based active perception module are not executed simultaneously, so their temporary working memory can be reused rather than allocated concurrently. Under this configuration, the system remains within the onboard memory budget, with approximately 26 kB of free memory remaining (Fig. S11).

Regarding computational cost, because the GPIS operates on a bounded dataset (maximum 50 points), the kernel matrices remain small and the onboard update remains lightweight. On the STM32F405, the combined GPIS update time (training + inference) is approximately 0.09 s for 20 points, 0.14 s for 30 points, 0.21 s for 40 points, and 0.29 s for 50 points. In practice, GPIS is not executed at every control cycle. The full onboard exploration pipeline runs at 50 Hz, whereas GPIS is triggered intermittently during active exploration, for example after the completion of a sweeping trial. This asynchronous execution keeps the computational load compatible with real-time onboard operation.

We have revised the manuscript to clarify both the memory budget and the computational cost, and to make the remaining system margin more explicit.

**Revision in the paper:**
Page S22 and S23, Supplementary Text, lines 1040 – 1064:

**Memory usage and computational cost**

We use an STM32F405 microcontroller with 192 kB of RAM, of which the base Crazyflie firmware occupies approximately 132 kB (Fig. S12). The whisker-based autonomous exploration system uses about 34 kB of additional memory. Within this budget, the Tactile Drift Online Recurrent Compensation (TDORC) module requires approximately 0.2 kB, while the tactile depth estimator occupies around 9.6 kB, including the neural network and Kalman filter (KF) fusion parameters. Since the two whisker-based depth estimators are cross-called, only one instance of this memory is required.

For the GPIS-based active perception module, we limit the system to 50 training data points and a $10 \times 10$ grid representation. Under this configuration, the GPIS module requires approximately 20.9 kB of memory, which is sufficient for reconstructing small-scale environments such as indoor rooms. Importantly, the penalty-based active perception module and the tactile depth estimator are not executed simultaneously. Their temporary working memory can therefore be reused rather than allocated concurrently, improving overall memory efficiency.

Under the current configuration, the system remains within the onboard memory budget and retains approximately 26 kB of free RAM. When running aerial tactile exploration without the active perception module, an additional 11.3 kB of memory is available.

In addition to memory usage, we evaluate the computational cost of the GPIS module. Because the system is constrained to a bounded dataset (maximum 50 points), the resulting kernel matrices remain small and enable efficient onboard computation. On the STM32F405, the combined GPIS update (training and inference) takes approximately 0.09 s for 20 points, 0.14 s for 30 points, 0.21 s for 40 points, and 0.29 s for 50 points. In practice, GPIS updates are not executed at every control cycle. The full onboard exploration pipeline runs at 50 Hz, while GPIS is triggered intermittently during active exploration. This asynchronous execution keeps the computational load compatible

with real-time onboard operation.

### Comment 5

**Reviewer comment:**
The process model assumes static obstacles and locally planar surfaces. How does performance degrade with curved or moving objects?

**Response:**
Thank you for this insightful question. We agree that the current process model assumes static obstacles and locally planar surfaces, and that evaluating the effect of curved or moving objects would strengthen the paper.

To address this point, we added additional experiments on curved surfaces and a moving surface. Specifically, we evaluated wall-following sweeping on three curved acrylic surfaces: a concave surface with relatively low curvature, a concave surface with higher curvature, and a convex surface with higher curvature. In each case, the drone performed 10 seconds of wall-following sweeping using the FSM-based aerial tactile navigation framework. We further evaluated wall following on a moving transparent surface.

The results show that the system remains capable of following curved and moving surfaces, but performance degrades as curvature increases and the interaction becomes more dynamic. In the low-curvature concave and high-curvature convex cases, oscillatory motion appears near the end of the trial. A likely reason is that the current FSM controller does not regulate the contact state as smoothly as a continuous feedback controller: once the drone moves outside the threshold band, it tends to oscillate around the upper and lower bounds instead of returning smoothly to the desired range. Under these higher-dynamic conditions, tactile perception also becomes less reliable, which further degrades reconstruction quality.

For the moving surface, the controller exhibits similar oscillatory behavior, but the drone still successfully follows the moving obstacle and continues forward motion around it. These results suggest that the current system remains usable under moderate curves and moderate dynamic obstacles assumption, but that stronger curvature and obstacle motion increase controller–perception coupling and reduce tracking quality.

We have revised the manuscript to clarify this limitation and added the new experiments to the Supplementary Information. We also note in the revised text that future work will investigate whisker-based tactile servoing for more stable curved-surface tracking and tighter integration of tactile perception and control.

**Revision in the paper:**
Page 13, Results, lines 267 – 271:
We further evaluated the system under violations of the locally planar and static-obstacle assumptions, including curved surfaces and a moving surface. The system remained capable of wall following under moderate curvature and object motion, although oscillations increased and object

reconstruction quality degraded in more dynamic cases. Detailed results are provided in Supplementary Fig. S9 and Movies S8.

Page S18 – S20, Supplementary Text, lines 998 – 1032 and Figure S9:

**Robustness to curved and moving surfaces**

To evaluate the effect of violating the locally planar and static-obstacle assumptions used in the process model, we conducted additional wall-following experiments on curved surfaces and a moving surface, as shown in Fig. S9 and Movies S8.

For the curved-surface experiments, we used three transparent acrylic surfaces with different curvatures: a concave surface with relatively low curvature, a concave surface with higher curvature, and a convex surface with higher curvature. In each case, the drone performed 10 s of wall-following sweeping using the same finite-state-machine (FSM)-based aerial tactile navigation framework as in the *Aerial tactile navigation via invisible walls* section (Fig. S5). Fig. S9a–c shows representative image sequences of these three experiments. The corresponding top-view reconstructions are shown in Fig. S9d–f. In each plot, we visualize the drone trajectory and orientation together with the reconstructed surface and the approximate surface. The reconstructed surface is obtained by averaging the left and right whisker depth estimates from the MLP+KF model. The approximate surface is obtained by fitting a cubic spline to control points recorded by the OptiTrack system. These results show that the current system can still follow curved surfaces and recover approximate local geometry. In the initial stable wall-following stage, the reconstruction remains relatively accurate. However, as curvature increases and the interaction becomes more dynamic, oscillatory motion emerges, and the reconstruction quality degrades noticeably during the oscillatory phase.

In the low-curvature concave case and the high-curvature convex case, oscillatory motion appears near the end of the trial, as visible in Fig. S9a,c and Movie S8. A likely cause is the current FSM controller, which does not regulate the contact state as smoothly as a continuous feedback controller. Once the drone moves outside the threshold band, it tends to oscillate around the upper and lower bounds instead of returning smoothly to the desired band. Under these higher-dynamic conditions, tactile perception also becomes less reliable, which further degrades reconstruction quality. Nevertheless, the system remains capable of completing curved-surface wall following under these conditions.

We further evaluated the system on a moving surface, as shown in Fig. S9g and Movie S8. Although the controller again exhibits oscillatory behavior similar to that observed in the curved-surface experiments, the drone still successfully follows the moving obstacle and continues forward motion around it.

These experiments indicate that the current system remains usable under moderate violations of the static and locally planar assumptions, but that stronger curvature and obstacle motion increase controller–perception coupling and degrade performance. Future work will investigate whisker-

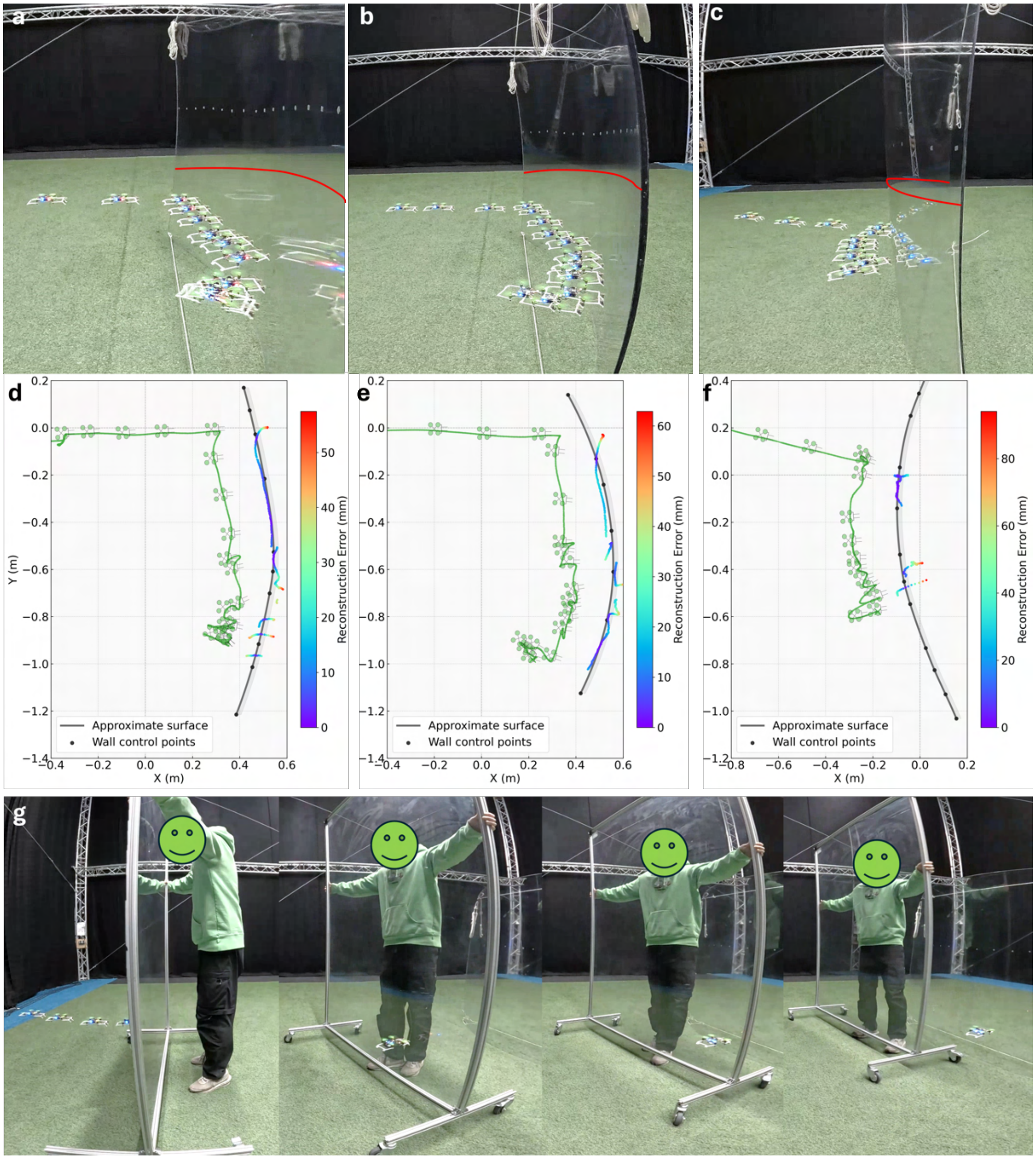


Figure S9: Experimental results of aerial tactile navigation on curved and moving surfaces. (a–c) Whiskered drone performing 10 s wall-following sweeping on a low-curvature transparent concave surface, a high-curvature transparent concave surface, and a high-curvature transparent convex surface, respectively. (d–f) Corresponding top-view trajectories and surface reconstructions for the three curved-surface experiments. The reconstructed surface is obtained from the average of the left and right whisker estimates using the MLP+KF model, and the approximate surface (GT) is obtained by fitting a cubic spline to OptiTrack control points. (g) Whiskered drone performing wall following on a moving surface.

based tactile servoing to achieve more stable curved-surface tracking and tighter integration of tactile perception and control.

## Comment 6

**Reviewer comment:**
It would be helpful to report energy consumption or flight time reduction due to whisker payload and computation.

**Response:**
Thank you for this suggestion. We agree that such insights would help in evaluating the practicality of the system.

To address this point, we added additional results comparing flight time under three configurations: (1) without extra payload, (2) with the whisker sensing suite (including wiring and propeller guard) and data transmission, and (3) with the full onboard tactile perception pipeline active. The measured flight times are 11.26 min (no extra payload), 8.52 min (whisker suite), and 7.18 min (whiskers with onboard perception), corresponding to reductions of approximately 24% and 36%, respectively, relative to the no-extra-payload baseline.

Importantly, the onboard tactile perception pipeline is not continuously active, but is triggered intermittently upon contact events. This event-driven design reduces unnecessary computation and helps limit the overall energy cost of onboard tactile processing. These results therefore highlight the expected trade-off between sensing capability and flight endurance. Despite this reduction, the system remains fully onboard and capable of real-time tactile perception, navigation, and control within the resource constraints of a 44.1 g aerial platform.

We have added a new supplementary figure and revised the manuscript accordingly.

**Revision in the paper:**
Page S23, Supplementary Text, lines 1065 – 1079 and Figure S13:

### Flight time analysis

We provide a detailed analysis of the impact of the whisker sensing system and onboard computation on flight time. Three configurations are evaluated: (1) no extra payload, (2) whisker suite including wiring and propeller guard with data transmission, and (3) whiskers with the full onboard tactile perception pipeline.

The measured flight times are 11.26 min, 8.52 min, and 7.18 min, respectively (Fig. S13), corresponding to reductions of approximately 24% and 36% compared to the no-extra-payload baseline.

Importantly, the tactile perception pipeline is not continuously active. Instead, it is triggered intermittently upon contact events. This event-driven design reduces unnecessary computation

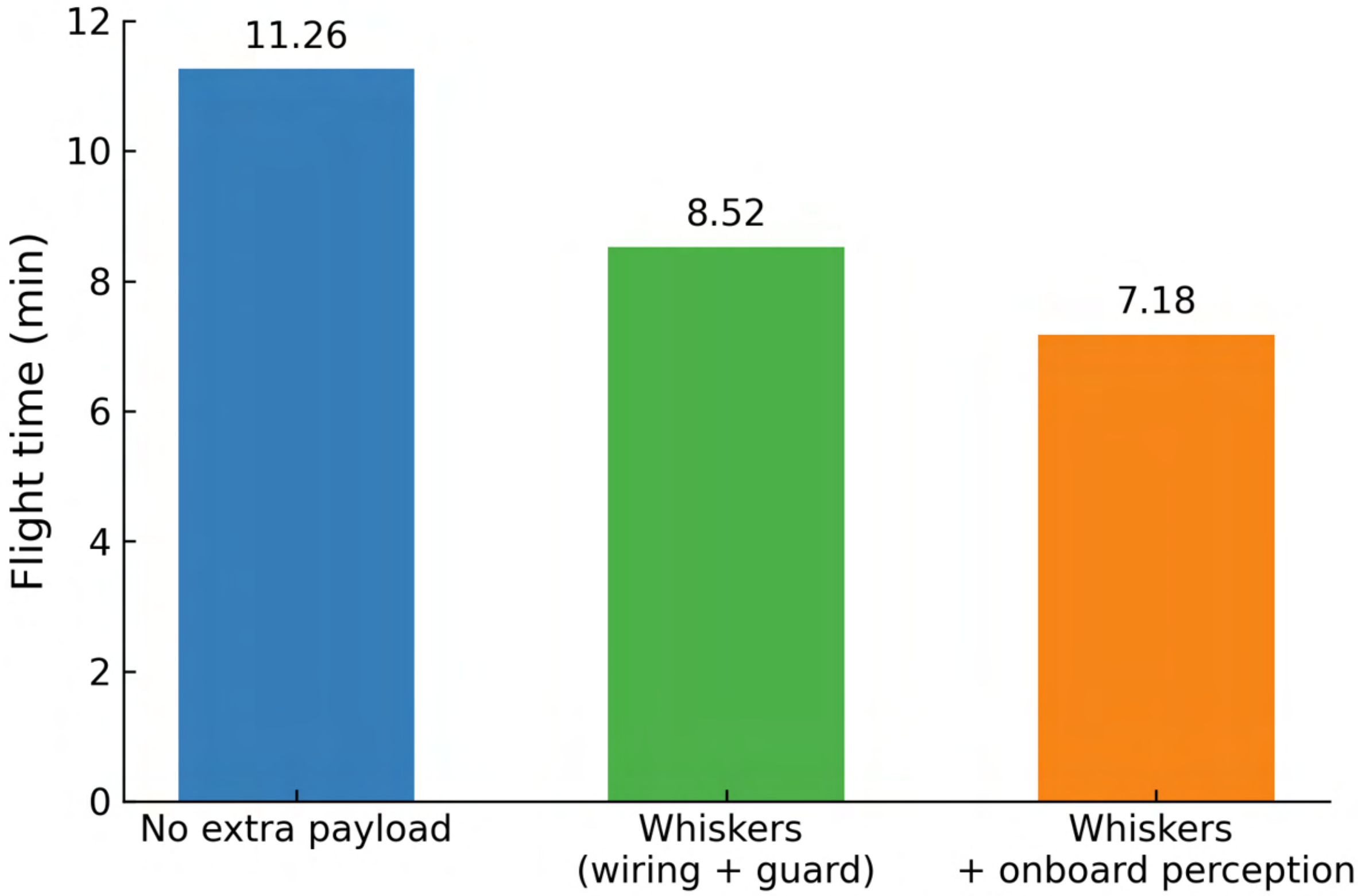


Figure S13: Flight time under different configurations. Comparison of flight time for (i) no extra payload, (ii) whisker suite with wiring and propeller guard, and (iii) whiskers with onboard tactile perception. The perception pipeline is activated intermittently upon contact events (every 30 s).

and helps limit the overall energy consumption on the tiny drone.

The reduction in flight time is therefore mainly attributed to the additional payload (whisker structure, wiring, and protection) and the intermittent activation of onboard processing. Despite this trade-off, the system remains capable of fully onboard tactile perception and navigation within the tight mass constraints of a 44.1 g nano drone.

## References


[1] Bitcraze. Crazyflie 2.1 brushed. `https://www.bitcraze.io/products/crazyflie-2-1-plus/`, 2026. Accessed: 2026-04-16.

[2] Bitcraze. Crazyflie 2.1 brushless. `https://www.bitcraze.io/products/crazyflie-2-1-brushless/`, 2026. Accessed: 2026-04-16.

[3] Bitcraze. Pwm to thrust. `https://www.bitcraze.io/documentation/repository/crazyflie-firmware/master/functional-areas/pwm-to-thrust/`, 2026. Crazyflie firmware documentation, accessed 2026-04-16.

[4] Anton Bredenbeck, Teaya Yang, Salua Hamaza, and Mark W Mueller. A tactile feedback approach to path recovery after high-speed impacts for collision-resilient drones. *arXiv preprint arXiv:2410.14249*, 2024.

[5] Paolo De Petris, Huan Nguyen, Mihir Kulkarni, Frank Mascarich, and Kostas Alexis. Resilient collision-tolerant navigation in confined environments. In *2021 IEEE International Conference on Robotics and Automation (ICRA)*, pages 2286–2292. IEEE, 2021.

[6] Teresa A Kent, Hannah Emnett, Mahnoush Babaei, Mitra JZ Hartmann, and Sarah Bergbreiter. Identifying contact distance uncertainty in whisker sensing with tapered, flexible whiskers. In *2023 IEEE International Conference on Robotics and Automation (ICRA)*, pages 607–613. IEEE, 2023.

[7] Teresa A Kent, Suhan Kim, Gabriel Kornilowicz, Wenzhen Yuan, Mitra JZ Hartmann, and Sarah Bergbreiter. Whisksight: A reconfigurable, vision-based, optical whisker sensing array for simultaneous contact, airflow, and inertia stimulus detection. *IEEE Robotics and Automation Letters*, 6(2):3357–3364, 2021.

[8] Suhan Kim, Regan Kubicek, Aleix Paris, Andrea Tagliabue, Jonathan P How, and Sarah Bergbreiter. A whisker-inspired fin sensor for multi-directional airflow sensing. In *2020 IEEE/RSJ International Conference on Intelligent Robots and Systems (IROS)*, pages 1330–1337. IEEE, 2020.

[9] Suhan Kim, Camilo Velez, Dinesh K Patel, and Sarah Bergbreiter. A magnetically transduced whisker for angular displacement and moment sensing. In *2019 IEEE/RSJ International Conference on Intelligent Robots and Systems (IROS)*, pages 665–671. IEEE, 2019.

[10] Michael A Lin, Emilio Reyes, Jeannette Bohg, and Mark R Cutkosky. Whisker-inspired tactile sensing for contact localization on robot manipulators. In *2022 IEEE/RSJ International Conference on Intelligent Robots and Systems (IROS)*, pages 7817–7824. IEEE, 2022.

[11] Yash Mulgaonkar, Wenxin Liu, Dinesh Thakur, Kostas Daniilidis, Camillo J Taylor, and Vijay Kumar. The tiercel: A novel autonomous micro aerial vehicle that can map the environment by flying into obstacles. In *2020 IEEE International Conference on Robotics and Automation (ICRA)*, pages 7448–7454. IEEE, 2020.

[12] Karishma Patnaik, Aravind Adhith Pandian Saravanakumaran, and Wenlong Zhang. Tactile-based exploration, mapping, and navigation with collision-resilient aerial vehicles. *IEEE/ASME Transactions on Mechatronics*, 2025.

[13] Ruoyu Wang, Zixuan Guo, Yizhou Chen, Xinyi Wang, and Ben M Chen. Air bumper: A collision detection and reaction framework for autonomous mav navigation. In *2024 IEEE International Conference on Robotics and Automation (ICRA)*, pages 15735–15741. IEEE, 2024.

[14] Chaoxiang Ye, Guido De Croon, and Salua Hamaza. A biomorphic whisker sensor for aerial tactile applications. In *2024 IEEE International Conference on Robotics and Automation (ICRA)*, pages 5257–5263. IEEE, 2024.
