# Supplementary material for: Whisker-based tactile flight for tiny drones
Source: Nat Commun. 2026 Sep 18;17:9743. doi: 10.1038/s41467-026-77366-7 (PMC13588785; doi:10.1038/s41467-026-77366-7)
Supplement: Supplementary file 2 [file 41467_2026_77366_MOESM1_ESM.pdf]

Supplementary Materials for

Whisker-based Tactile Flight for Tiny Drones

Chaoxiang Ye*, Guido de Croon, Salua Hamaza*

*Corresponding authors. Emails: C.Ye@tudelft.nl; S.Hamaza@tudelft.nl

This PDF file includes:

Supplementary Text

Figures S1 to S13

Tables S1 to S3

Other Supplementary Materials for this manuscript:

Supplementary Movies 1 to 8

Supplementary data 1

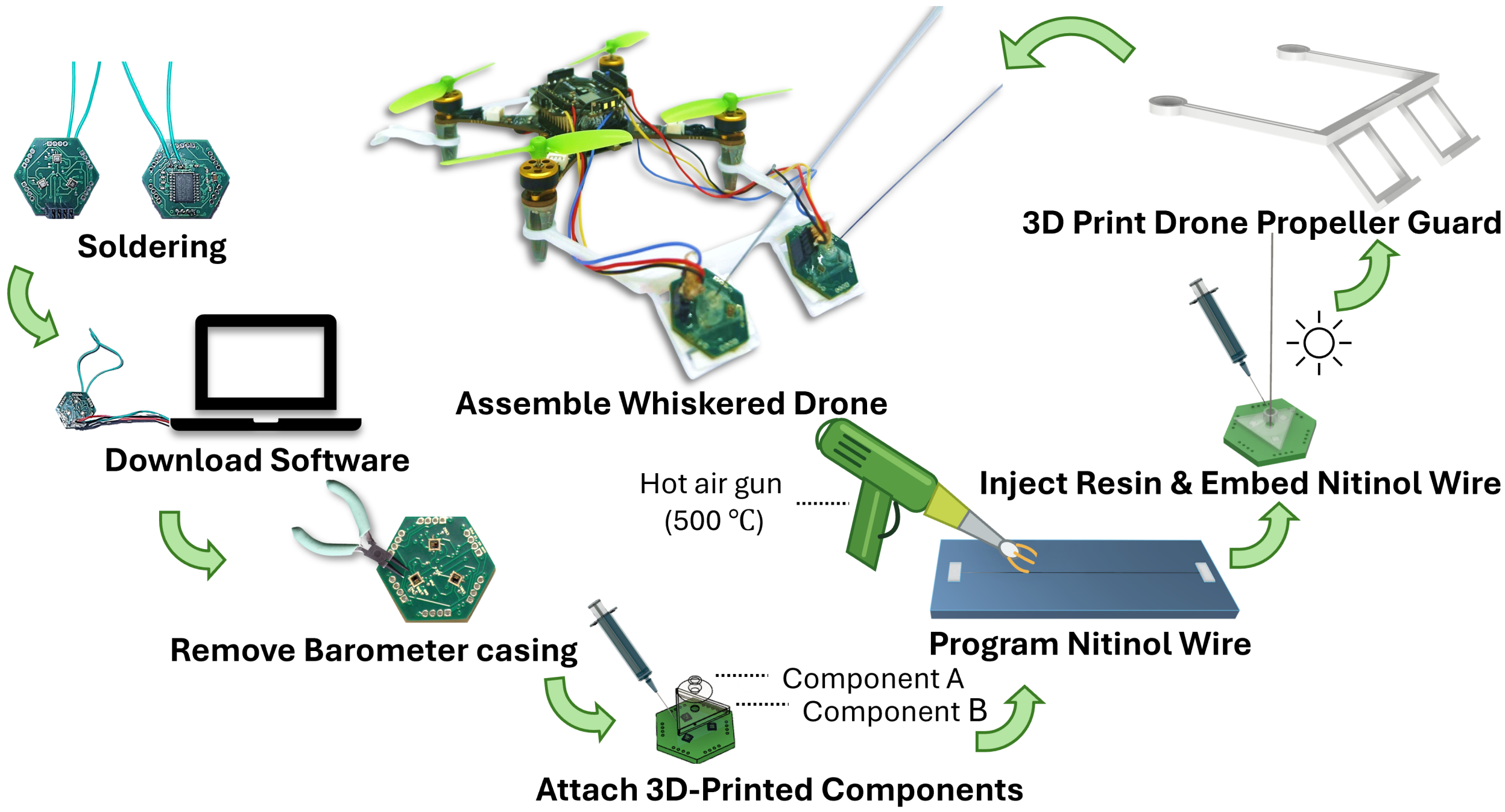

Figure S1: Whisker sensor fabrication process. The transparent casing consists of two components: A and B. Component A is a tubular structure with a bulging base, while Component B is designed to enclose the three barometers and establish their connection to the PCB. Before mounting onto the PCB, these two components must be securely bonded together using UV resin.

Supplementary Text

Whisker design and fabrication

The design of the whisker sensor follows our previous work (*1*) with some minor modifications. Initially, the original PCB was equipped with six UART ports; however, we modified it to include five I^2C ports and one UART port to enhance multi-device communication among the whisker sensors. The single UART port was retained exclusively for flashing software onto the microcontroller. Additionally, we 3D-printed a protective package for the follicle-sinus complex (FSC) using transparent PETG filament. This protective package serves three key functions: first, it ensures the injection of a uniform amount of UV resin, thereby minimizing variations in data distribution caused by manufacturing tolerances (albeit with only a slight improvement); second, it regulates the resin quantity to prevent signal out-of-range issues; and third, it safeguards the FSC, ultimately extending the sensor's lifespan. The protective package comprises two components, as shown in

Fig. S1. Component A is a tube featuring a bulging base resembling a small hill, which is specifically designed to enhance sensor sensitivity. These two components should be securely bonded together using UV resin to form a complete protective package.

The total fabrication process is shown in Fig. S1. We begin by soldering all the electronic components, connecting the wires for software downloading (ISP), and attaching the pin headers for communication. Next, we carefully remove the metal casing surrounding the MEMS device, exposing the sensing element for microforce detection. For programming the nitinol wire, we first secure it in a straight configuration and then apply a hot air gun at approximately 500°C, moving at a controlled velocity of around 5 mm/s to achieve precise shape setting. The transparent 3D-printed package consists of two components, A and B, which are first glued together using UV resin. This assembled package is then attached to the PCB, forming a mold to house the three barometers. The pre-programmed nitinol wire is carefully positioned along the central axis of a plastic tube, ensuring it remains perpendicular to the PCB surface. Using a syringe with a 1.2 mm needle, we inject 1.5 ml of UV resin into the tube, allowing it to flow evenly until a uniform surface is formed within the mold. The structure is then cured under a UV resin lamp to solidify the assembly. Finally, we 3D print a custom drone propeller guard designed to hold two whiskers at a 45° upward angle.

Choice of sensing modality

The choice of barometric sensing is driven by the constraints of aerial tactile exploration rather than by a direct benchmark against alternative modalities. Small aerial robots impose strict limits on payload, onboard computation, and robustness under repeated transitions between contact and free flight. Among the representative alternatives commonly explored in whisker sensing, vision-based whisker sensing typically requires additional imaging hardware and processing, increasing computational load and system footprint (*2, 3*). Magnetic-based approaches often require extra magnets, holders, or alignment structures, which increase integration complexity and size (*4, 5*). In addition, achieving high sensitivity with long, slender whiskers typically requires compliant deformation structures, introducing a trade-off with mechanical robustness (*6*). In contrast, barometric sensing enables a compact, lightweight, and sensitive implementation that is well suited to aerial deployment.

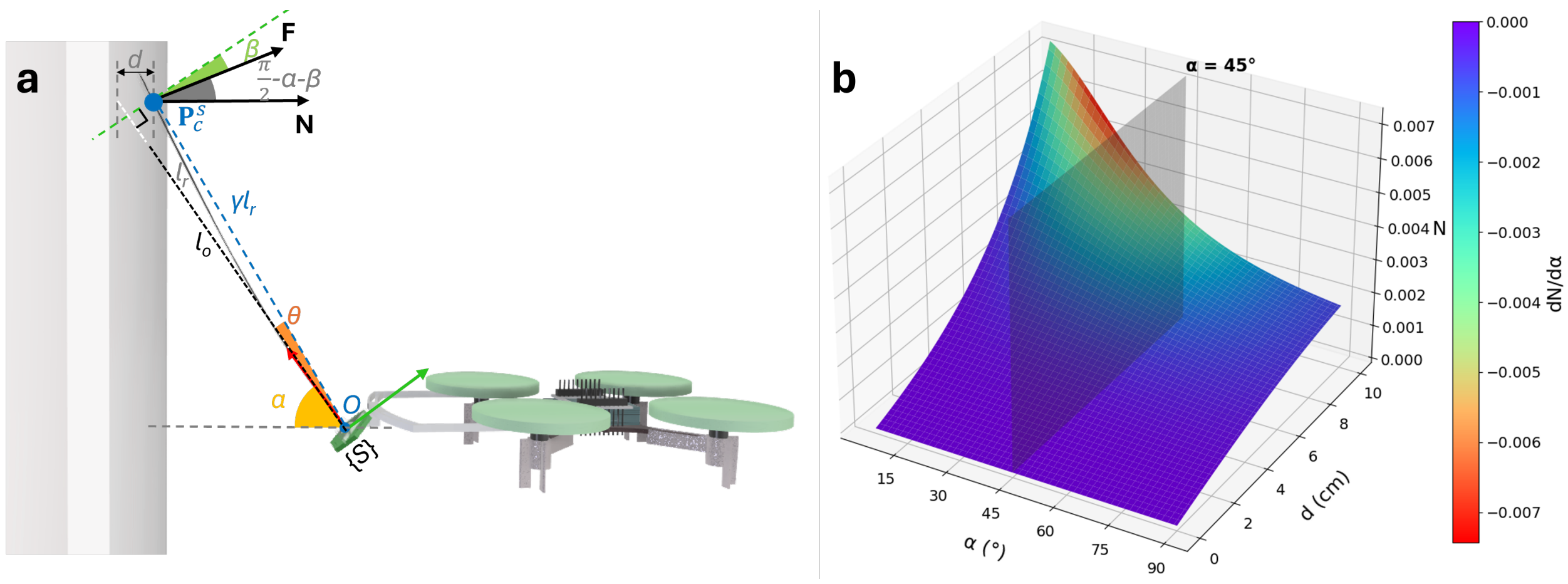

Figure S2: Analysis of normal contact force (N) in whisker-based interaction with a static contact point $\mathbf{P}_C^S$. (a) Schematic of force analysis in drone interaction using PRBM. This diagram illustrates the force interaction in a whisker-based sensor system, where a drone-mounted whisker makes contact with a static contact point $\mathbf{P}_C^S$. The analysis employs the Pseudo Rigid-Body Model (PRBM) to approximate whisker deflection and force distribution. Here, θ represents the pseudo-rigid-body rotation angle, γ is the characteristic radius factor, and β denotes the force ratio that defines the vertical contact force F at point $\mathbf{P}_C^S$, measured relative to the x-axis of the sensor frame $\{S\}$. (b) Numerical results of normal force (N) variation with placement angle (α) and contact depth (d). This plot presents the computed relationship between whisker placement angle α, contact depth d, and the resulting normal force N. The results indicate that the normal force N decreases as the placement angle α increases for a given contact depth d.

Theoretical analysis for whisker placement selection

We aim to determine the whisker placement that minimizes the contact force for a given contact depth d, Fig. S2a. To validate our hypothesis in the case of larger deflections, we employ Pseudo Rigid-Body Modeling (PRBM). The 1R PRBM is a single-degree-of-freedom system consisting of two rigid links connected by a revolute joint with a torsion spring. In this model, the shorter rigid link is fixed, while the longer one rotates.

Let l_o denote the original length of the whisker and l_r the initial length of the flexural beam, measured between the contact point $\mathbf{P}_c^S$ in the sensor frame $\{S\}$ and the root $\mathbf{O}$. The rotation angle of the rigid link in $\{S\}$ is represented by θ, and the torsion spring constant by K. The rotating

rigid link has a length of γl_r, where γ is the characteristic radius factor. The vertical force F at the contact point $\mathbf{P}_c^S$ is related to the rotation angle θ by the following equation:

$$F = \frac{K\theta}{\gamma l_r(\cos\theta + n\sin\theta)}, \tag{S1}$$

where $n = tan\beta$ represents the force ratio between the horizontal and vertical forces in , given by $\operatorname{arccot}\beta$. The parameter β has been derived and validated in (*7*) as:

$$\beta \approx \frac{3}{2}\theta. \tag{S2}$$

Since the contact point moves within $\{S\}$, we must recalculate the original length of the flexural beam l_r based on the contact point $\mathbf{P}_c^S$ and the characteristic radius γ to ensure that the force is applied at the free end of the beam. In this case, the contact point in $\{S\}$ is represented as:

$$\mathbf{P}_c^S = \begin{bmatrix} P_x^S \\ P_y^S \end{bmatrix} = \begin{bmatrix} l_o - d\cos\alpha \\ d\sin\alpha \end{bmatrix}. \tag{S3}$$

The original length of the flexural beam in $\{S\}$ is then calculated as:

$$l_r = \frac{\|\mathbf{P}_c^S\|}{\gamma} = \frac{\sqrt{(P_x^S)^2 + (P_y^S)^2}}{\gamma}. \tag{S4}$$

Substituting Equation (S3), we obtain:

$$l_r = \frac{\sqrt{(l_o - d\cos\alpha)^2 + (d\sin\alpha)^2}}{\gamma}. \tag{S5}$$

The rotation rad θ in $\{S\}$ can be determined from the contact point $\mathbf{P}_c^S$ as follows:

$$\theta = \arctan\left(\frac{d\sin\alpha}{l_o - d\cos\alpha}\right). \tag{S6}$$

To calculate K and γ, we use the functions:

$$\gamma = 0.841655 - 0.0067807n + 0.000438n^2 \tag{S7}$$

$$K = \frac{\gamma EI(a_0 - a_1 n + a_2 n^2 - a_3 n^3 + a_4 n^4)}{l_r}, \tag{S8}$$

where $a_0 = 2.654855$, $a_1 = 0.509896 * 10^{-1}$, $a_2 = 0.126749 * 10^{-1}$, $a_3 = 0.142039 * 10^{-2}$, $a_4 = 0.584525 * 10^{-4}$ when $0.5 < n < 10$, which implies $5.7 < \theta < 63.4$, which is satisfied in our experiments. Then we get:

$$F \propto \frac{(a_0 - a_1 n + a_2 n^2 - a_3 n^3 + a_4 n^4)\theta\gamma^2}{((l_o - d\cos\alpha)^2 + (d\sin\alpha)^2)(cos\theta + nsin\theta)}. \tag{S9}$$

$$N \propto \frac{(a_0 - a_1 n + a_2 n^2 - a_3 n^3 + a_4 n^4)\theta\gamma^2}{((l_o - d\cos\alpha)^2 + (d\sin\alpha)^2)(cos\theta + nsin\theta)sin(\frac{3}{2}\theta + \alpha)} \tag{S10}$$

We then obtain a numerical results between the placement angle α, contact depth d and the normal force N shown in Fig. S2b. It can be seen that the normal force N decreases with increasing placement angle α at the same contact depth d. Additionally, we introduce a reference plane at $\alpha = 45°$, highlighting that beyond this angle, the rate of decrease in N becomes less steep.

Preliminary experiments of whisker placements on the Crazyflie 2.1 brushed platform

We conducted preliminary experiments of whisker placement angle on the Crazyflie 2.1 brushed platform to qualitatively assess how the contact location in space influences the interaction stability during wall sweeping maneuvers.

The brushed platform has a mass of 27 g and a recommended maximum payload of about 15 g. Its smaller practical thrust margin made less favourable whisker placements more evident, as the platform was more prone to crashes caused by contact-induced instabilities.

In these preliminary trials, five whisker placement angles (−15°, 0°, 15°, 30°, and 45°) were tested at a contact depth of $d = 3$ cm while sweeping along a smooth wall at 0.1, 0.2, and 0.5 m/s (Fig. S3b–g). Among the tested configurations, the 45° placement showed the most stable interaction across the tested velocities, whereas the 15° configuration, which shifted close to 0° due to gravity, showed the highest crash tendency. Crashes were primarily associated with excessive yaw moment deviation that the controller could not compensate in time. Video compilations are also provided in Supplementary Movie 1.

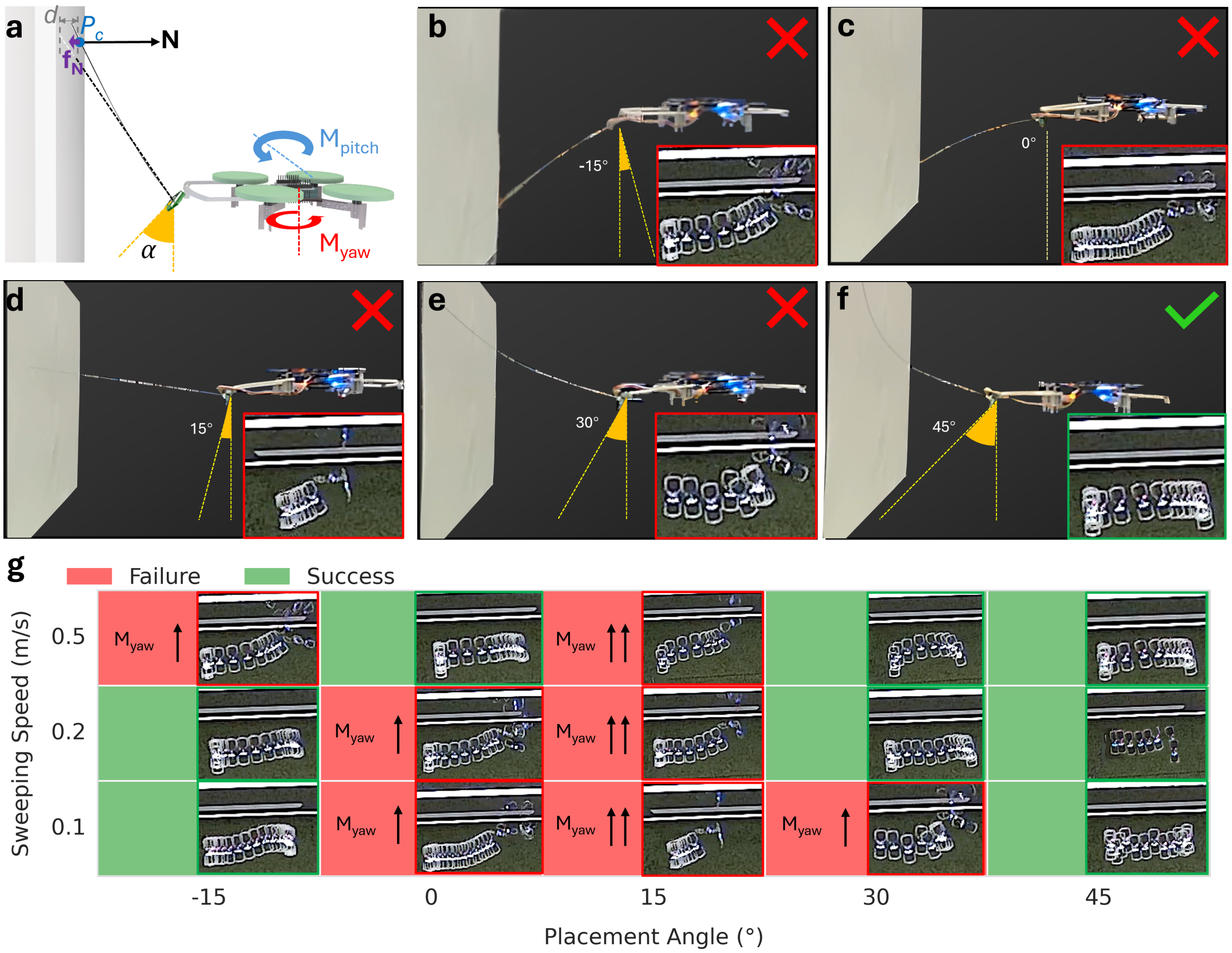

Figure S3: Effect of whisker placement angle on drone stability during wall-sweeping interactions on the Crazyflie 2.1 brushed platform. (a) Schematic illustration of the forces and moments acting on the drone during whisker sweeping. During contact with a vertical obstacle, the whisker generates a normal force N, which induces a friction force f_N. The placement angle α influences the resulting M_{pitch} and M_{yaw}. (b–f) Representative wall-sweeping trials with whiskers mounted at $-15°$, $0°$, $15°$, $30°$, and $45°$ in the pitch plane. Due to gravity, the tip of the whisker naturally tilts downward by approximately $10°$ from the preset angle. The top-view inset in the lower right corner illustrates that crashes primarily occur when the drone is unable to compensate for M_{yaw}. (g) Exploratory results showing yaw deflection and success or failure outcomes for different whisker placement angles at sweeping velocities of 0.1, 0.2, and 0.5 m/s. Among the tested configurations, the $45°$ placement showed the most stable interaction in these preliminary trials.

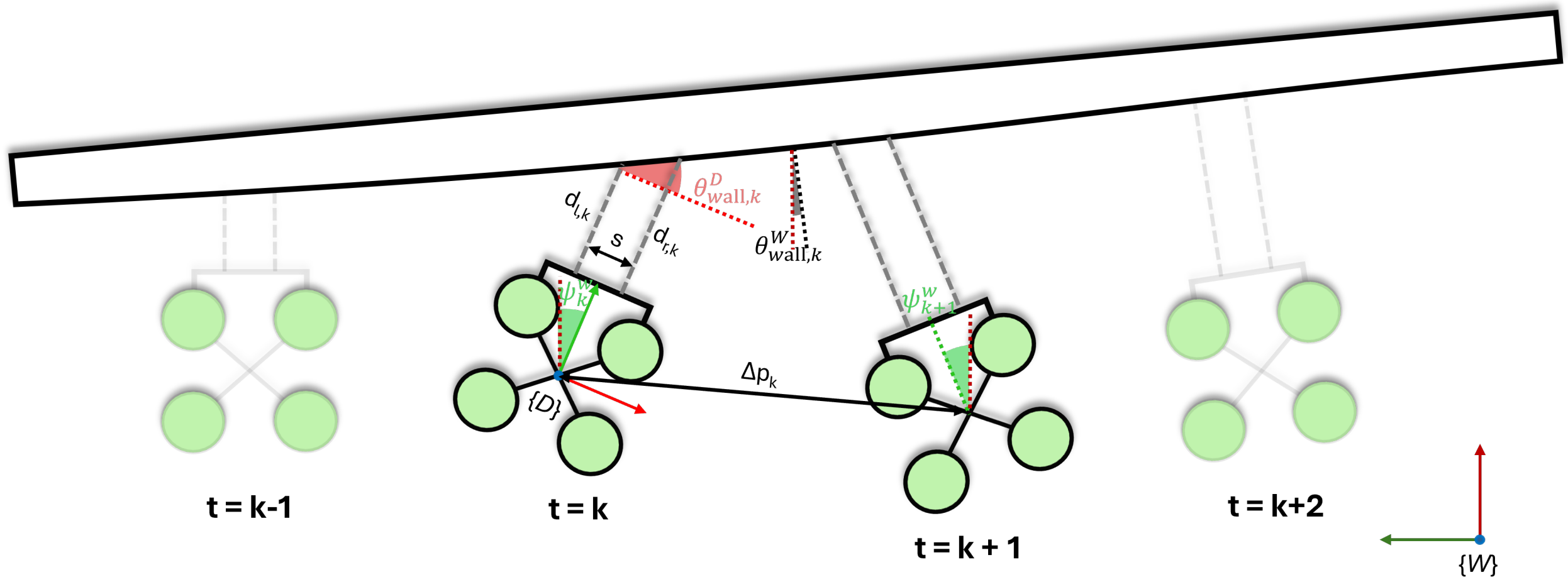

Figure S4: The process model for tactile depth estimation. At time $t = k$, we compute $\theta^{D}_{\text{wall},k}$ using $d_{l,k}$ and $d_{r,k}$, which are obtained by fusing the sensor model outputs m_l and m_r at that moment with the predicted process model outputs $d^{-}_{l,k}$ and $d^{-}_{r,k}$ from the previous time step, along with the sensor distance s. Using this computed $\theta^{D}_{\text{wall},k}$, the drone's positional change Δp_{k+1}, and the updated orientation ψ^{W}_{k+1}, we predict the next process model outputs $d^{-}_{l,k+1}$ and $d^{-}_{r,k+1}$ for the subsequent time step $k + 1$.

Process model for tactile depth estimation

This graphical supplement Fig. S4 is based on the process model presented in the main text, which visualizes the parameters. The slight bump in the wall is almost negligible, as we assume the wall to be locally planar in our model. At time $t = k$, we compute $\theta^{D}_{\text{wall},k}$ in the drone frame D using $d_{l,k}$ and $d_{r,k}$, which are derived by fusing the sensor model outputs m_l and m_r at that moment with the predicted process model outputs $d^{-}_{l,k}$ and $d^{-}_{r,k}$ from the previous time step, along with the sensor distance s. Using the computed $\theta^{D}_{\text{wall},k}$, the drone's positional change Δp_{k+1}, and the updated orientation ψ^{W}_{k+1}, we predict the next process model outputs $d^{-}_{l,k+1}$ and $d^{-}_{r,k+1}$ for the subsequent time step $k + 1$. In this illustration, we exaggerate the drone's state change between the two time points for clarity; however, in practice, the change is much smaller due to the drone's low speed and high frequency.

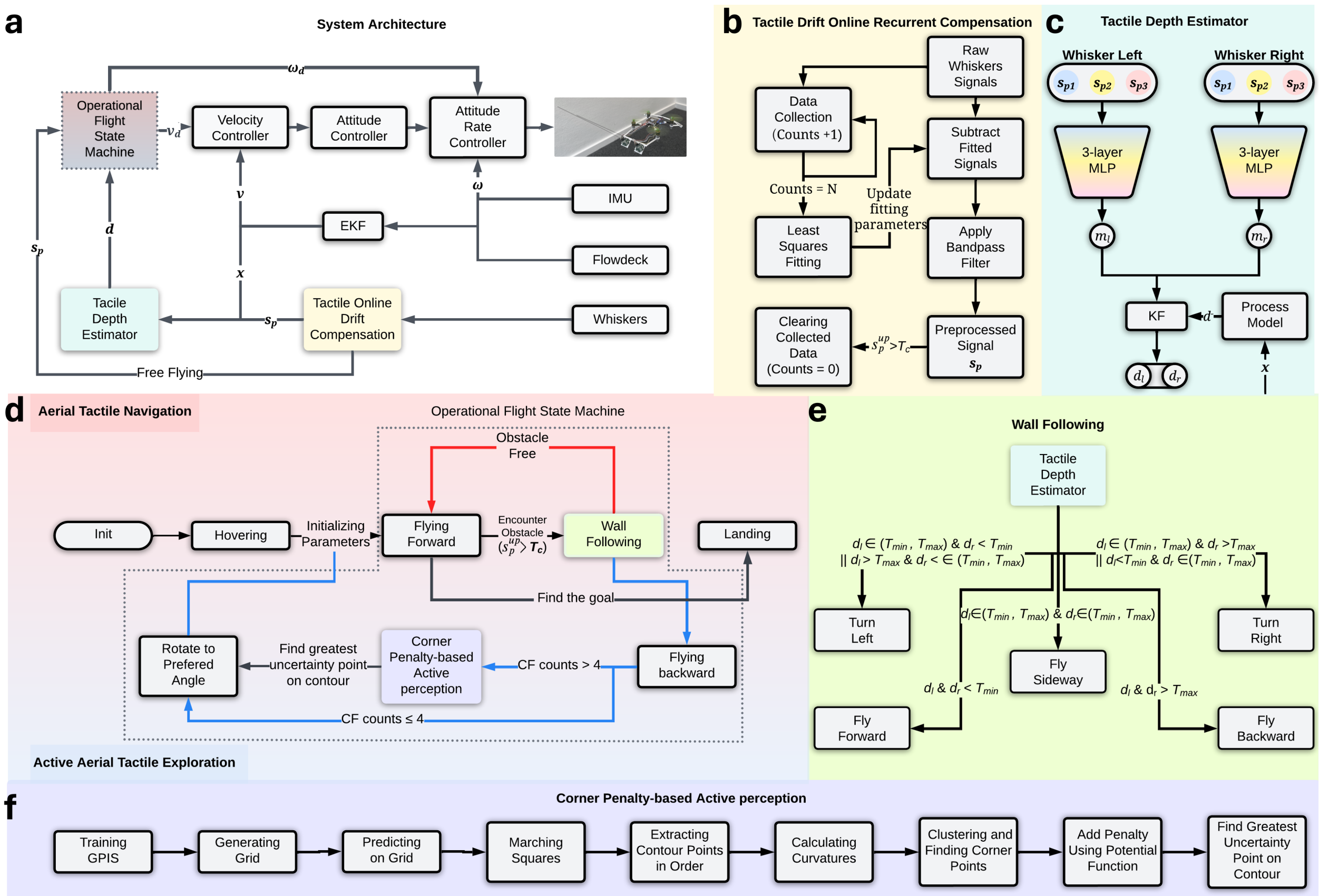

Figure S5: Overview of our whiskered drone system pipeline (full version). (a) System architecture diagram. (b) The TODRC module for compensating tactile drift. (c) The tactile depth estimation module, which fuses the sensor model and process model to predict left and right whisker depths. (d) The aerial tactile navigation module (highlighted in red), based on wall-following, and the active aerial tactile exploration module (highlighted in blue), which integrates wall-following and corner penalty-based active perception. The shared components used in both modules are represented with black arrows. (e) Wall-Following Module: A finite-state machine consisting of five states, designed to guide the drone along surfaces based on tactile feedback.(f) Corner penalty-based active perception: A module leveraging geometric information and uncertainty from the GPIS-predicted environment. By identifying high-curvature points and clustering them into corners, a penalty potential function is applied to encourage the drone to avoid hazardous areas. This penalty, combined with uncertainty-based exploration, ensures safe and efficient navigation in complex environments.

Whiskered drone system pipeline

In Fig. S5, we present the complete pipeline of the whiskered drone. Modules Fig. S5a, b, c, and d are identical to those described in the main text, while Fig. S5e represents the wall-following module, which operates based on five distinct states: *Fly Sideways*, *Fly Forward*, *Fly Backward*, *Turn Left*, and *Turn Right*, determined by the estimated distance to the obstacle. In this mode, the sideways velocity (V_{sideway}) is set to V_{max}, while the forward (V_{forward}) and backward (V_{backward}) velocities are set to $V_{\text{max}}/2$ to enhance stability during wall following. The turn rate is set to ω_{max}. The conditions for each turn maneuver are illustrated in the figure, based on the intervals in which d_l and d_r fall. Fig. S5f illustrates the corner penalty-based active perception module, which corresponds to the relevant section in Methods. Using GPIS (Gaussian Process Implicit Surfaces), we predict the value of each point on a generated grid and extract contour segments where $z = 0$ using the marching squares method. These contour segments are then connected sequentially as contour points. From the extracted contour points, we compute curvature values and identify points where the curvature exceeds a set threshold. A clustering method is then applied to detect corner points, where a penalty potential function is imposed to encourage the drone to avoid hazardous areas. This penalty mechanism, combined with uncertainty-based exploration, ensures safe and efficient navigation in complex environments.

Whisker-based tactile depth estimation supplemantary results

Table. S1 presents the results of whisker-based tactile depth estimation using different models on Dataset1. For all compared sensor models, hyperparameters were selected on the training set using 5-fold cross-validation. Table. S2 summarizes the hyperparameter search spaces and the selected values for all compared models. First, we evaluate the sensor model, comparing a deep learning approach (MLP) with traditional machine learning models, including Polynomial Fitting (PolyFit), Gaussian Process Regression (GPR), and Random Forest Regression (RFR). For each whisker, the MLP takes the three normalized barometer signals as input and outputs a scalar depth estimate. The network consists of three hidden layers with 32 neurons each, followed by a linear output layer, resulting in 2,273 trainable parameters. As shown in the results, the MLP model significantly outperforms the traditional machine learning approaches. Next, we assess the effectiveness of KF

fusion, which, as discussed in the Whisker-based tactile depth estimation section, demonstrates better performance compared to using the sensor model alone.

We further provide a detailed analysis of the impact of KF fusion, including deviation plots and reconstruction plots for both whiskers on Dataset 1 and Dataset 2, Fig. S6. For surface reconstruction, we take the average of the left and right whisker estimates as input. As discussed in the main text, the fusion process model effectively mitigates sensor hysteresis effects. The deviation and reconstruction plots further confirm that the fusion method yields predictions more closely aligned with the true values. Unlike the raw sensor response, which primarily follows the drone's motion, the reconstructed trajectory more accurately represents the actual object surface. Additionally, in the bottom-right corner of the deviation plots, we present histogram plots that illustrate the distribution of errors. These histograms demonstrate that the fused method produces a distribution more centered around zero, indicating reduced deviation. Moreover, the mean error and the 95% confidence interval, marked in the figure, further highlight the improved accuracy and consistency achieved through KF fusion.

Robustness to Post-Contact Oscillations

During tactile flight, when the whisker slides off an obstacle, the system may experience transient oscillations caused by whisker rebound and drone-induced vibrations. These effects introduce high-frequency ringing in the raw (whisker) pressure signals. To evaluate the robustness of the proposed method under such conditions, we analyze representative segments of tactile navigation data, as shown in Fig. S7. For clarity of visualization, we show the top channel of each whisker, defined as the channel experiencing the largest deformation under contact and thus exhibiting the strongest response. This channel is also used for contact detection in our system. In practice, all channels are used in the proposed method, and similar behavior is consistently observed across them.

As highlighted in the shaded gray regions, the raw signals exhibit pronounced oscillations after sliding off the obstacle. In contrast, the TDORC-processed signals remain significantly more stable, effectively attenuating high-frequency components while preserving the informative tactile features required for contact detection. Depth estimation is only activated when the processed signal exceeds a predefined threshold. Therefore, oscillations occurring outside the contact regime do not influence the estimation process. This is reflected in Fig. S7, where the depth estimates remain stable despite

Table S1: **Evaluation metrics for all models we tested across trials in dataset 1.** The table shows the MAE and RMSE for each model, across different trials and categories.

Model	**Whisker Left**		**Whisker Right**		**Orientation**		**Reconstruction**	
	MAE	RMSE	MAE	RMSE	MAE	RMSE	MAE	RMSE
**PolyFit**	9.07	12.03	10.41	13.19	7.04	8.43	8.72	12.10
Trial 1	10.04	11.98	10.68	13.96	9.63	10.30	8.32	12.28
Trial 2	10.26	13.50	9.90	13.14	2.42	3.11	9.50	13.11
Trial 3	6.71	10.25	10.70	12.35	9.31	10.03	8.28	10.66
**GPR**	8.69	10.39	9.51	12.28	9.21	10.88	7.51	10.29
Trial 1	11.43	12.48	9.34	12.84	13.76	14.62	7.90	10.59
Trial 2	8.07	10.13	9.62	12.44	4.94	6.78	7.63	10.80
Trial 3	6.42	7.90	9.56	11.45	8.98	9.78	6.96	9.32
**RFR**	7.83	10.61	6.93	9.63	7.31	9.78	6.75	9.29
Trial 1	9.24	11.41	7.73	9.91	10.99	13.64	6.54	8.37
Trial 2	6.41	8.85	7.16	10.42	5.53	6.89	6.61	9.07
Trial 3	7.87	11.46	5.81	8.33	5.31	7.14	7.13	10.41
**MLP**	6.40	9.07	5.36	8.08	6.18	7.72	5.34	8.06
Trial 1	4.63	5.86	7.23	9.53	8.79	10.17	4.74	6.68
Trial 2	7.96	11.39	4.90	7.86	4.76	5.87	6.31	9.45
Trial 3	6.59	9.05	3.84	6.46	4.91	6.31	4.91	7.76
**MLP + KF (Simplified)**	6.19	8.35	5.13	7.09	5.96	7.36	4.73	6.99
Trial 1	4.86	5.78	6.27	8.19	8.61	9.77	4.16	5.64
Trial 2	7.15	10.26	5.43	7.49	4.34	5.28	5.35	8.36
Trial 3	6.57	8.36	3.57	5.06	4.89	6.21	4.65	6.64
**MLP + KF (Full Model)**	**4.23**	**5.69**	**4.72**	**6.75**	**5.55**	**6.84**	**4.12**	**5.75**
Trial 1	3.44	4.25	8.53	10.12	8.30	9.22	5.93	7.20
Trial 2	5.00	6.95	3.71	4.87	3.60	4.44	3.70	5.51
Trial 3	4.23	5.50	1.72	2.89	4.73	5.95	2.63	3.96

Table S2: Hyperparameter search spaces and selected values for all compared sensor models. For all methods, hyperparameters were selected on the training set using 5-fold cross-validation.

**Model**	**Hyperparameter**	**Search range**	**Selected value**	
			**Left whisker**	**Right whisker**
PolyFit	Polynomial degree	$\{1, 2, \ldots, 10\}$	1	1
RFR	Number of trees	$\{100, 200, 300\}$	300	100
	Maximum tree depth	{None, 5, 10, 20}	5	5
	Minimum samples for split	$\{2, 5, 10\}$	5	10
	Minimum samples per leaf	$\{1, 2, 4\}$	2	4
GPR	Kernel amplitude c	$\{1, 2, \ldots, 10\}$	6	9
	RBF length scale l	$\{50, 100, \ldots, 10000\}$	2450	4600
MLP	Hidden units per layer	$\{16, 32\}$	(32, 32, 32)	(32, 32, 32)
	Batch size	$\{32, 64, 128\}$	32	32
	Learning rate	$\{10^{-4}, 10^{-3}\}$	10^{-3}	10^{-3}
	Dropout	$\{0, 0.1, 0.5\}$	0.1	0.1
	Regularization	$\{0, 10^{-4}, 10^{-3}, 10^{-2}\}$	10^{-3}	10^{-2}

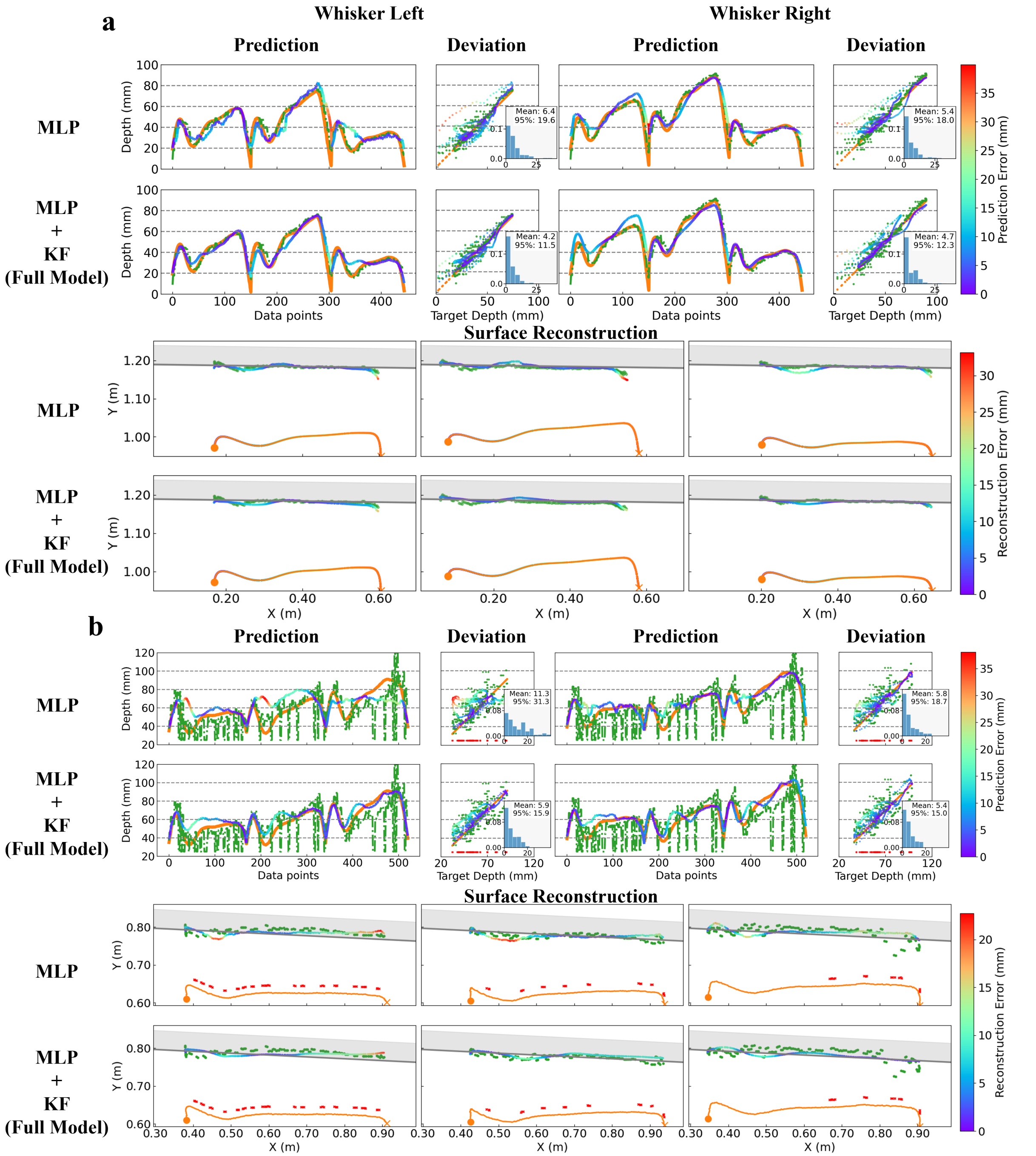

Figure S6: Additional details on whisker-based tactile depth estimation experiments. (a) Supplementary results on Dataset1, including the prediction vs. GT deviation plots. The bottom-right section of the figure presents a histogram illustrating the deviation distribution, along with the mean and 95% confidence interval. (b) Additional results on Dataset2, including deviation plots and surface reconstruction. We represent laser reconstruction points as green dots, failure detection as red crosses, whisker reconstruction points as rainbow-colored dots, and include reconstruction error bars.

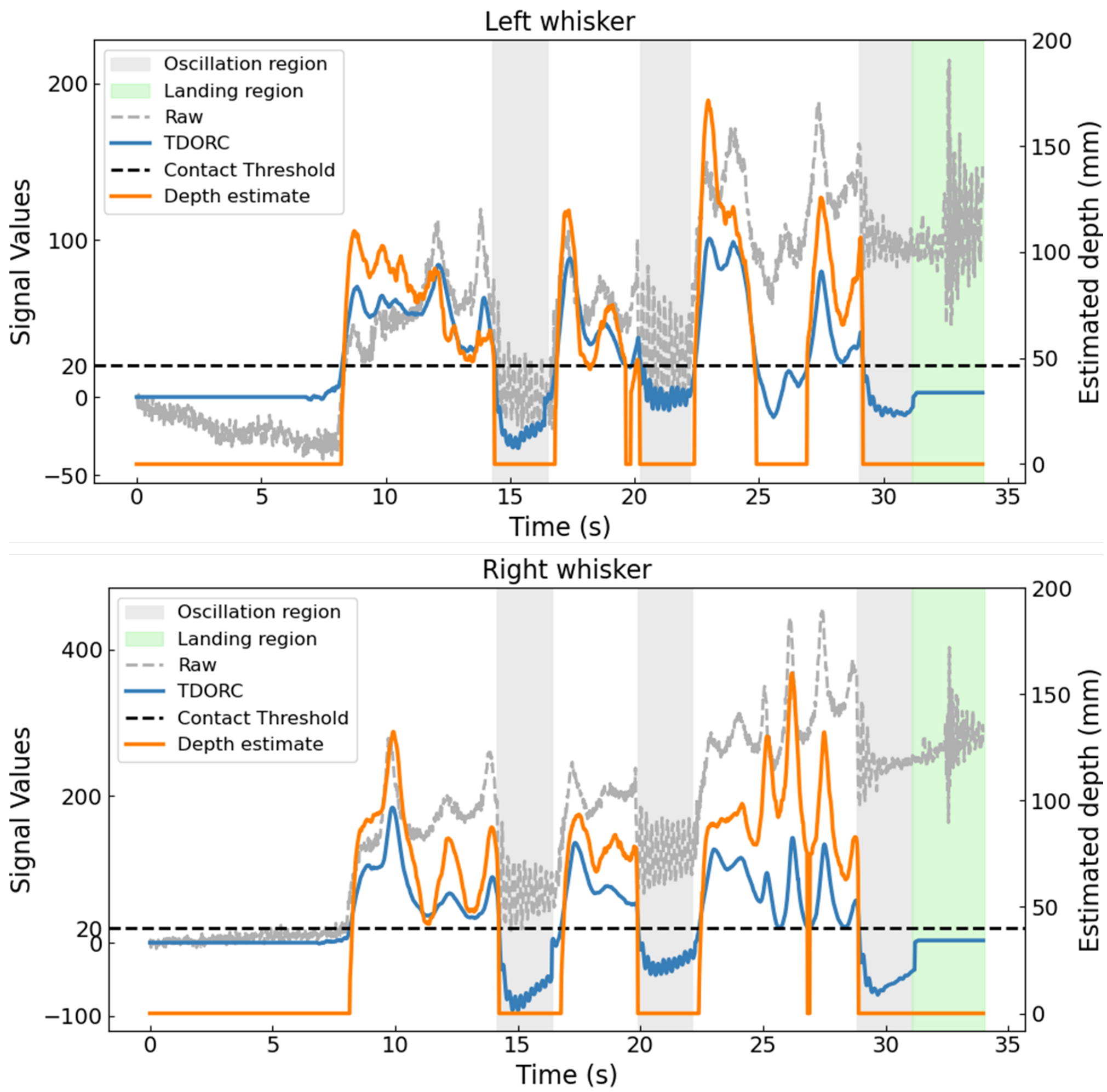

Figure S7: Whisker signal processing and depth estimation under post-contact oscillations. Raw whisker signals (gray), TDORC-processed signals (blue), contact threshold (black dashed line), and corresponding depth estimates (orange) for the left (top) and right (bottom) whiskers. The shaded gray regions indicate intervals with strong post-contact oscillations caused by whisker rebound and drone vibrations after sliding off the obstacle. The green region denotes the landing phase. For clarity of visualization, we show the top channel of each whisker, defined as the channel experiencing the largest deformation under contact and thus exhibiting the strongest response. This channel is also used for contact detection in our system.

strong oscillations in the raw signals. The green region denotes the landing phase, after which the navigation program terminates and the corresponding signals return to zero.

We further observe that the processed signals may exhibit small negative values immediately after contact is lost. This is due to the transient response of the band-pass filtering stage: when the signal undergoes a sudden drop (from contact to no-contact), the filter produces a short negative undershoot. This behavior is expected and does not affect subsequent processing. Since contact detection relies on a positive threshold applied to the processed signal, these transient values do not interfere with contact detection or the resulting depth estimation.

Mitigation of Post-Contact Recovery Delay

During aerial tactile navigation, the whisker signals are affected not only by high-frequency oscillations but also by slow recovery dynamics after contact is lost. This effect arises from the partial deformation and recovery of the soft gel material, leading to a delayed return of the signal to its baseline. Such behavior can introduce a residual bias that interferes with subsequent contact detection. To analyze this effect, we consider data from the aerial tactile navigation experiment (Fig. 6, Trial 4), specifically when the drone passes the first wall. Fig. S8 shows the signal from the top channel of the left whisker, which corresponds to the channel undergoing the largest deformation and thus providing the most prominent contact response. Similar behavior is observed across other channels. As shown in Fig. S8, the raw signal exhibits both noise and a clear delay in returning to zero after contact release. Removing the linear trend (detrended residual) reduces drift but does not resolve the slow recovery behavior. Applying low-pass filtering alone further smooths the signal but retains the low-frequency bias, resulting in a signal that remains offset during the hysteresis-dominated interval. In contrast, the proposed TDORC method effectively mitigates this delayed recovery. By combining online drift compensation with a band-pass filter, the method attenuates both high-frequency ringing and low-frequency recovery components. As a result, the TDORC-processed signal returns to baseline more rapidly after contact loss, reducing the impact of recovery-induced bias. It is important to note that this approach does not remove the intrinsic hysteresis of the sensing material during contact. Instead, it mitigates its effect on the signal during the transition from contact to free space. This is particularly important for reliable contact detection in aerial tactile navigation, where rapid transitions between contact and free flight frequently occur.

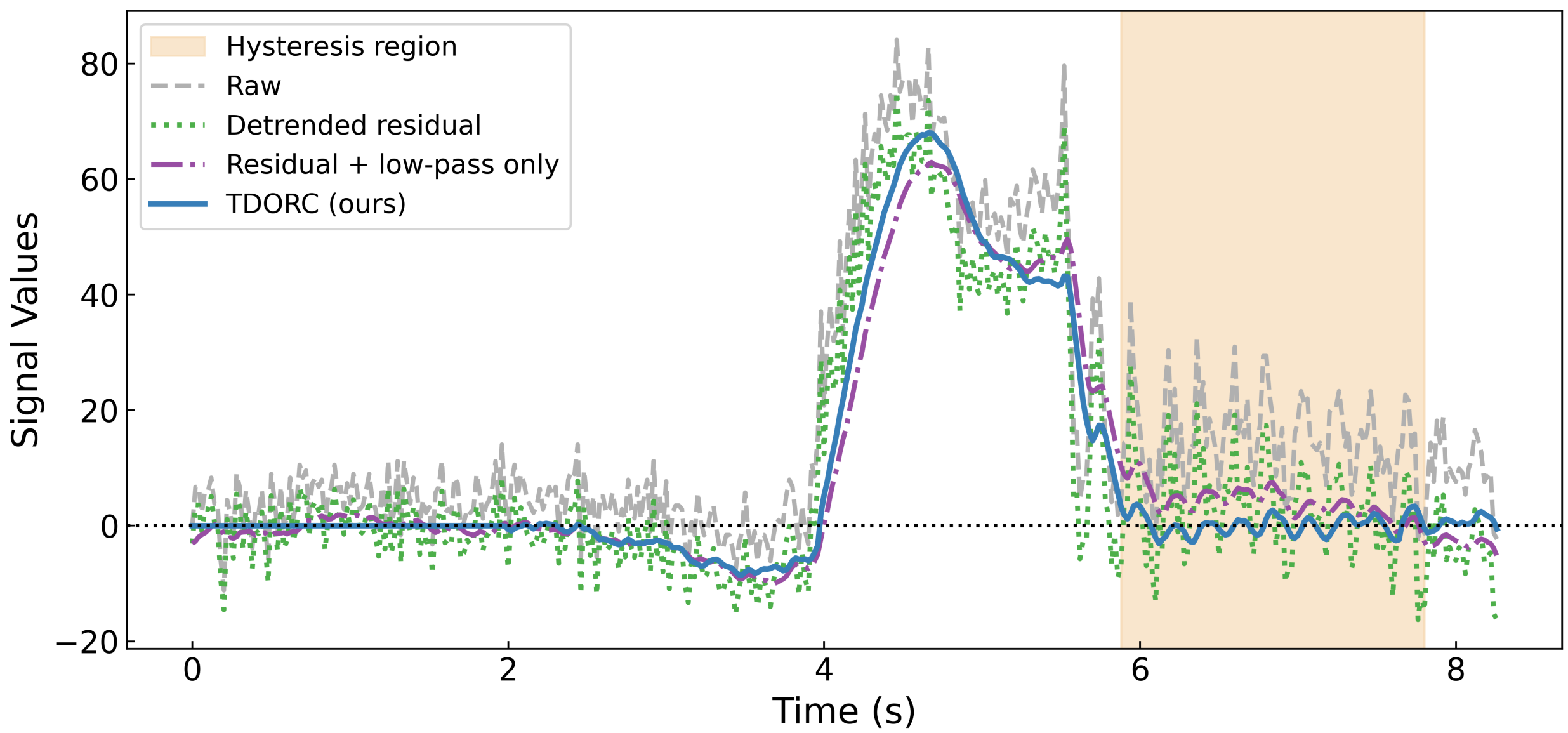

Figure S8: Comparison of the raw whisker signal (gray), detrended residual (green), residual with low-pass filtering only (purple), and the TDORC-processed signal (blue). The data are taken from the aerial tactile navigation experiment (Trial 4) when the drone passes the first wall. We show the top channel of the left whisker, i.e., the channel experiencing the largest deformation under contact and exhibiting the strongest response. The shaded region highlights the hysteresis-dominated interval after contact release, where the signal exhibits delayed recovery due to the soft material response. The low-pass-only signal remains biased and does not return to baseline, whereas TDORC effectively restores the signal to zero by attenuating low-frequency components associated with the recovery process. This behavior is critical for reliable contact detection.

Robustness to curved and moving surfaces

To evaluate the effect of violating the locally planar and static-obstacle assumptions used in the process model, we conducted additional wall-following experiments on curved surfaces and a moving surface, as shown in Fig. S9 and Supplementary Movie 8.

For the curved-surface experiments, we used three transparent acrylic surfaces with different curvatures: a concave surface with relatively low curvature, a concave surface with higher curvature, and a convex surface with higher curvature. In each case, the drone performed 10 s of wall-following sweeping using the same finite-state-machine (FSM)-based aerial tactile navigation framework as in the *Aerial tactile navigation via invisible walls* section (Fig. S5). Fig. S9a–c shows representative image sequences of these three experiments. The corresponding top-view reconstructions are shown in Fig. S9d–f. In each plot, we visualize the drone trajectory and orientation together with the reconstructed surface and the approximate surface. The reconstructed surface is obtained by averaging the left and right whisker depth estimates from the MLP+KF model. The approximate surface is obtained by fitting a cubic spline to control points recorded by the OptiTrack system. These results show that the current system can still follow curved surfaces and recover approximate local geometry. In the initial stable wall-following stage, the reconstruction remains relatively accurate. However, as curvature increases and the interaction becomes more dynamic, oscillatory motion emerges, and the reconstruction quality degrades noticeably during the oscillatory phase.

In the low-curvature concave case and the high-curvature convex case, oscillatory motion appears near the end of the trial, as visible in Fig. S9a,c and Supplementary Movie 8. A likely cause is the current FSM controller, which does not regulate the contact state as smoothly as a continuous feedback controller. Once the drone moves outside the threshold band, it tends to oscillate around the upper and lower bounds instead of returning smoothly to the desired band. Under these higher-dynamic conditions, tactile perception also becomes less reliable, which further degrades reconstruction quality. Nevertheless, the system remains capable of completing curved-surface wall following under these conditions.

We further evaluated the system on a moving surface, as shown in Fig. S9g and Supplementary Movie 8. Although the controller again exhibits oscillatory behavior similar to that observed in the curved-surface experiments, the drone still successfully follows the moving obstacle and continues

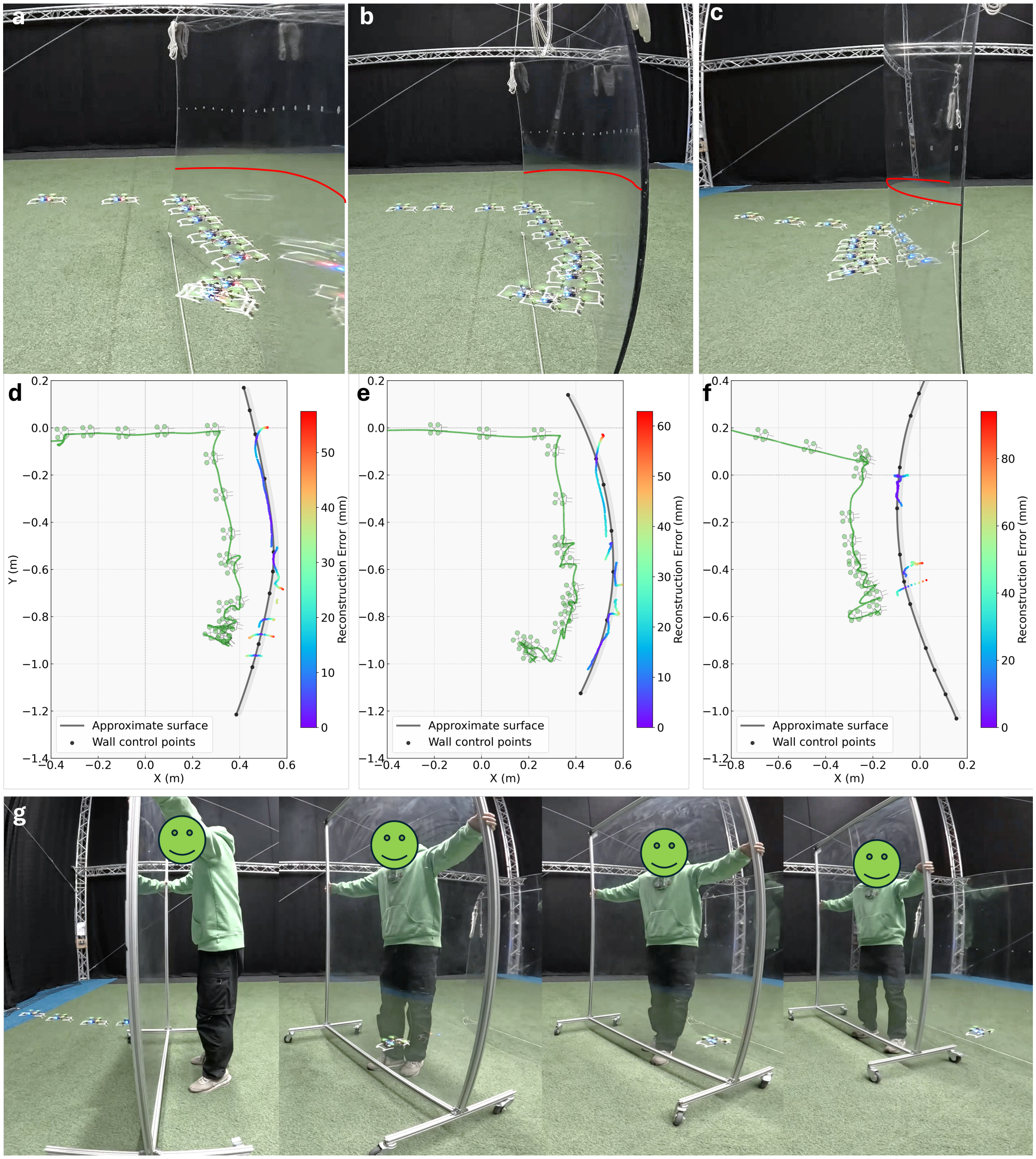

Figure S9: Experimental results of aerial tactile navigation on curved and moving surfaces. (a–c) Whiskered drone performing 10 s wall-following sweeping on a low-curvature transparent concave surface, a high-curvature transparent concave surface, and a high-curvature transparent convex surface, respectively. (d–f) Corresponding top-view trajectories and surface reconstructions for the three curved-surface experiments. The reconstructed surface is obtained from the average of the left and right whisker estimates using the MLP+KF model, and the approximate surface (GT) is obtained by fitting a cubic spline to OptiTrack control points. (g) Whiskered drone performing wall following on a moving surface.

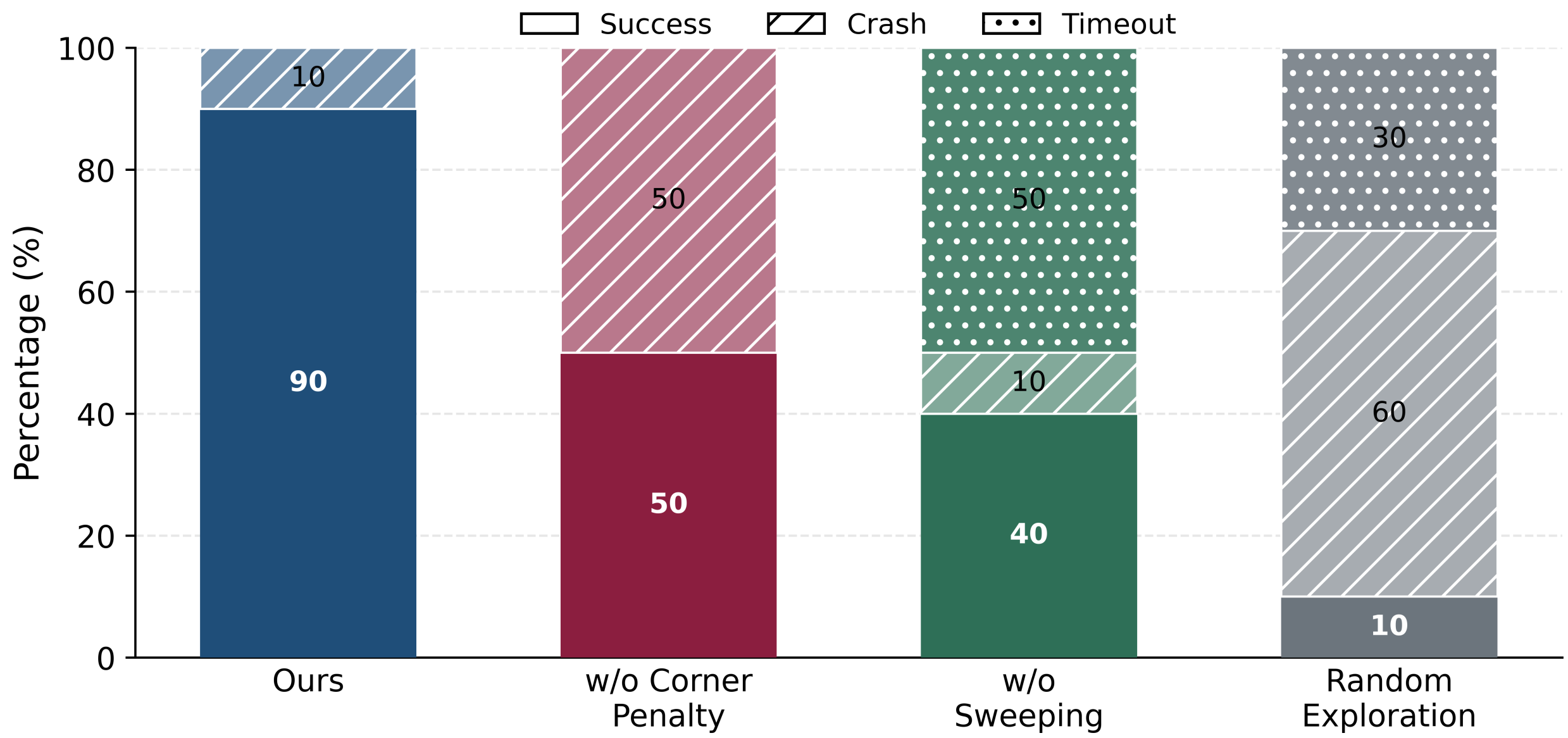

Figure S10: Failure modes across tactile exploration methods. Removing the corner-penalty module leads to a substantial increase in failures due to crashes. In contrast, removing the sweeping behaviour results in more timeout cases due to insufficient exploration. Random exploration exhibits both frequent crashes and timeout events.

forward motion around it.

These experiments indicate that the current system remains usable under moderate violations of the static and locally planar assumptions, but that stronger curvature and obstacle motion increase controller–perception coupling and degrade performance. Future work will investigate whisker-based tactile servoing to achieve more stable curved-surface tracking and tighter integration of tactile perception and control.

Ablation study on active aerial tactile exploration

Fig. S10 shows the breakdown of trial outcomes into success, crashing, and timeout events across different methods.

Fig. S11 presents qualitative results for all trials, including drone trajectories, reconstructed contours, GT layouts, and failure cases (red crosses indicate crashing events).

These results further corroborate the distinct roles of each component in shaping tactile flight exploration performance and failure modes.

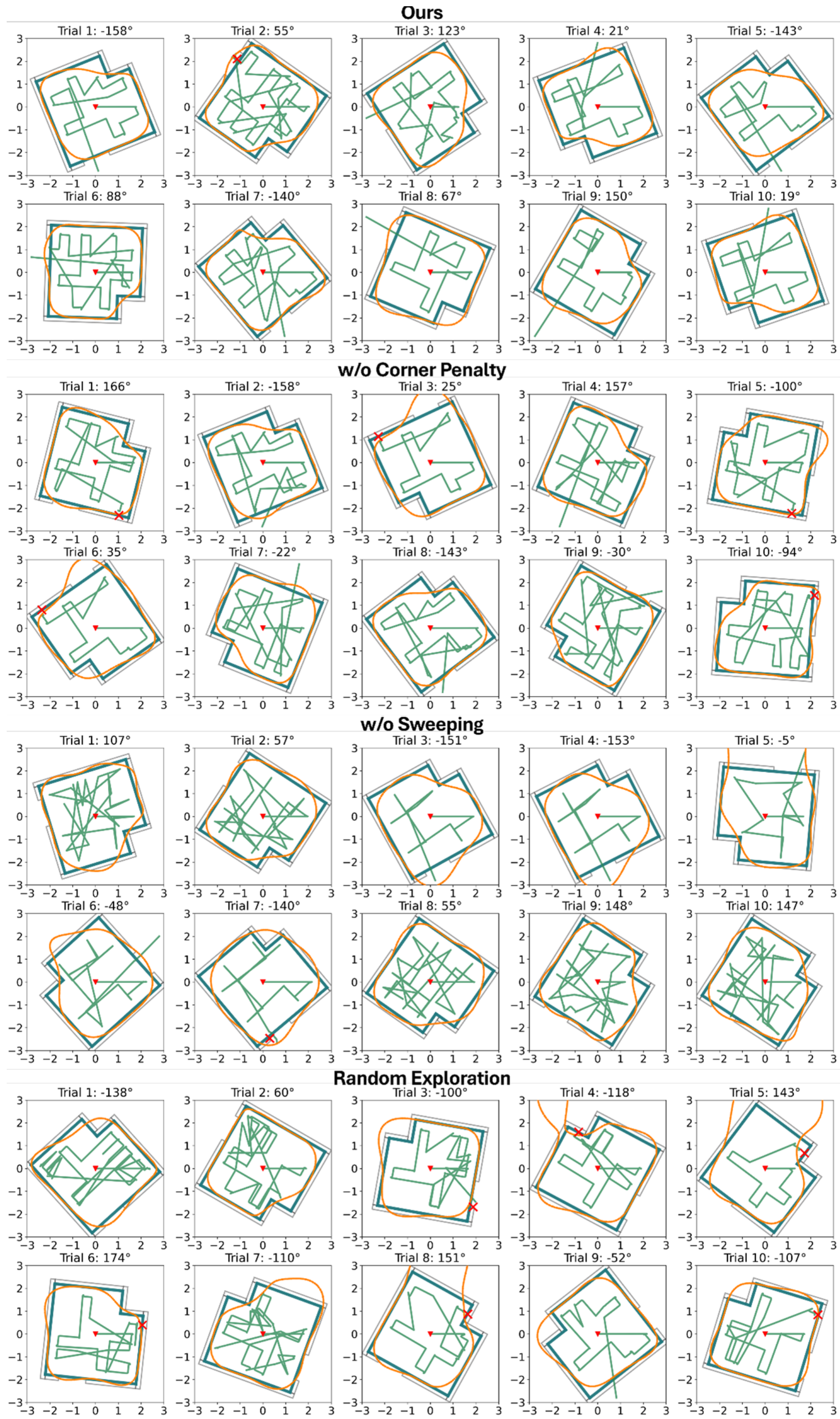

Figure S11: Qualitative results across all trials. Each subplot shows the drone trajectory (green), reconstructed contour (orange), and GT layout (blue). Crash cases are indicated by red crosses. Without the corner-penalty module, the drone frequently enters concave regions and collides with sharp corners. In contrast, the proposed method consistently avoids these regions and produces stable exploration trajectories.

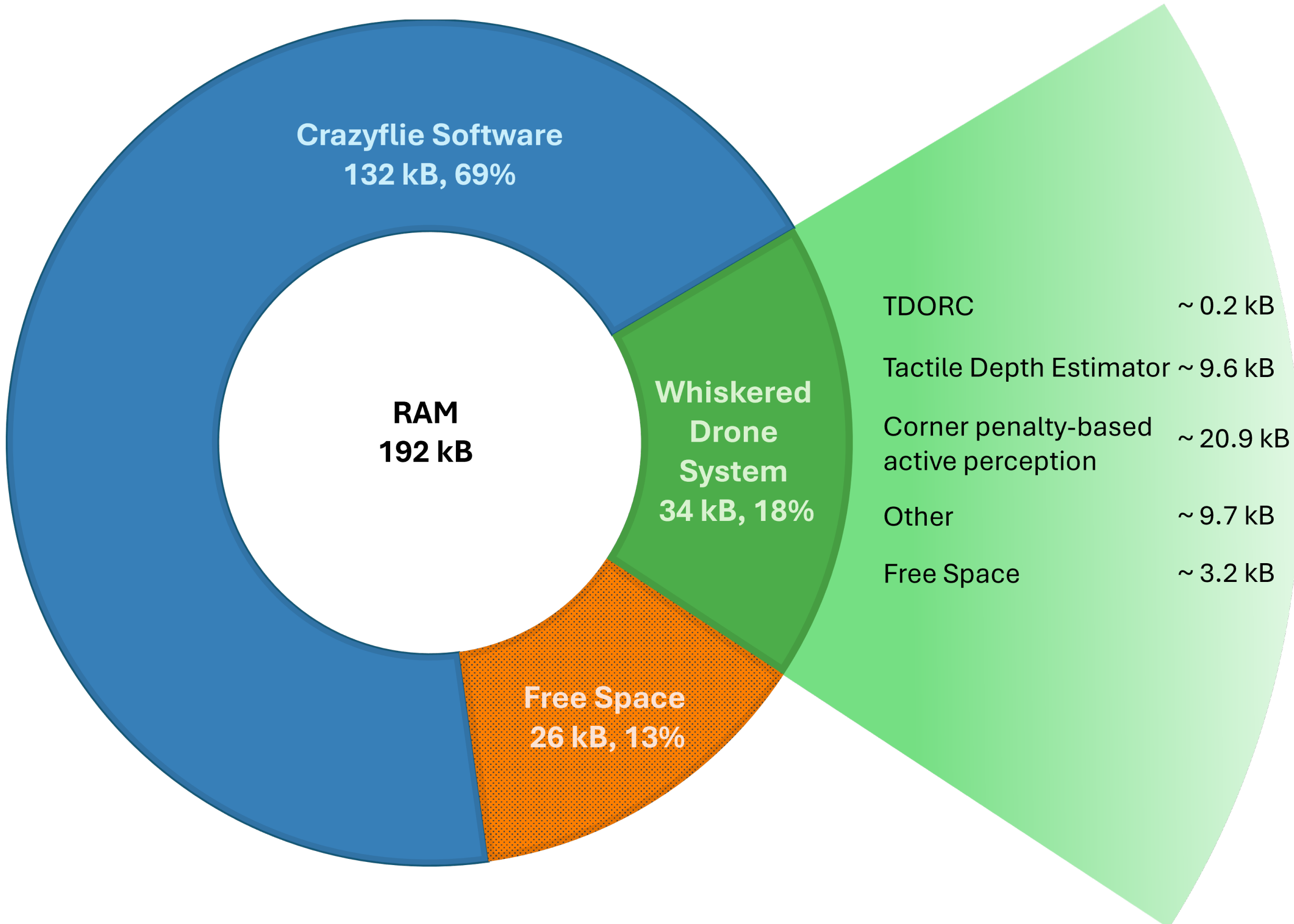

Figure S12: Crazyflie memory usage breakdown. Total free space: 26 kB.

Memory usage and computational cost

We use an STM32F405 microcontroller with 192 kB of RAM, of which the base Crazyflie firmware occupies approximately 132 kB (Fig. S12). The whisker-based autonomous exploration system uses about 34 kB of additional memory. Within this budget, the Tactile Drift Online Recurrent Compensation (TDORC) module requires approximately 0.2 kB, while the tactile depth estimator occupies around 9.6 kB, including the neural network and Kalman filter (KF) fusion parameters. Since the two whisker-based depth estimators are cross-called, only one instance of this memory is required.

For the GPIS-based active perception module, we limit the system to 50 training data points and a 10×10 grid representation. Under this configuration, the GPIS module requires approximately 20.9 kB of memory, which is sufficient for reconstructing small-scale environments such as indoor rooms. Importantly, the penalty-based active perception module and the tactile depth estimator are

not executed simultaneously. Their temporary working memory can therefore be reused rather than allocated concurrently, improving overall memory efficiency.

Under the current configuration, the system remains within the onboard memory budget and retains approximately 26 kB of free RAM. When running aerial tactile exploration without the active perception module, an additional 11.3 kB of memory is available.

In addition to memory usage, we evaluate the computational cost of the GPIS module. Because the system is constrained to a bounded dataset (maximum 50 points), the resulting kernel matrices remain small and enable efficient onboard computation. On the STM32F405, the combined GPIS update (training and inference) takes approximately 0.09 s for 20 points, 0.14 s for 30 points, 0.21 s for 40 points, and 0.29 s for 50 points. In practice, GPIS updates are not executed at every control cycle. The full onboard exploration pipeline runs at 50 Hz, while GPIS is triggered intermittently during active exploration. This asynchronous execution keeps the computational load compatible with real-time onboard operation.

Flight Time Analysis

We provide a detailed analysis of the impact of the whisker sensing system and onboard computation on flight time. Three configurations are evaluated: (1) no extra payload, (2) whisker suite including wiring and propeller guard with data transmission, and (3) whiskers with the full onboard tactile perception pipeline.

The measured flight times are 11.26 min, 8.52 min, and 7.18 min, respectively (Fig. S13), corresponding to reductions of approximately 24% and 36% compared to the no-extra-payload baseline.

Importantly, the tactile perception pipeline is not continuously active. Instead, it is triggered intermittently upon contact events. This event-driven design reduces unnecessary computation and helps limit the overall energy consumption on the tiny drone.

The reduction in flight time is therefore mainly attributed to the additional payload (whisker structure, wiring, and protection) and the intermittent activation of onboard processing. Despite this trade-off, the system remains capable of fully onboard tactile perception and navigation within the tight mass constraints of a 44.1 g nano drone.

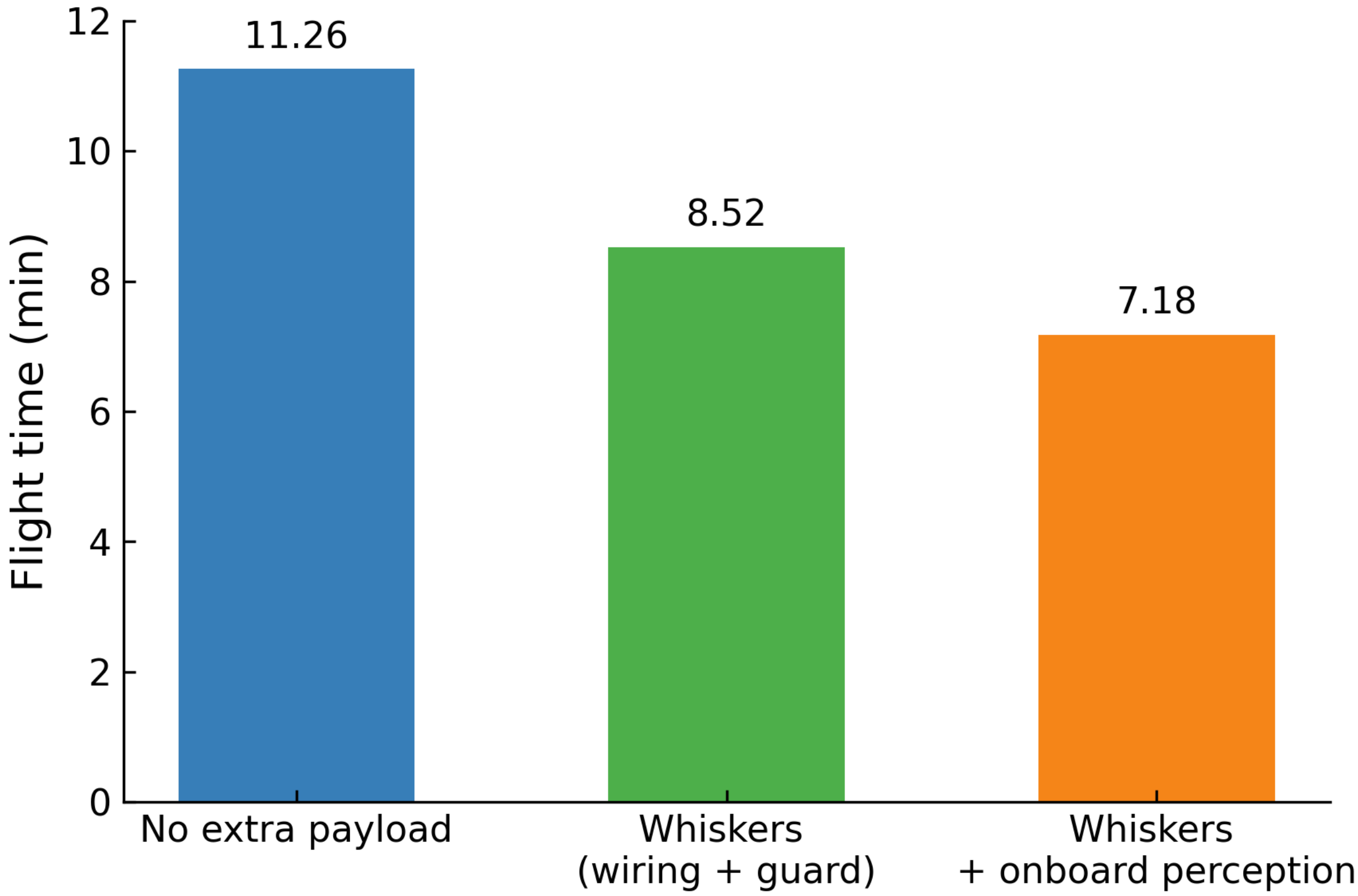

Figure S13: Flight time under different configurations. Comparison of flight time for (i) no extra payload, (ii) whisker suite with wiring and propeller guard, and (iii) whiskers with onboard tactile perception. The perception pipeline is activated intermittently upon contact events (every 30 s).

Comparison with collision-resilient aerial systems

Table S3 summarizes key interface-level differences between the proposed whisker-based system and representative collision-resilient aerial platforms. XPLORER (*9*) and RMF (*8*) also exploit continuous contact information. However, compared with the present work, their sensing range is much shorter, since contact information is obtained through body or flap deformation rather than through forward-reaching whiskers. In addition, these systems generally involve larger interaction forces, larger contact areas, larger structural footprint, and substantially heavier platforms. Notably, lighter prior systems at 133 g and 321 g provide only binary collision-event sensing, whereas the systems that exploit continuous contact information are substantially heavier, at 495 g and 1.12 kg, compared with 44.1 g in this work. These differences lead to qualitatively different interaction mechanics for tactile wall following. In particular, our whisker-placement experiments already show that even modest changes in contact-induced moment can strongly affect flight stability on a very small aerial platform. Hence, we expect that bumper-based systems with a large contact area will experience large, destabilizing contact-induced moments. In contrast, the proposed system provides localized continuous tactile sensing with a longer sensing range and very low interaction force, which are particularly suited to active tactile sweeping and wall following on a tiny drone. Among the compared systems, XPLORER is the closest in spirit in that it supports sustained contact and sweeping-like interaction, but it does so through body deformation and force estimation on a much heavier deformable platform. RMF, while equipped with continuous contact sensing, primarily relies on laser/ToF-based obstacle avoidance rather than tactile sensing as the principal navigation modality. These differences highlight that the compared systems are not directly equivalent benchmarks for the tactile sensing problem addressed in this work.

Table S3: **Interface-level comparison between whisker-based tactile sensing and representative collision-resilient drones equipped with mechanical bumpers.** The table highlights contact-interface properties relevant to tactile wall following, rather than providing a direct performance benchmark, since the platforms, tasks, and sensing goals differ substantially across studies.

System	Weight	Tactile/contact information	Interaction force	Supports active tactile sweeping	Sensing range
**This work**	**44.1 g**	**Localized continuous tactile sensing**	**Very Low**	**Yes**	**Large (whisker deformation)**
RMF with laser avoidance (*8*)	495 g	Flexion-based continuous contact sensing	Low	No	Moderate (flap deformation)
XPLORER (*9*)	1.12 kg	Force-based continuous tactile sensing	High (body contact)	Yes	Very short (body deformation)
(*10*)	321 g	Discrete collision event	High (body contact)	No	None
Tiercel (*11*)	133 g	Discrete collision events	High (body contact)	No	None
Air Bumper (*12*)	1.45 kg	Discrete collision events	High (body contact)	No	None

References

1. C. Ye, G. De Croon, S. Hamaza, A Biomorphic Whisker Sensor for Aerial Tactile Applications, in *2024 IEEE International Conference on Robotics and Automation (ICRA)* (IEEE) (2024), pp. 5257–5263.

2. T. A. Kent, *et al.*, Whisksight: A reconfigurable, vision-based, optical whisker sensing array for simultaneous contact, airflow, and inertia stimulus detection. *IEEE Robotics and Automation Letters* **6** (2), 3357–3364 (2021).

3. T. A. Kent, H. Emnett, M. Babaei, M. J. Hartmann, S. Bergbreiter, Identifying contact distance uncertainty in whisker sensing with tapered, flexible whiskers, in *2023 IEEE International Conference on Robotics and Automation (ICRA)* (IEEE) (2023), pp. 607–613.

4. S. Kim, C. Velez, D. K. Patel, S. Bergbreiter, A magnetically transduced whisker for angular displacement and moment sensing, in *2019 IEEE/RSJ International Conference on Intelligent Robots and Systems (IROS)* (IEEE) (2019), pp. 665–671.

5. S. Kim, *et al.*, A whisker-inspired fin sensor for multi-directional airflow sensing, in *2020 IEEE/RSJ International Conference on Intelligent Robots and Systems (IROS)* (IEEE) (2020), pp. 1330–1337.

6. M. A. Lin, E. Reyes, J. Bohg, M. R. Cutkosky, Whisker-inspired tactile sensing for contact localization on robot manipulators, in *2022 IEEE/RSJ International Conference on Intelligent Robots and Systems (IROS)* (IEEE) (2022), pp. 7817–7824.

7. J. H. Solomon, M. J. Hartmann, Extracting object contours with the sweep of a robotic whisker using torque information. *The International Journal of Robotics Research* **29** (9), 1233–1245 (2010).

8. P. De Petris, H. Nguyen, M. Kulkarni, F. Mascarich, K. Alexis, Resilient collision-tolerant navigation in confined environments, in *2021 IEEE International Conference on Robotics and Automation (ICRA)* (IEEE) (2021), pp. 2286–2292.

9. K. Patnaik, A. A. P. Saravanakumaran, W. Zhang, Tactile-Based Exploration, Mapping, and Navigation With Collision-Resilient Aerial Vehicles. *IEEE/ASME Transactions on Mechatronics* (2025).

10. A. Bredenbeck, T. Yang, S. Hamaza, M. W. Mueller, A Tactile Feedback Approach to Path Recovery after High-Speed Impacts for Collision-Resilient Drones. *arXiv preprint arXiv:2410.14249* (2024).

11. Y. Mulgaonkar, *et al.*, The tiercel: A novel autonomous micro aerial vehicle that can map the environment by flying into obstacles, in *2020 IEEE International Conference on Robotics and Automation (ICRA)* (IEEE) (2020), pp. 7448–7454.

12. R. Wang, Z. Guo, Y. Chen, X. Wang, B. M. Chen, Air Bumper: A Collision Detection and Reaction Framework for Autonomous MAV Navigation, in *2024 IEEE International Conference on Robotics and Automation (ICRA)* (IEEE) (2024), pp. 15735–15741.
