# Supplementary material for: Whisker-based tactile flight for tiny drones
Source: Nat Commun. 2026 Sep 18;17:9743. doi: 10.1038/s41467-026-77366-7 (PMC13588785; doi:10.1038/s41467-026-77366-7)
Supplement: Supplementary file 3 [file 41467_2026_77366_MOESM2_ESM.pdf]

Description of Additional Supplementary Files

Supplementary Movie 1. Placement-angle experiments on the Crazyflie 2.1 Brushless platform.

Supplementary Movie 2. Tactile drift online recurrent compensation during free-flight experiments.

Supplementary Movie 3. Data collection and test-set inference for whisker-based tactile depth estimation.

Supplementary Movie 4. Aerial tactile navigation through invisible walls.

Supplementary Movie 5. Active aerial tactile exploration in unknown confined environments.

Supplementary Movie 6. Aerial tactile navigation in complete darkness.

Supplementary Movie 7. Supplementary placement-angle experiments on the Crazyflie 2.1 brushed platform.

Supplementary Movie 8. Aerial tactile navigation on curved and moving surfaces.

Supplementary Data S1. Experimental data supporting the figures, tables, and supplementary analyses.
